# A Bioinformatics Classifier and Database for Heme-Copper Oxygen Reductases

**DOI:** 10.1371/journal.pone.0019117

**Published:** 2011-04-29

**Authors:** Filipa L. Sousa, Renato J. Alves, José B. Pereira-Leal, Miguel Teixeira, Manuela M. Pereira

**Affiliations:** 1 Instituto de Tecnologia Química e Biológica, Universidade Nova de Lisboa, Oeiras, Portugal; 2 Instituto Gulbenkian de Ciência, Oeiras, Portugal; National Institute for Medical Research, Medical Research Council, London, United Kingdom

## Abstract

**Background:**

Heme-copper oxygen reductases (HCOs) are the last enzymatic complexes of most aerobic respiratory chains, reducing dioxygen to water and translocating up to four protons across the inner mitochondrial membrane (eukaryotes) or cytoplasmatic membrane (prokaryotes). The number of completely sequenced genomes is expanding exponentially, and concomitantly, the number and taxonomic distribution of HCO sequences. These enzymes were initially classified into three different types being this classification recently challenged.

**Methodology:**

We reanalyzed the classification scheme and developed a new bioinformatics classifier for the HCO and Nitric oxide reductases (NOR), which we benchmark against a manually derived gold standard sequence set. It is able to classify any given sequence of subunit I from HCO and NOR with a global recall and precision both of 99.8%. We use this tool to classify this protein family in 552 completely sequenced genomes.

**Conclusions:**

We concluded that the new and broader data set supports three functional and evolutionary groups of HCOs. Homology between NORs and HCOs is shown and NORs closest relationship with C Type HCOs demonstrated. We established and made available a classification web tool and an integrated Heme-Copper Oxygen reductase and NOR protein database (www.evocell.org/hco).

## Introduction

Heme-copper Oxygen reductases (HCOs) are the main enzymes responsible for reduction of oxygen to water in respiratory chains. These membrane-bound enzymes, present in the three domains of life, Bacteria, Archaea and Eukarya, catalyze the last reaction of aerobic respiratory chains. HCOs contribute to energy conservation by two processes: i) charge separation, since protons and electrons needed for the chemical reaction come from opposite sides of the membrane, and ii) proton translocation, as part of the energy released during the O_2_ reduction is used to promote thermodynamically unfavorable proton translocation across the membrane. HCOs are composed of 3–4 (prokaryotes) and up to 13 subunits (eukaryotes) of which only the catalytic subunit I is common to all HCOs. Subunit I contains at least 12 transmembrane helices and has as cofactors, a low-spin heme, a binuclear centre (high-spin heme and Cu_B_ ion) where the reduction of O_2_ occurs, and a tyrosine residue covalently bound to a histidine residue ligand of the Cu_B_ ion (for review see [1], [2], [3], [4]). This tyrosine is proposed to be the source of the fourth electron needed for O_2_ reduction in HCOs [5], [6].

**Figure 1:**
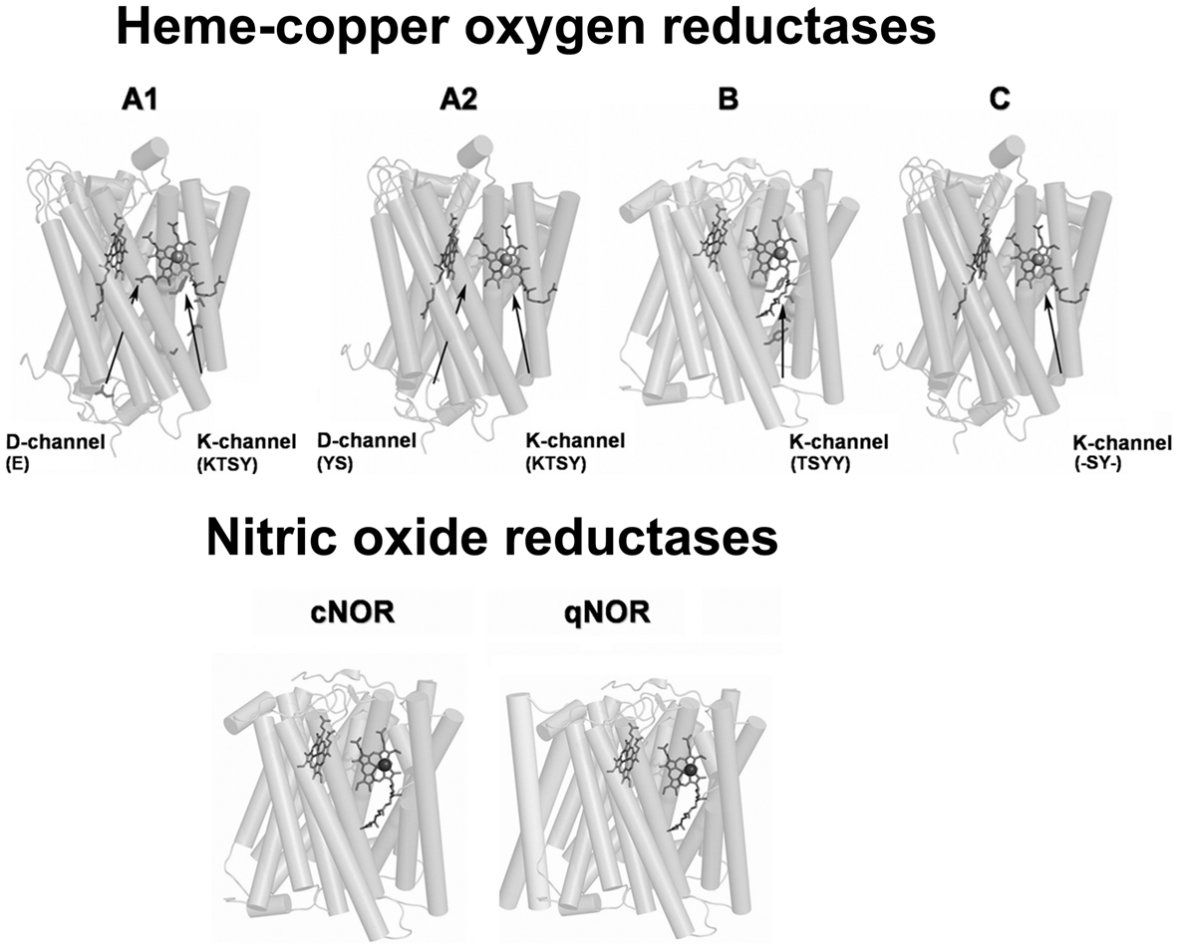
Cartoon of tertiary structures of the subunits I of different HCO type families and NORs. The black arrows indicate the localization of the proton conducting channels and the respective conserved amino acid residues are indicated between brackets. Heme groups are depicted as grey sticks, copper ions as grey spheres and iron ions as black spheres. With exception of qNOR, all other proteins have deposited pdb files.

Sequence alignments, site directed mutagenesis and X-ray crystallographic structural models of subunit I led to the identification of intra-protein proton conducting channels in the mitochondrial and mitochondrial-like enzymes [7], [8], [9], [10]. These channels were named D and K due to specific amino acid residues considered to play an important role in proton translocation (see for instance [11], [12]). As more amino acid sequences of HCOs became available, the residues lining the proton conducting channels were observed not to be fully conserved. Indeed, analysis of subunit I from several HCOs, namely including those from organisms from diverse taxonomic groups, led to the identification of distinct patterns of possible proton channels. These patterns became the basis for the classification of HCOs into three types, proposed by Pereira and co-workers: A (subdivided into A1 and A2), B and C (Figure 1) [13]. Recently, also based on sequences comparisons, an extension of this classification, including the existence of new types (D to H) was suggested [14].

Nitric oxide reductases (NORs) were previously proposed to be included in the HCO superfamily, based on the predicted structural similarity to heme-copper oxygen reductases, [15], [16], [17]. Recently, the structure of a cNOR was determined showing that the catalytic subunit of these enzymes, shares with subunit I of HCOs the same structural fold core of 12 transmembrane helices, and the presence of the low-spin heme and the binuclear center, in this case formed by the high-spin heme and an iron ion, instead of Cu_B_ [18]. The cross-linked tyrosine residue is absent in NORs and is therefore considered to be a distinctive feature between HCOs and NORs. In A and in the majority of B Type enzymes, this cross-linked tyrosine residue is located in helix VI (referred as Tyr-I), whereas in C Type enzymes it is present in helix VII (Tyr-II) [6], [19]. Moreover, in contrast to what is observed for HCOs, no proton channel (nor hydrogen-bonding network) from the cytoplasmic region to the active site of the cNOR was identified in the structure of the cNOR[18]. This is in agreement with the experimental results which showed the non-electrogenicy of the enzyme [20], [21], [22]. Data on qNOR are even more scarce, since no pdb file is available neither experimental results concerning proton uptake or translocation.

Cytochrome *bd* oxidase and the so-called alternative oxidase are also membrane-bound oxygen reductases [23], [24]. However, these enzymes are structurally unrelated to each other and to HCOs, which suggests distinct and independent evolutionary processes, and thus are not included in the present study.

The high increase of completely sequenced genomes represented an opportunity to clarify the classification of HCO enzymes and understand their evolutionary relationship with NORs. Commonly used methods based on local similarities such as BLASTP [25], or domain-based methods like superfamily or Pfam [26], [27] yield reasonable good results in predicting the superfamily and conserved domains of HCOs and NORs. However, manual decisions are required to classify these proteins to any of the functional classes, which are unattainable in face of the large and increasing number of HCO and NOR sequences available.

Here we report a reassessment of the functional types within this family and the establishmentof NOR as a homolog of HCOs based on the comparison between their respective subunit I. The existence of cytochromes *c* domains in the equivalent subunit II of C Type enzymes and cNORs could indicate a closer relationship between these two enzymes, and thus, justify the inclusion of subunit II for the classification analysis of HCOs and NORs. However, the presence of cytochrome *c* domains in subunit II is not exclusive of C Type enzymes or cNORs and results from amino acid sequence comparisons of the subunit II from the different HCO types indicate that their similarity is not related with enzyme type [28].

In this study, we further developed an automated, bioinformatics pipeline to identify and classify HCOs and NORs which we make available as a web tool. We used this pipeline to identify and classify HCO and NOR repertoires in 552 completely sequenced prokaryote genomes, which we also make available as a publicly accessible database.

## Methods

### Database searches

To compile a gold standard set of sequences we used representative sequences from the previously described enzyme types to query the protein non-redundant database at NCBI (A1 Type: *Paracoccus denitrificans aa*_3_, accession: CAA29274.1; A2 Type: *Rhodothermus marinus caa*_3_, accession: CAC08532.1; B Type: *Thermus thermophilus ba*_3_, accession: AAB00370.1; C Type: *Vibrio cholerae O395 cbb*_3_, accession: YP_001216999.1; NOR: *Paracoccus denitrificans PD1222* NOR, accession: YP_916266.1). Several iterative BLASTP queries were run with default parameters (Expected value:10, Word size 3, Blosum62, Gap Opening Penalty 11, Gap Extension Penalty 1 and Conditional compositional score matrix adjustments) using the last hit from each query as input for the next run until no new sequences were retrieved. We discarded duplicated sequences, those with less than 400 amino acid residues, and those in which the six conserved histidine residues ligands of Cu_B_ (in HCO) or Fe (in NORs), or of the low- and high-spin hemes were absent. With one exception, described in the results section, only prokaryotic sequences were considered.

A second data set, named Genomes data set, was built using Superfamily automated genome-wide domain assignments. The Superfamily database compiles structural domain assignments that are based on Hidden Markov Model profiles derived from known protein structures. The Superfamily accession code of the family considered was Cytochrome *c* oxidase subunit I-like SCOP Superfamily (code 81442), from the Superfamily ftp database (by April 2009) (available to download at http://supfam.mrc-lmb.cam.ac.uk/SUPERFAMILY/) [26]. The same two criteria, i.e., a defined sequence length and presence of conserved histidine residues as in the construction of the Gold Standard data set were used to accept each sequence.

All retrieved sequences were mapped on NCBI Taxonomy using the BioSQL package from April 2009 available to download at ftp://ftp.ncbi.nih.gov/. Sequence annotations were obtained from the following resources: RefSeq, Accession, GI, Uniprot, Kegg, Superfamily, Pfam and Prosite [26], [27], [29], [30], [31], [32]. The accession numbers of the sequences used in this study as well as their taxonomic classification are listed as Supporting Information in **Table S1**. Alignments are available upon request.

### Protein alignments

A global amino acid sequence alignment was performed with the Gold Standard data set in ClustalX v.1.83 [33]. Default parameters were used, as no significant differences were observed with different parameter combinations. Protein weight matrix Gonnet, with Gap Opening 10 and Gap Extension 0.2 was used for multiple alignments that were manually refined in GeneDoc v.2.7.0 [34]. From this global alignment, and using the defining fingerprints of the different types (see below) [13], [17], the Gold Standard sequences were manually classified as members of each group. In order to avoid redundancy of the data set, where multiple sequences exist for strains of the same species, only one sequence of the same type was considered (Table 1).

**Table 1:** Number of HCO and NOR sequences or species in the Gold Standard data set organized by taxonomic domains and enzyme type.

	Number of HCO/NOR sequences									
	total	Bacteria	Archaea	Eukarya	A1	A2	B	C	NOR	
Gold Standard data set	1216	1163	52	1	636	94	64	265	157	

	Number of species containing HCO/NORs									
	total	strains	Bacteria	Archaea	Eukarya	A1	A2	B	C	NOR
Gold Standard data set	524	553	502	21	1	392	66	51	230	139

### Phylogenetic analysis

Neighbour Joining (NJ) trees were constructed in ClustalX v.1.83 using the manually adjusted alignments with the following parameters: 10000 bootstraps, 1000 seeds and correction for multiple substitutions. Bayesian phylogenetic analysis was performed using Parallel Metropolis-coupled Markov chain Monte Carlo algorithm [35] in Mr.Bayes v.3.1.2 [36]. A likelihood model with two substitutions (nst = 2) and a gamma distribution were employed to distinguish between transitions and transversions and to account for different rates at different sites in the sequence. The initial parameters were 100000 generations (ngen =  100000) with trees saved every 1000 round (samplefreq = 1000) and four parallel chains to find additional peaks in the probability distribution (nchains = 4). Additional generations were performed until the average standard deviation of split frequencies was less than 0.01. A majority consensus tree was created and visualized in Dendroscope v2.4 [37].

### Validation of the Gold Standard data set and construction of the similarity network

In order to test the criteria employed for the sequence's manual classification, an all-versus-all blast was performed, in which each Gold Standard sequence was blasted against the Gold Standard data set using BLAST2P search (version 2.2.10, [25]) with standard parameters (Blosum62, Gap open penalty: −1, Gap extension penalty: −1, Expected value: 10). From these local alignments, a similarity score between each pair was calculated using the number of positives and the length of the alignment of the query sequence. When the similarity score between x-y was different from the y-x one, the mean value was used. A similarity matrix was created with all the scores and a representation of this network was generated in Cytoscape v2.6.1 [38] using the weighted spring-embedded layout which is based on a “force-directed” paradigm as implemented by Kamada and Kawai [39]. The network nodes are treated as physical objects that repel each other while the connections between nodes are treated like metal springs attached to the pair of nodes. These springs repel or attract their end points according to a force function. The layout algorithm sets the positions of the nodes in a way that minimizes the sum of forces in the network. Thus, the sequences (nodes) are spatially organized according to the sequence similarities (edges). The closer the sequences are located, the higher their similarity. As all possible relationships are taken into account, the formation of clusters and the number of linkages between them reflect the existence of naturally related groups containing those sequences. Average similarities of each cluster were calculated as arithmetic means using the pairwise similarity scores between each type of sequences. The retrieved natural groups were compared with the manual classification. The latter was considered valid if the result was in agreement with the natural groups obtained using the similarity matrix. The sequence properties such as enzyme type or taxonomic information are not taken into account for the network construction.

### Automated HCO classification

A leave-one-out cross validation was performed excluding each one of the Gold Standard data set sequences and blasting it against the remaining sequences using BLAST2P search (version 2.2.10,[25]) with default parameters (Blosum62, Gap open penalty: −1, Gap extension penalty: −1, Expected value: 10). Several tests of classification were performed based on i) the first (single blast hit), ii) the 2 first blasts hits, iii) the 3 first blasts hits and iv) the 5 first blast hits. In the three latter cases, if the blast hits were classified as belonging to different types, the query sequence was assigned as unclassified. To evaluate the sensitivity and selectivity of the methods, a statistical analysis was performed based on their percentage of recall and precision. Precision is defined as a function of predictions matching the annotated sequences 
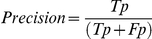
 . It represents the ratio between the number of items correctly predicted as elements of a certain type or class (true positives or Tp), and the total number of items classified as belonging to that type (sum between Tp and items incorrectly classified as members of that class, False positives or Fp).

Recall is the proportion of annotated sequences that match a prediction 
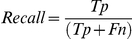
 . It represents the ratio between items correctly assigned as members of a certain type (true positives, Tp) and the total number of members that actually belong to that type, including the ones that were not labeled as members but should have been (false negatives or Fn).

To classify sequences from the Genome data set, a Stand-Alone BLAST was performed in which each sequence was blasted against the Gold Standard data set using BLAST2P search (version 2.2.10,[25]) with the default parameters above described. As before, a similarity matrix was created and a weighted similarity network generated in Cytoscape v2.6.1.

The database was implemented in MySQL version 5.0.51, and the interfaces generated using Django 1.0.2 a Python Web framework (http://www.djangoproject.com/) for Python 2.5.2.

## Results

### Compilation of data sets

The first step in our work was to manually compile a gold standard data set of sequences that we were certain to belong to the HCO superfamily. We further included NORs, as they have been proposed to be evolutionarily related to HCOs [16], [40], [41]. This was initially supported by the observation that NORs and HCO subunit I are assigned to the same predicted SCOP Superfamily [42], and later corroborated by the available structure [18], indicating that most probably NORs and HCOs have a common ancestor.

Using representative sequences for each HCO type or NOR (see methods for details) to query NCBI's protein non-redundant database we identified over 2000 putative oxidases. After filtering and removing redundancy (see methods) we obtained a set of 1216 distinct sequences. This Gold Standard data set is phylogenetically representative, containing 1163 bacterial, 52 archaeal and 1 eukaryotic sequences, which belong to 553 different strains (Table 1).

The two A Types were distinguished on the basis of the amino acid residues making part of the D-proton channel. The B and C Type enzymes were classified according to the absence of the amino acid residues of the D-channel and the existence of the amino acid residues proposed to constitute their K-proton channel [13] (Table 2). NOR sequences were identified by the two motifs, H-X-(Arom)-X-E and H-H-X-(Arom)-(Arom)-X17-E.

**Table 2:** Structural and functional properties of HCOs and NORs.

	Binuclear center				Energy conservation		
Enzyme Type	Heme-Cu	Heme-Fe	Tyr-I	Tyr-II	Charge separation	H^+^ translocation	
						D-channel^a^	K-channel^b^
A	+	-	+	-	+	+	+
B	+	-	+	-	+	-	Altern.
C	+	-	-	+	+	-	Altern.

a The amino acid residues that compose the D-channel are Asp124, Asn199, Asn113, Asn131, Tyr35, Ser134, Ser193 and Glu278 for A1 type enzymes (*P. denitrificans* numbering). In the D-channel of A2 Type enzymes a tyrosine replaces the glutamate residue [13].

b K-channel of A Type enzymes is constituted by Lys354, Thr351, Ser291 and Tyr280 (Tyr-I) residues. In the alternative K-channel of B Type enzymes besides Tyr-I, those residues are replaced by a Thr, a Ser and a Tyr residues. The alternative K-channel from C Type enzymes is constituted by Tyr-II and a Ser and a Tyr residue in the same sequence position of the Thr-351 and SerI-291 residues [13].

### A classification framework for HCOs

We have previously proposed that HCOs should be classified in three distinct types: A Type (subdivided into A1 and A2), B Type and C Type [13]. More recently, and based on a larger number of sequences, this classification was revised by Hemp and Gennis, who proposed five new HCO types (D to H, some of them further divided) comprising 16 sequences from the Archaea domain exclusively [14]. In face of a growing number of HCO and NOR sequences, we revisited the organization of this protein superfamily, using simple sequence similarity and phylogenetic analysis.

As a starting hypothesis, we labeled all the sequences in the gold standard according to our previously proposed classification, based on the presence of key functional residues in the proton conducting channels (Table 1). From the 1216 sequences, only 4 did not contain the amino acid residues proposed to compose the functional channels that characterize each family. These enzymes show the highest similarities with the B Type ones (similarities between 27 and 37%). Importantly, in a global Neighbor-Joining (NJ) tree comprising all amino acid sequences (shown upon request), those sequences cluster with B Type enzymes and thus, we also assigned them as such Type. To avoid large redundancy, eukaryotic A1 heme-copper oxygen reductases were not considered; only a single eukaryotic HCO was found outside the A1 Type. This enzyme, an A2 Type enzyme, is present in the euglyphid amoeba *Paulinella* (*P*.) *chromatophora* that contains two cyanobacterial endosymbionts/plastids, which are close to *Synechococcus sp.* [43]. Within the A Type enzymes, the Cyanobacteria phylum only contains A2 enzymes, and therefore *P. chromatophora* most probably acquired its HCO via its *Synechococcus* endosymbiont (see below).

**Figure 2:**
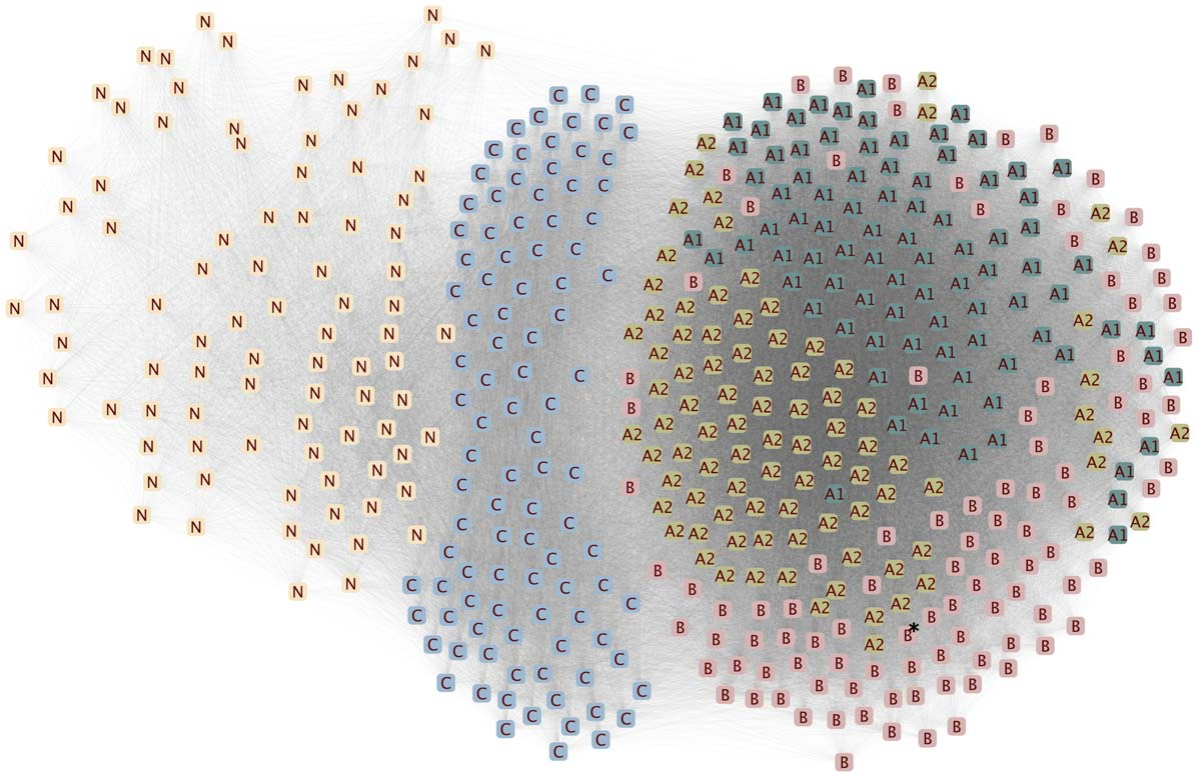
Weighted network representation of similarities for HCOs and NORs. Each square represents an amino acid sequence of subunit I and the lines represent similarities between two sequences. Camel squares (N) – NORs; Blue squares (C) - C Type enzymes; pink squares (B) - B Type enzymes; light green squares (A2) - represent A2 Type enzymes and darker green squares (A1) - A1 Type enzymes; *Thermus thermophilus* B Type enzyme is indicated by an * for reference.

We first built a weighted network based on the pairwise similarity between all the sequences belonging to Gold Standard data (Figure 2). Three interconnected clusters are clearly discernible, comprising i) the NORs (average similarity of 42%), ii) the C Type enzymes (average similarity of 46%) and iii) the A and B Type ones (average similarity of A and B Type enzymes containing cluster of 52%).

The cluster in which the sequences are most tightly connected comprises the A and B Type enzymes, and although in some subgroups a predominance of one specific type can be observed, there are no clear boundaries between the two enzyme types. This reflects a higher similarity between A and B Type enzymes than with the C Type ones (average similarity between A Type and B Type enzymes of 38% and average similarity of A Type with C Type enzymes of 16%).

NORs were proposed to be close to C Type enzymes as observed in an unrooted dendrogram.

This idea was further supported by observing that “subunits II” of C Type HCOs (FixO and FixP) and cNORs contain peripherical cytochrome *c* domains. Cytochrome *c* domains are also present in some subunits II of A type enzymes [13], which show a close structural relation with subunit II (FixO) of C Type enzymes [19]. The same was observed for the cytochrome *c* domains of cNOR and again that of the A Type HCO from *Rhodothermus marinus* [18] ****. We have performed a NJ tree including cytochrome *c* domains of subunits from C Type HCOs (FixO and FixP), subunit II from cNORs (NorC) and subunits II of A Type enzymes that contain a cytochrome *c* domain. We observed no trend that would justify a closer relationship between C Type enzymes and cNOR on the basis of the cytochrome *c* domains [28]. The proximity between the NORs and the C Type enzymes could also be due to artifacts resulting from the higher divergence of these two groups from the A and B Type enzymes [44], [45]. The tight linkage of the NOR cluster to HCOs in the network lends further support to the homology between these two types of proteins [16], [40], as absence of homology would be causing the separation of an independent NOR cluster. Moreover, its closest relation to the C Type enzymes is evidenced by the highest number of interconnections between the two clusters and an average similarity of 21% while the average similarity of NOR to the A and B Type cluster is 9%.

**Figure 3:**
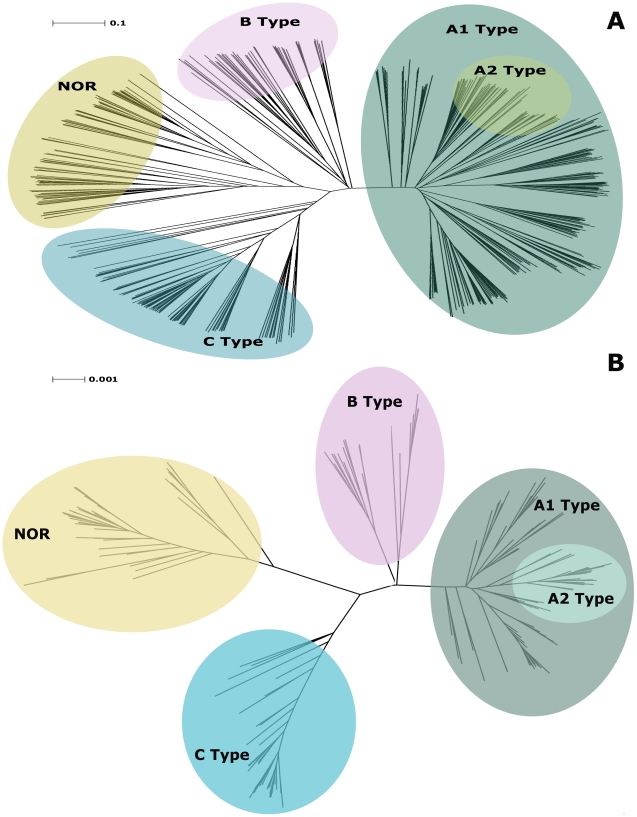
Phylogenetic analysis of subunit I of HCO and NOR enzymes. **A-** Neighbor**-**joining tree representation of subunit I HCO and NOR enzymes. The tree was constructed with the same sequences used for the weighted similarity network. The color code is the same as in Figure 2. **B**- Bayesian tree calculated with selected sequences HCOs from each type and NORs. The sequences were chose in order to have a representative from each taxonomic class.

We further investigated the organization of the family using phylogenetic analysis. We constructed both a Neighbor-Joining (NJ) and a Bayesian tree (Figures 3A and 3B). The NJ tree comprised all sequences used in the weighted similarity network (Figure 3A) whereas the Bayesian tree (Figure 3B), to reduce the computational cost of calculating the tree, included one NOR and one HCO of each type from each taxonomic order. Both trees agree in establishing a clear separation between the clades of the A, B and C Type enzymes as well as of the NOR enzymes. NORs and A Type enzymes are evolutionarily more separated, while the clades for the B and C Type enzymes cluster in between NORs and A Type enzymes (Figure 3A and B). Interestingly, it was observed that archaeal A1 Type enzymes are more closely related to B Type enzymes.

Our analysis fails to support the existence of additional types as proposed by Hemp and Gennis [14]. All the proposed types, two of which constituted by only one member, are included in the previous B Type from Pereira and co-workers [13]. All members of the new types have in common the canonical K-channel features considered to be the fingerprint of B type enzymes. Most importantly, the members of the proposed D to G Types are dispersed in the similarity network and do not form consistent subgroups within the B Type family. It should be stressed that the group of 4 sequences that did not contain the amino acid residues of the proton conducting channel characteristic of B Type enzymes channel did not correspond to any of these new proposed types [14].

In summary, our detailed analysis of a large manually curated set of HCOs and NORs (1216 sequences) revealed that a simple classification in three different types [13] is phylogenetically, functionally and structurally supported (Table 2).

### A classifier for HCOs and NORs

Having established which classification best described the HCO/NOR protein family we now aim to be able to classify large numbers of proteins from completely sequenced genomes, or from any other source. We found that by searching the query sequence against a database composed of the Gold Standard set of sequences using BLASTP, and transferring the annotation of the best-hit, returns the best classification. Note that this only works because the reference database includes the classification of each sequence into a specific type, and this would not work against a generic database as such annotations are not present for many sequences.

**Figure 4:**
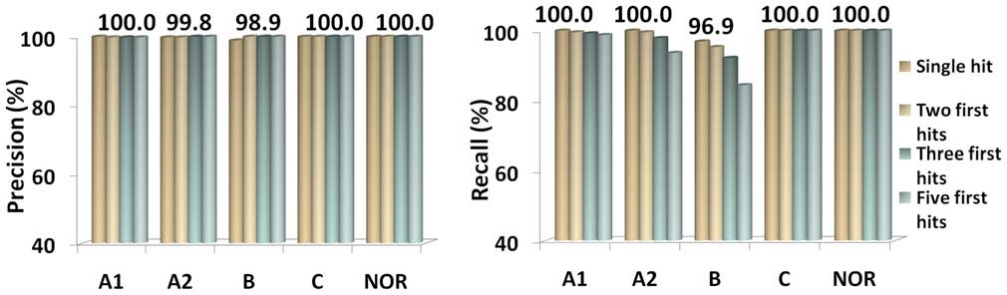
Statistics of HCO automated classification. The left panel shows the Precision () and the right panel the Recall of the first hit, two, three and five first hits for each HCO Type and NOR.

We validated this and alternative classification approaches using a leave-one-out cross verification. This procedure was used with each one of the manually curated sequences. As anticipated from the similarity network (Figure 2), the classification of B Type sequences gave the poorer results. While the results of the other tests did not affect the statistics of the other groups, only using the single first hit, a recall above 96% could be obtained for B Type enzymes. From the classification tests, the first hit was chosen with a global recall and precision of 99.8% (Figure 4).

### A database and web tool

The established method for classification of large number of sequences at high accuracy, was run on completely sequenced bacterial and archaeal genomes, generating what we termed as the Genomes set. We obtained the complete predicted proteomes from the Superfamily database [26] and made use of the fact that all HCOs and NORs belong to the same domain superfamily (Cytochrome *c* oxidase subunit I-like – see methods for details), to reduce the search space, *i.e.* to exclude from our classifier sequences that were not members of this family.

**Figure 5:**
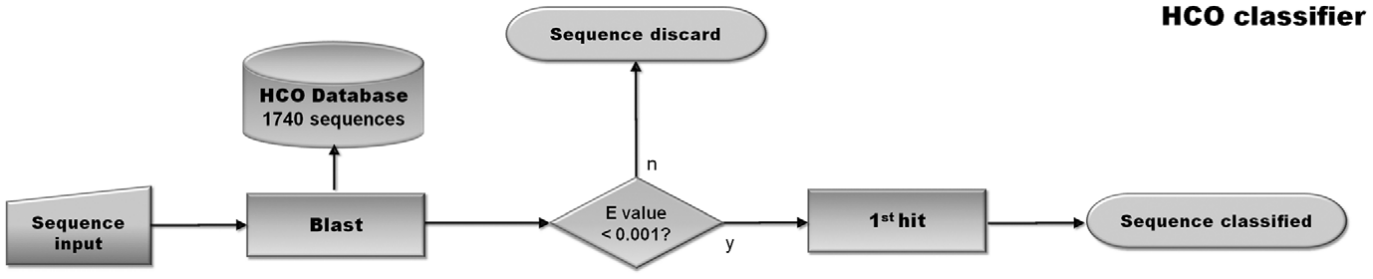
Flowchart of the HCO web-tool classifier. A given sequence is blasted against HCO database. For E value lower than 0.01, the sequence is classified according to the first hit. For E value higher than 0.01, the sequence is discarded and the message: ” the given sequence can not be classified” appear in the screen.

After discarding redundant sequences between the Gold Standard and the Genome data sets, our global dataset, was composed of 1740 sequences from 763 different species/strains. From those, 1327 belong to 552 different strains (376 species) whose genomes have been completely sequenced and annotated at the Superfamily server (Table 3). We make this data available as a public browsable database, the HCO database, at http://www.evocell.org/hco. We further implemented our classification pipeline of HCO and NOR enzymes as a web tool (Figure 5) that can be found on the same site.

**Table 3:** of HCO and NOR sequences or species in HCO database used in this work organized by taxonomic domains and enzyme type.

	Number of HCO/NOR sequences									
	total	Bacteria	Archaea	Eukarya	A1	A2	B	C	NOR	
HCO Database	1740	1685	52	1	936	141	102	352	209	

	Number of species containing HCO/NORs									
	total	strains	Bacteria	Archaea	Eukarya	A1	A2	B	C	NOR
HCO Database	552	773	530	21	1	417	79	63	241	147

Snapshots of the database and its browsing functions are shown in Figure 5. It is possible to select several combinations of criteria according to taxonomy and enzyme type and browse the computed results. Combining different levels in the classification yields different sets of results that can be viewed together. For example, when choosing a taxonomic rank only, the profile of distribution of the different enzyme types can be accessed. This could be used, for example, as a selection criterion of organisms to be cultured in the lab if there is a particular interest in one enzyme Type or to access the taxonomic distribution of the different HCO Types and NORs within the selected taxa.

**Figure 6:**
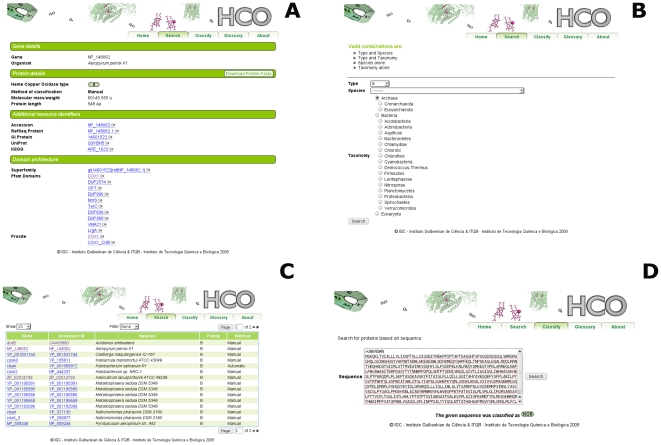
Overview of the core functionality of the HCO web-database. A) A hyperlinked web-page showing details of the protein and annotations of HCO and NOR enzymes. B) Search page which can be explored using the following combinations: Type and Species; Type and Taxonomy; Species alone or Taxonomy alone; C) Web page containing the retrieved result of a query with links to the details of each of the retrieved sequences. D) Classification result of a given fasta sequence.

The HCO web-interface provides an integrated interface, which allows any user to query the database using as input any specific species, taxonomic rank or HCO type/NOR (Figure 6). The HCO web-interface allows: i) a direct classification of the protein according to the different HCO types or NOR; ii) the integration of information from different databases; iii) the analysis of the phylogenetic distribution of the different HCOs/NORs. Thus, this tool provides the scientific community with a specialized and integrated Database in which concise information on the HCO superfamily and NORs can be searched and amino acid sequences classified and retrieved.

## Discussion

Prompted by the high number of presently available sequences of HCO/NORs and completely sequenced genomes we performed a reevaluation of the classification of the HCO superfamily. The present enlarged sequence data set and the available functional and structural data corroborate our previous classification of heme-copper oxygen reductases in three different types [13] and do not show the need for any further division. All our results present no evidence for further types of HCOs or NORs and thus we considered that the classification here employed remains accurate. The results from the similarity network and NJ analysis are complementary being obtained by the different constrains of each method. While in the similarity network we are only comparing the conserved regions between two sequences and thus, looking at the ones that are under a highest pressure to be maintained, in the NJ and Bayesian phylogenetic analysis, the entire sequence length is compared and the specific fingerprints of each enzyme type promote their separation in a tree.

We also conclude that NORs are indeed closely related with C Type enzymes than with the other types of HCOs and exclude the possibility of Long Branch attraction.

Moreover, a tool that allows an automatic classification of HCO and NOR sequences has been constructed and is now available to the scientific community at www.evocell.org/hco. This web-interface can be used in future studies of these enzymes as well as a template to select new targets belonging to phylogenetic distant organisms for biochemical and functional studies.

## Supporting Information

Table S1 Accessions and taxonomic information of sequences deposited at HCO database.
(PDF)

## References

[1] Ducluzeau AL, van Lis R, Duval S, Schoepp-Cothenet B, Russell MJ (2009). Was nitric oxide the first deep electron sink?. Trends Biochem Sci.

[2] Buse G, Soulimane T, Dewor M, Meyer HE, Bluggel M (1999). Evidence for a copper-coordinated histidine-tyrosine cross-link in the active site of cytochrome oxidase.. Protein Sci.

[3] Ferguson-Miller S, Babcock GT (1996). Heme/Copper Terminal Oxidases.. Chem Rev.

[4] Michel H, Behr J, Harrenga A, Kannt A (1998). Cytochrome c oxidase: structure and spectroscopy.. Annu Rev Biophys Biomol Struct.

[5] Gennis RB (1998). Multiple proton-conducting pathways in cytochrome oxidase and a proposed role for the active-site tyrosine.. Biochimica Et Biophysica Acta-Bioenergetics.

[6] Hemp J, Christian C, Barquera B, Gennis RB, Martinez TJ (2005). Helix switching of a key active-site residue in the cytochrome cbb3 oxidases.. Biochemistry.

[7] Iwata S, Ostermeier C, Ludwig B, Michel H (1995). Structure at 2.8 A resolution of cytochrome c oxidase from Paracoccus denitrificans.. Nature.

[8] Verkhovskaya ML, Garcia-Horsman A, Puustinen A, Rigaud JL, Morgan JE (1997). Glutamic acid 286 in subunit I of cytochrome bo3 is involved in proton translocation.. Proc Natl Acad Sci U S A.

[9] Svensson M, Hallen S, Thomas JW, Lemieux LJ, Gennis RB (1995). Oxygen reaction and proton uptake in helix VIII mutants of cytochrome bo3.. Biochemistry.

[10] Tsukihara T, Aoyama H, Yamashita E, Tomizaki T, Yamaguchi H (1996). The whole structure of the 13-subunit oxidized cytochrome c oxidase at 2.8 A.. Science.

[11] Konstantinov AA, Siletsky S, Mitchell D, Kaulen A, Gennis RB (1997). The roles of the two proton input channels in cytochrome c oxidase from Rhodobacter sphaeroides probed by the effects of site-directed mutations on time-resolved electrogenic intraprotein proton transfer.. Proc Natl Acad Sci U S A.

[12] Branden G, Gennis RB, Brzezinski P (2006). Transmembrane proton translocation by cytochrome c oxidase.. Biochim Biophys Acta.

[13] Pereira MM, Santana M, Teixeira M (2001). A novel scenario for the evolution of haem-copper oxygen reductases.. Biochim Biophys Acta.

[14] Hemp J, Gennis RB (2008). Diversity of the heme-copper superfamily in archaea: insights from genomics and structural modeling.. Results Probl Cell Differ.

[15] Hendriks J, Gohlke U, Saraste M (1998). From NO to OO: nitric oxide and dioxygen in bacterial respiration.. J Bioenerg Biomembr.

[16] van der Oost J, de Boer AP, de Gier JW, Zumft WG, Stouthamer AH (1994). The heme-copper oxidase family consists of three distinct types of terminal oxidases and is related to nitric oxide reductase.. FEMS Microbiol Lett.

[17] Zumft WG (2005). Nitric oxide reductases of prokaryotes with emphasis on the respiratory, heme-copper oxidase type.. J Inorg Biochem.

[18] Hino T, Matsumoto Y, Nagano S, Sugimoto H, Fukumori Y (2010). Structural basis of biological N2O generation by bacterial nitric oxide reductase.. Science.

[19] Buschmann S, Warkentin E, Xie H, Langer JD, Ermler U (2010). The Structure of cbb3 Cytochrome Oxidase Provides Insights into Proton Pumping.. Science.

[20] Shapleigh JP, Payne WJ (1985). Nitric Oxide-Dependent Proton Translocation in Various Denitrifiers.. Journal of Bacteriology.

[21] Bell LC, Richardson DJ, Ferguson SJ (1992). Identification of Nitric-Oxide Reductase-Activity in Rhodobacter-Capsulatus - the Electron-Transport Pathway Can Either Use or Bypass Both Cytochrome-C2 and the Cytochrome-Bc1 Complex.. Journal of General Microbiology.

[22] Hendriks JH, Jasaitis A, Saraste M, Verkhovsky MI (2002). Proton and electron pathways in the bacterial nitric oxide reductase.. Biochemistry.

[23] Hao WL, Golding GB (2006). Asymmetrical evolution of cytochrome bd subunits.. Journal of Molecular Evolution.

[24] Vanlerberghe GC, McIntosh L (1997). ALTERNATIVE OXIDASE: From Gene to Function.. Annu Rev Plant Physiol Plant Mol Biol.

[25] Altschul SF, Madden TL, Schaffer AA, Zhang J, Zhang Z (1997). Gapped BLAST and PSI-BLAST: a new generation of protein database search programs.. Nucleic Acids Res.

[26] Gough J, Karplus K, Hughey R, Chothia C (2001). Assignment of homology to genome sequences using a library of hidden Markov models that represent all proteins of known structure.. J Mol Biol.

[27] Finn RD, Tate J, Mistry J, Coggill PC, Sammut SJ (2008). The Pfam protein families database.. Nucleic Acids Research.

[28] Sousa FL (2011). Searching for the common denominator of heme-copper oxygen reductases..

[29] Gasteiger E, Gattiker A, Hoogland C, Ivanyi I, Appel RD (2003). ExPASy: the proteomics server for in-depth protein knowledge and analysis.. Nucleic Acids Research.

[30] Kanehisa M, Goto S, Hattori M, Aoki-Kinoshita KF, Itoh M (2006). From genomics to chemical genomics: new developments in KEGG.. Nucleic Acids Research.

[31] Pruitt KD, Tatusova T, Maglott DR (2007). NCBI reference sequences (RefSeq): a curated non-redundant sequence database of genomes, transcripts and proteins.. Nucleic Acids Research.

[32] Sigrist CJ, Cerutti L, de Castro E, Langendijk-Genevaux PS, Bulliard V PROSITE, a protein domain database for functional characterization and annotation.. Nucleic Acids Res.

[33] Thompson JD, Gibson TJ, Higgins DG (2002). Multiple sequence alignment using ClustalW and ClustalX.. Curr Protoc Bioinformatics Chapter 2: Unit 2.

[34] Nicholas KB, Nicholas, Hugh B (1997). GeneDoc: a tool for editing and annotating multiple sequence alignments..

[35] Altekar G, Dwarkadas S, Huelsenbeck JP, Ronquist F (2004). Parallel Metropolis coupled Markov chain Monte Carlo for Bayesian phylogenetic inference.. Bioinformatics.

[36] Ronquist F, Huelsenbeck JP (2003). MrBayes 3: Bayesian phylogenetic inference under mixed models.. Bioinformatics.

[37] Huson DH, Richter DC, Rausch C, Dezulian T, Franz M (2007). Dendroscope: An interactive viewer for large phylogenetic trees.. BMC Bioinformatics.

[38] Shannon P, Markiel A, Ozier O, Baliga NS, Wang JT (2003). Cytoscape: a software environment for integrated models of biomolecular interaction networks.. Genome Res.

[39] Kamada T, Kawai S (1989). An algorithm for drawing general undirected graphs.. Information Processing Letters.

[40] Saraste M, Castresana J (1994). Cytochrome oxidase evolved by tinkering with denitrification enzymes.. FEBS Lett.

[41] de Vries S, Schroder I (2002). Comparison between the nitric oxide reductase family and its aerobic relatives, the cytochrome oxidases.. Biochemical Society Transactions.

[42] Murzin AG, Brenner SE, Hubbard T, Chothia C (1995). SCOP: a structural classification of proteins database for the investigation of sequences and structures.. J Mol Biol.

[43] Keeling PJ, Archibald JM (2008). Organelle evolution: what's in a name?. Curr Biol.

[44] Gribaldo S, Talla E, Brochier-Armanet C (2009). Evolution of the haem copper oxidases superfamily: a rooting tale.. Trends Biochem Sci.

[45] Brochier-Armanet C, Talla E, Gribaldo S (2009). The multiple evolutionary histories of dioxygen reductases: Implications for the origin and evolution of aerobic respiration.. Mol Biol Evol.

