# Supplementary material for: A Bioinformatics Classifier and Database for Heme-Copper Oxygen Reductases
Source: PLoS One. 2011 Apr 29;6(4):e19117. doi: 10.1371/journal.pone.0019117 (PMC3084760; doi:10.1371/journal.pone.0019117)
Supplement: Table S1 — Accessions and taxonomic information of sequences deposited at HCO database. (PDF) [file pone.0019117.s001.pdf]

Table S1: Accessions and taxonomic information of sequences deposited at HCO database

Accession	species	genus	order	classe	family	phylum	domain
YP_001518912.1	Acaryochloris marina	Acaryochloris				Cyanobacteria	Bacteria
YP_001514849.1	Acaryochloris marina	Acaryochloris				Cyanobacteria	Bacteria
YP_001520798.1	Acaryochloris marina	Acaryochloris				Cyanobacteria	Bacteria
BAB97174.1	Acetobacter pasteurianus	Acetobacter	Acetobacteraceae	Rhodospirillales	Alphaproteobacteria	Proteobacteria	Bacteria
CAC27381.1	Achromobacter cycloclastes	Achromobacter	Alcaligenaceae	Burkholderiales	Betaproteobacteria	Proteobacteria	Bacteria
CAA69980.1	Acidianus ambivalens	Acidianus	Sulfolobaceae	Sulfolobales	Thermoprotei	Crenarchaeota	Archaea
YP_001235284.1	Acidiphilium cryptum	Acidiphilium	Acetobacteraceae	Rhodospirillales	Alphaproteobacteria	Proteobacteria	Bacteria
YP_001234229.1	Acidiphilium cryptum	Acidiphilium	Acetobacteraceae	Rhodospirillales	Alphaproteobacteria	Proteobacteria	Bacteria
YP_002219239.1	Acidithiobacillus ferrooxidans	Acidithiobacillus	Acidithiobacillaceae	Acidithiobacillales	Gammaproteobacteria	Proteobacteria	Bacteria
YP_589517.1	Acidobacteria bacterium Ellin345		Acidobacteriaceae	Acidobacteriales	Acidobacteria (class)	Acidobacteria	Bacteria
YP_592070.1	Acidobacteria bacterium Ellin345		Acidobacteriaceae	Acidobacteriales	Acidobacteria (class)	Acidobacteria	Bacteria
YP_589443.1	Acidobacteria bacterium Ellin345		Acidobacteriaceae	Acidobacteriales	Acidobacteria (class)	Acidobacteria	Bacteria
YP_872249.1	Acidothermus cellulolyticus	Acidothermus	Acidothermaceae	Actinomycetales	Actinobacteria (class)	Actinobacteria	Bacteria
YP_872716.1	Acidothermus cellulolyticus	Acidothermus	Acidothermaceae	Actinomycetales	Actinobacteria (class)	Actinobacteria	Bacteria
YP_972223.1	Acidovorax avenae	Acidovorax	Comamonadaceae	Burkholderiales	Betaproteobacteria	Proteobacteria	Bacteria
YP_971882.1	Acidovorax avenae	Acidovorax	Comamonadaceae	Burkholderiales	Betaproteobacteria	Proteobacteria	Bacteria
YP_970574.1	Acidovorax avenae	Acidovorax	Comamonadaceae	Burkholderiales	Betaproteobacteria	Proteobacteria	Bacteria
YP_969886.1	Acidovorax avenae	Acidovorax	Comamonadaceae	Burkholderiales	Betaproteobacteria	Proteobacteria	Bacteria
YP_987725.1	Acidovorax sp. JS42	Acidovorax	Comamonadaceae	Burkholderiales	Betaproteobacteria	Proteobacteria	Bacteria
YP_986993.1	Acidovorax sp. JS42	Acidovorax	Comamonadaceae	Burkholderiales	Betaproteobacteria	Proteobacteria	Bacteria
YP_985395.1	Acidovorax sp. JS42	Acidovorax	Comamonadaceae	Burkholderiales	Betaproteobacteria	Proteobacteria	Bacteria
YP_984423.1	Acidovorax sp. JS42	Acidovorax	Comamonadaceae	Burkholderiales	Betaproteobacteria	Proteobacteria	Bacteria
YP_987627.1	Acidovorax sp. JS42	Acidovorax	Comamonadaceae	Burkholderiales	Betaproteobacteria	Proteobacteria	Bacteria
YP_002325200.1	Acinetobacter baumannii	Acinetobacter	Moraxellaceae	Pseudomonadales	Gammaproteobacteria	Proteobacteria	Bacteria
YP_002319850.1	Acinetobacter baumannii	Acinetobacter	Moraxellaceae	Pseudomonadales	Gammaproteobacteria	Proteobacteria	Bacteria
YP_001713301.1	Acinetobacter baumannii	Acinetobacter	Moraxellaceae	Pseudomonadales	Gammaproteobacteria	Proteobacteria	Bacteria
YP_001847029.1	Acinetobacter baumannii	Acinetobacter	Moraxellaceae	Pseudomonadales	Gammaproteobacteria	Proteobacteria	Bacteria
YP_001706978.1	Acinetobacter baumannii	Acinetobacter	Moraxellaceae	Pseudomonadales	Gammaproteobacteria	Proteobacteria	Bacteria
YP_001085196.1	Acinetobacter baumannii	Acinetobacter	Moraxellaceae	Pseudomonadales	Gammaproteobacteria	Proteobacteria	Bacteria
YP_047021.1	Acinetobacter sp. ADP1	Acinetobacter	Moraxellaceae	Pseudomonadales	Gammaproteobacteria	Proteobacteria	Bacteria
YP_001344891.1	Actinobacillus succinogenes	Actinobacillus	Pasteurellaceae	Pasteurellales	Gammaproteobacteria	Proteobacteria	Bacteria
YP_857449.1	Aeromonas hydrophila	Aeromonas	Aeromonadaceae	Aeromonadales	Gammaproteobacteria	Proteobacteria	Bacteria
YP_856818.1	Aeromonas hydrophila	Aeromonas	Aeromonadaceae	Aeromonadales	Gammaproteobacteria	Proteobacteria	Bacteria
YP_001142711.1	Aeromonas salmonicida	Aeromonas	Aeromonadaceae	Aeromonadales	Gammaproteobacteria	Proteobacteria	Bacteria
YP_001141804.1	Aeromonas salmonicida	Aeromonas	Aeromonadaceae	Aeromonadales	Gammaproteobacteria	Proteobacteria	Bacteria
NP_147500.2	Aeropyrum pernix	Aeropyrum	Desulfurococcaceae	Desulfurococcales	Thermoprotei	Crenarchaeota	Archaea
NP_148062.1	Aeropyrum pernix	Aeropyrum	Desulfurococcaceae	Desulfurococcales	Thermoprotei	Crenarchaeota	Archaea
NP_353792.2	Agrobacterium tumefaciens	Agrobacterium	Rhizobiaceae	Rhizobiales	Alphaproteobacteria	Proteobacteria	Bacteria
NP_353177.1	Agrobacterium tumefaciens	Agrobacterium	Rhizobiaceae	Rhizobiales	Alphaproteobacteria	Proteobacteria	Bacteria
NP_354541.2	Agrobacterium tumefaciens	Agrobacterium	Rhizobiaceae	Rhizobiales	Alphaproteobacteria	Proteobacteria	Bacteria

CAA86308.1	Agrobacterium tumefaciens	Agrobacterium	Rhizobiaceae	Rhizobiales	Alphaproteobacteria	Proteobacteria	Bacteria
NP_356267.1	Agrobacterium tumefaciens	Agrobacterium	Rhizobiaceae	Rhizobiales	Alphaproteobacteria	Proteobacteria	Bacteria
BAA90782.1	Alcaligenes faecalis	Alcaligenes	Alcaligenaceae	Burkholderiales	Betaproteobacteria	Proteobacteria	Bacteria
YP_693619.1	Alcanivorax borkumensis	Alcanivorax	Alcanivoracaceae	Oceanospirillales	Gammaproteobacteria	Proteobacteria	Bacteria
YP_693756.1	Alcanivorax borkumensis	Alcanivorax	Alcanivoracaceae	Oceanospirillales	Gammaproteobacteria	Proteobacteria	Bacteria

Accession	species	genus	order	classe	family	phylum	domain
YP_693071.1	Alcanivorax borkumensis	Alcanivorax	Alcanivoracaceae	Oceanospirillales	Gammaproteobacteria	Proteobacteria	Bacteria
EDX89879.1	Alcanivorax sp. DG881	Alcanivorax	Alcanivoracaceae	Oceanospirillales	Gammaproteobacteria	Proteobacteria	Bacteria
EDX89574.1	Alcanivorax sp. DG881	Alcanivorax	Alcanivoracaceae	Oceanospirillales	Gammaproteobacteria	Proteobacteria	Bacteria
EDX89384.1	Alcanivorax sp. DG881	Alcanivorax	Alcanivoracaceae	Oceanospirillales	Gammaproteobacteria	Proteobacteria	Bacteria
ZP_01720126.1	Algoriphagus sp. PR1	Algoriphagus	Cyclobacteriaceae	Sphingobacteriales	Sphingobacteria	Bacteroidetes	Bacteria
ZP_01720277.1	Algoriphagus sp. PR1	Algoriphagus	Cyclobacteriaceae	Sphingobacteriales	Sphingobacteria	Bacteroidetes	Bacteria
YP_002262995.1	Aliivibrio salmonicida	Aliivibrio	Vibrionaceae	Vibrionales	Gammaproteobacteria	Proteobacteria	Bacteria
YP_741139.1	Alkalilimnicola ehrlichei	Alkalilimnicola	Ectothiorhodospiraceae	Chromatiales	Gammaproteobacteria	Proteobacteria	Bacteria
YP_742716.1	Alkalilimnicola ehrlichei	Alkalilimnicola	Ectothiorhodospiraceae	Chromatiales	Gammaproteobacteria	Proteobacteria	Bacteria
YP_742950.1	Alkalilimnicola ehrlichei	Alkalilimnicola	Ectothiorhodospiraceae	Chromatiales	Gammaproteobacteria	Proteobacteria	Bacteria
ZP_02186257.1	alpha proteobacterium BAL199				Alphaproteobacteria	Proteobacteria	Bacteria
ZP_01613838.1	Alteromonadales bacterium TW-7			Alteromonadales	Gammaproteobacteria	Proteobacteria	Bacteria
ZP_01614363.1	Alteromonadales bacterium TW-7			Alteromonadales	Gammaproteobacteria	Proteobacteria	Bacteria
YP_002128296.1	Alteromonas macleodii	Alteromonas	Alteromonadaceae	Alteromonadales	Gammaproteobacteria	Proteobacteria	Bacteria
ZP_01108658.1	Alteromonas macleodii	Alteromonas	Alteromonadaceae	Alteromonadales	Gammaproteobacteria	Proteobacteria	Bacteria
YP_002128195.1	Alteromonas macleodii	Alteromonas	Alteromonadaceae	Alteromonadales	Gammaproteobacteria	Proteobacteria	Bacteria
YP_002125779.1	Alteromonas macleodii	Alteromonas	Alteromonadaceae	Alteromonadales	Gammaproteobacteria	Proteobacteria	Bacteria
ZP_01109066.1	Alteromonas macleodii	Alteromonas	Alteromonadaceae	Alteromonadales	Gammaproteobacteria	Proteobacteria	Bacteria
YP_002126350.1	Alteromonas macleodii	Alteromonas	Alteromonadaceae	Alteromonadales	Gammaproteobacteria	Proteobacteria	Bacteria
YP_324077.1	Anabaena variabilis	Anabaena	Nostocaceae	Nostocales		Cyanobacteria	Bacteria
YP_320079.1	Anabaena variabilis	Anabaena	Nostocaceae	Nostocales		Cyanobacteria	Bacteria
YP_324793.1	Anabaena variabilis	Anabaena	Nostocaceae	Nostocales		Cyanobacteria	Bacteria
YP_320968.1	Anabaena variabilis	Anabaena	Nostocaceae	Nostocales		Cyanobacteria	Bacteria
YP_321048.1	Anabaena variabilis	Anabaena	Nostocaceae	Nostocales		Cyanobacteria	Bacteria
ZP_02323500.1	Anaeromyxobacter dehalogenans	Anaeromyxobacter	Myxococcaceae	Myxococcales	Deltaproteobacteria	Proteobacteria	Bacteria
YP_465481.1	Anaeromyxobacter dehalogenans	Anaeromyxobacter	Myxococcaceae	Myxococcales	Deltaproteobacteria	Proteobacteria	Bacteria
YP_464016.1	Anaeromyxobacter dehalogenans	Anaeromyxobacter	Myxococcaceae	Myxococcales	Deltaproteobacteria	Proteobacteria	Bacteria
ZP_02325394.1	Anaeromyxobacter dehalogenans	Anaeromyxobacter	Myxococcaceae	Myxococcales	Deltaproteobacteria	Proteobacteria	Bacteria
YP_464382.1	Anaeromyxobacter dehalogenans	Anaeromyxobacter	Myxococcaceae	Myxococcales	Deltaproteobacteria	Proteobacteria	Bacteria
YP_466923.1	Anaeromyxobacter dehalogenans	Anaeromyxobacter	Myxococcaceae	Myxococcales	Deltaproteobacteria	Proteobacteria	Bacteria
YP_464614.1	Anaeromyxobacter dehalogenans	Anaeromyxobacter	Myxococcaceae	Myxococcales	Deltaproteobacteria	Proteobacteria	Bacteria
YP_466383.1	Anaeromyxobacter dehalogenans	Anaeromyxobacter	Myxococcaceae	Myxococcales	Deltaproteobacteria	Proteobacteria	Bacteria
YP_001378044.1	Anaeromyxobacter sp. Fw109-5	Anaeromyxobacter	Myxococcaceae	Myxococcales	Deltaproteobacteria	Proteobacteria	Bacteria
YP_001378410.1	Anaeromyxobacter sp. Fw109-5	Anaeromyxobacter	Myxococcaceae	Myxococcales	Deltaproteobacteria	Proteobacteria	Bacteria

YP_001381011.1	Anaeromyxobacter sp. Fw109-5	Anaeromyxobacter	Myxococcaceae	Myxococcales	Deltaproteobacteria	Proteobacteria	Bacteria
YP_001379612.1	Anaeromyxobacter sp. Fw109-5	Anaeromyxobacter	Myxococcaceae	Myxococcales	Deltaproteobacteria	Proteobacteria	Bacteria
YP_001379942.1	Anaeromyxobacter sp. Fw109-5	Anaeromyxobacter	Myxococcaceae	Myxococcales	Deltaproteobacteria	Proteobacteria	Bacteria
YP_002133959.1	Anaeromyxobacter sp. K	Anaeromyxobacter	Myxococcaceae	Myxococcales	Deltaproteobacteria	Proteobacteria	Bacteria
ZP_02173620.1	Anaeromyxobacter sp. K	Anaeromyxobacter	Myxococcaceae	Myxococcales	Deltaproteobacteria	Proteobacteria	Bacteria
YP_002133217.1	Anaeromyxobacter sp. K	Anaeromyxobacter	Myxococcaceae	Myxococcales	Deltaproteobacteria	Proteobacteria	Bacteria
YP_002133592.1	Anaeromyxobacter sp. K	Anaeromyxobacter	Myxococcaceae	Myxococcales	Deltaproteobacteria	Proteobacteria	Bacteria
YP_002136117.1	Anaeromyxobacter sp. K	Anaeromyxobacter	Myxococcaceae	Myxococcales	Deltaproteobacteria	Proteobacteria	Bacteria
YP_002134809.1	Anaeromyxobacter sp. K	Anaeromyxobacter	Myxococcaceae	Myxococcales	Deltaproteobacteria	Proteobacteria	Bacteria
YP_002135648.1	Anaeromyxobacter sp. K	Anaeromyxobacter	Myxococcaceae	Myxococcales	Deltaproteobacteria	Proteobacteria	Bacteria
YP_154146.1	Anaplasma marginale	Anaplasma	Anaplasmataceae	Rickettsiales	Alphaproteobacteria	Proteobacteria	Bacteria

Accession	species	genus	order	classe	family	phylum	domain
YP_505633.1	Anaplasma phagocytophilum	Anaplasma	Anaplasmataceae	Rickettsiales	Alphaproteobacteria	Proteobacteria	Bacteria
YP_002314642.1	Anoxybacillus flavithermus	Anoxybacillus	Bacillaceae	Bacillales	Bacilli	Firmicutes	Bacteria
YP_002316213.1	Anoxybacillus flavithermus	Anoxybacillus	Bacillaceae	Bacillales	Bacilli	Firmicutes	Bacteria
YP_002315712.1	Anoxybacillus flavithermus	Anoxybacillus	Bacillaceae	Bacillales	Bacilli	Firmicutes	Bacteria
NP_214504.1	Aquifex aeolicus	Aquifex	Aquificaceae	Aquificales	Aquificae (class)	Aquificae	Bacteria
NP_214506.1	Aquifex aeolicus	Aquifex	Aquificaceae	Aquificales	Aquificae (class)	Aquificae	Bacteria
YP_001490968.1	Arcobacter butzleri	Arcobacter	Campylobacteraceae	Campylobacterales	Epsilonproteobacteria	Proteobacteria	Bacteria
YP_001490279.1	Arcobacter butzleri	Arcobacter	Campylobacteraceae	Campylobacterales	Epsilonproteobacteria	Proteobacteria	Bacteria
YP_159414.1	Aromatoleum aromaticum	Aromatoleum	Rhodocyclaceae	Rhodocyclales	Betaproteobacteria	Proteobacteria	Bacteria
YP_157109.1	Aromatoleum aromaticum	Aromatoleum	Rhodocyclaceae	Rhodocyclales	Betaproteobacteria	Proteobacteria	Bacteria
YP_159601.1	Aromatoleum aromaticum	Aromatoleum	Rhodocyclaceae	Rhodocyclales	Betaproteobacteria	Proteobacteria	Bacteria
YP_159094.1	Aromatoleum aromaticum	Aromatoleum	Rhodocyclaceae	Rhodocyclales	Betaproteobacteria	Proteobacteria	Bacteria
YP_159939.1	Aromatoleum aromaticum	Aromatoleum	Rhodocyclaceae	Rhodocyclales	Betaproteobacteria	Proteobacteria	Bacteria
YP_158648.1	Aromatoleum aromaticum	Aromatoleum	Rhodocyclaceae	Rhodocyclales	Betaproteobacteria	Proteobacteria	Bacteria
YP_157126.1	Aromatoleum aromaticum	Aromatoleum	Rhodocyclaceae	Rhodocyclales	Betaproteobacteria	Proteobacteria	Bacteria
YP_947955.1	Arthrobacter aurescens	Arthrobacter	Micrococcaceae	Actinomycetales	Actinobacteria (class)	Actinobacteria	Bacteria
YP_831696.1	Arthrobacter sp. FB24	Arthrobacter	Micrococcaceae	Actinomycetales	Actinobacteria (class)	Actinobacteria	Bacteria
ZP_03271753.1	Arthrospira maxima	Arthrospira		Oscillatoriales		Cyanobacteria	Bacteria
ZP_03273012.1	Arthrospira maxima	Arthrospira		Oscillatoriales		Cyanobacteria	Bacteria
ZP_01228023.1	Aurantimonas sp. SI85-9A1	Aurantimonas	Aurantimonadaceae	Rhizobiales	Alphaproteobacteria	Proteobacteria	Bacteria
ZP_01228584.1	Aurantimonas sp. SI85-9A1	Aurantimonas	Aurantimonadaceae	Rhizobiales	Alphaproteobacteria	Proteobacteria	Bacteria
ZP_01225850.1	Aurantimonas sp. SI85-9A1	Aurantimonas	Aurantimonadaceae	Rhizobiales	Alphaproteobacteria	Proteobacteria	Bacteria
ZP_01225540.1	Aurantimonas sp. SI85-9A1	Aurantimonas	Aurantimonadaceae	Rhizobiales	Alphaproteobacteria	Proteobacteria	Bacteria
YP_934805.1	Azoarcus sp. BH72	Azoarcus	Rhodocyclaceae	Rhodocyclales	Betaproteobacteria	Proteobacteria	Bacteria
YP_933261.1	Azoarcus sp. BH72	Azoarcus	Rhodocyclaceae	Rhodocyclales	Betaproteobacteria	Proteobacteria	Bacteria
YP_932845.1	Azoarcus sp. BH72	Azoarcus	Rhodocyclaceae	Rhodocyclales	Betaproteobacteria	Proteobacteria	Bacteria
YP_934592.1	Azoarcus sp. BH72	Azoarcus	Rhodocyclaceae	Rhodocyclales	Betaproteobacteria	Proteobacteria	Bacteria

YP_001525875.1	Azorhizobium caulinodans	Azorhizobium	Xanthobacteraceae	Rhizobiales	Alphaproteobacteria	Proteobacteria	Bacteria
YP_001525015.1	Azorhizobium caulinodans	Azorhizobium	Xanthobacteraceae	Rhizobiales	Alphaproteobacteria	Proteobacteria	Bacteria
YP_001527439.1	Azorhizobium caulinodans	Azorhizobium	Xanthobacteraceae	Rhizobiales	Alphaproteobacteria	Proteobacteria	Bacteria
AAC72071.1	Azospirillum brasilense	Azospirillum	Rhodospirillaceae	Rhodospirillales	Alphaproteobacteria	Proteobacteria	Bacteria
ZP_00417290.1	Azotobacter vinelandii	Azotobacter	Pseudomonadaceae	Pseudomonadales	Gammaproteobacteria	Proteobacteria	Bacteria
ZP_00418276.1	Azotobacter vinelandii	Azotobacter	Pseudomonadaceae	Pseudomonadales	Gammaproteobacteria	Proteobacteria	Bacteria
ZP_00418645.1	Azotobacter vinelandii	Azotobacter	Pseudomonadaceae	Pseudomonadales	Gammaproteobacteria	Proteobacteria	Bacteria
YP_001421070.1	Bacillus amyloliquefaciens	Bacillus	Bacillaceae	Bacillales	Bacilli	Firmicutes	Bacteria
YP_001423102.1	Bacillus amyloliquefaciens	Bacillus	Bacillaceae	Bacillales	Bacilli	Firmicutes	Bacteria
YP_020800.1	Bacillus anthracis	Bacillus	Bacillaceae	Bacillales	Bacilli	Firmicutes	Bacteria
NP_846391.1	Bacillus anthracis	Bacillus	Bacillaceae	Bacillales	Bacilli	Firmicutes	Bacteria
YP_030105.1	Bacillus anthracis	Bacillus	Bacillaceae	Bacillales	Bacilli	Firmicutes	Bacteria
YP_017332.1	Bacillus anthracis	Bacillus	Bacillaceae	Bacillales	Bacilli	Firmicutes	Bacteria
NP_843229.1	Bacillus anthracis	Bacillus	Bacillaceae	Bacillales	Bacilli	Firmicutes	Bacteria
YP_026945.1	Bacillus anthracis	Bacillus	Bacillaceae	Bacillales	Bacilli	Firmicutes	Bacteria
YP_002447510.1	Bacillus cereus	Bacillus	Bacillaceae	Bacillales	Bacilli	Firmicutes	Bacteria
YP_002339996.1	Bacillus cereus	Bacillus	Bacillaceae	Bacillales	Bacilli	Firmicutes	Bacteria
YP_085284.1	Bacillus cereus	Bacillus	Bacillaceae	Bacillales	Bacilli	Firmicutes	Bacteria

Accession	species	genus	order	classe	family	phylum	domain
NP_980285.1	Bacillus cereus	Bacillus	Bacillaceae	Bacillales	Bacilli	Firmicutes	Bacteria
YP_002452908.1	Bacillus cereus	Bacillus	Bacillaceae	Bacillales	Bacilli	Firmicutes	Bacteria
ZP_00236857.1	Bacillus cereus	Bacillus	Bacillaceae	Bacillales	Bacilli	Firmicutes	Bacteria
NP_833662.1	Bacillus cereus	Bacillus	Bacillaceae	Bacillales	Bacilli	Firmicutes	Bacteria
YP_002444166.1	Bacillus cereus	Bacillus	Bacillaceae	Bacillales	Bacilli	Firmicutes	Bacteria
YP_002336832.1	Bacillus cereus	Bacillus	Bacillaceae	Bacillales	Bacilli	Firmicutes	Bacteria
YP_082219.1	Bacillus cereus	Bacillus	Bacillaceae	Bacillales	Bacilli	Firmicutes	Bacteria
NP_977096.1	Bacillus cereus	Bacillus	Bacillaceae	Bacillales	Bacilli	Firmicutes	Bacteria
YP_002449730.1	Bacillus cereus	Bacillus	Bacillaceae	Bacillales	Bacilli	Firmicutes	Bacteria
ZP_00239526.1	Bacillus cereus	Bacillus	Bacillaceae	Bacillales	Bacilli	Firmicutes	Bacteria
NP_830510.1	Bacillus cereus	Bacillus	Bacillaceae	Bacillales	Bacilli	Firmicutes	Bacteria
YP_175888.1	Bacillus clausii	Bacillus	Bacillaceae	Bacillales	Bacilli	Firmicutes	Bacteria
YP_175632.1	Bacillus clausii	Bacillus	Bacillaceae	Bacillales	Bacilli	Firmicutes	Bacteria
ZP_01696322.1	Bacillus coagulans	Bacillus	Bacillaceae	Bacillales	Bacilli	Firmicutes	Bacteria
ZP_01697439.1	Bacillus coagulans	Bacillus	Bacillaceae	Bacillales	Bacilli	Firmicutes	Bacteria
ZP_03226521.1	Bacillus coahuilensis	Bacillus	Bacillaceae	Bacillales	Bacilli	Firmicutes	Bacteria
YP_001375874.1	Bacillus cytotoxicus	Bacillus	Bacillaceae	Bacillales	Bacilli	Firmicutes	Bacteria
YP_001373933.1	Bacillus cytotoxicus	Bacillus	Bacillaceae	Bacillales	Bacilli	Firmicutes	Bacteria
AAA22365.1	Bacillus firmus	Bacillus	Bacillaceae	Bacillales	Bacilli	Firmicutes	Bacteria

NP_242931.1	Bacillus halodurans	Bacillus	Bacillaceae	Bacillales	Bacilli	Firmicutes	Bacteria
NP_243480.1	Bacillus halodurans	Bacillus	Bacillaceae	Bacillales	Bacilli	Firmicutes	Bacteria
NP_241605.1	Bacillus halodurans	Bacillus	Bacillaceae	Bacillales	Bacilli	Firmicutes	Bacteria
YP_093545.1	Bacillus licheniformis	Bacillus	Bacillaceae	Bacillales	Bacilli	Firmicutes	Bacteria
YP_081116.1	Bacillus licheniformis	Bacillus	Bacillaceae	Bacillales	Bacilli	Firmicutes	Bacteria
YP_091296.1	Bacillus licheniformis	Bacillus	Bacillaceae	Bacillales	Bacilli	Firmicutes	Bacteria
YP_078884.1	Bacillus licheniformis	Bacillus	Bacillaceae	Bacillales	Bacilli	Firmicutes	Bacteria
YP_091667.1	Bacillus licheniformis	Bacillus	Bacillaceae	Bacillales	Bacilli	Firmicutes	Bacteria
YP_079248.1	Bacillus licheniformis	Bacillus	Bacillaceae	Bacillales	Bacilli	Firmicutes	Bacteria
YP_001486625.1	Bacillus pumilus	Bacillus	Bacillaceae	Bacillales	Bacilli	Firmicutes	Bacteria
YP_001488673.1	Bacillus pumilus	Bacillus	Bacillaceae	Bacillales	Bacilli	Firmicutes	Bacteria
ZP_02171841.1	Bacillus selenitireducens	Bacillus	Bacillaceae	Bacillales	Bacilli	Firmicutes	Bacteria
ZP_01725697.1	Bacillus sp. B14905	Bacillus	Bacillaceae	Bacillales	Bacilli	Firmicutes	Bacteria
ZP_01723962.1	Bacillus sp. B14905	Bacillus	Bacillaceae	Bacillales	Bacilli	Firmicutes	Bacteria
ZP_01721998.1	Bacillus sp. B14905	Bacillus	Bacillaceae	Bacillales	Bacilli	Firmicutes	Bacteria
ZP_01723349.1	Bacillus sp. B14905	Bacillus	Bacillaceae	Bacillales	Bacilli	Firmicutes	Bacteria
NP_389373.1	Bacillus subtilis	Bacillus	Bacillaceae	Bacillales	Bacilli	Firmicutes	Bacteria
CAA38077.1	Bacillus subtilis	Bacillus	Bacillaceae	Bacillales	Bacilli	Firmicutes	Bacteria
NP_391695.1	Bacillus subtilis	Bacillus	Bacillaceae	Bacillales	Bacilli	Firmicutes	Bacteria
ZP_00741993.1	Bacillus thuringiensis	Bacillus	Bacillaceae	Bacillales	Bacilli	Firmicutes	Bacteria
YP_038005.1	Bacillus thuringiensis	Bacillus	Bacillaceae	Bacillales	Bacilli	Firmicutes	Bacteria
YP_896311.1	Bacillus thuringiensis	Bacillus	Bacillaceae	Bacillales	Bacilli	Firmicutes	Bacteria
ZP_00742474.1	Bacillus thuringiensis	Bacillus	Bacillaceae	Bacillales	Bacilli	Firmicutes	Bacteria
YP_034958.1	Bacillus thuringiensis	Bacillus	Bacillaceae	Bacillales	Bacilli	Firmicutes	Bacteria
YP_893531.1	Bacillus thuringiensis	Bacillus	Bacillaceae	Bacillales	Bacilli	Firmicutes	Bacteria
YP_001646566.1	Bacillus weihenstephanensis	Bacillus	Bacillaceae	Bacillales	Bacilli	Firmicutes	Bacteria

Accession	species	genus	order	classe	family	phylum	domain
YP_001643510.1	Bacillus weihenstephanensis	Bacillus	Bacillaceae	Bacillales	Bacilli	Firmicutes	Bacteria
ZP_02964949.1	bacterium Ellin514		Verrucomicrobia subdivis	Verrucomicrobiales	Verrucomicrobiae	Verrucomicrobia	Bacteria
ZP_02965874.1	bacterium Ellin514		Verrucomicrobia subdivis	Verrucomicrobiales	Verrucomicrobiae	Verrucomicrobia	Bacteria
YP_989523.1	Bartonella bacilliformis	Bartonella	Bartonellaceae	Rhizobiales	Alphaproteobacteria	Proteobacteria	Bacteria
YP_032982.1	Bartonella henselae	Bartonella	Bartonellaceae	Rhizobiales	Alphaproteobacteria	Proteobacteria	Bacteria
YP_031835.1	Bartonella quintana	Bartonella	Bartonellaceae	Rhizobiales	Alphaproteobacteria	Proteobacteria	Bacteria
YP_001608617.1	Bartonella tribocorum	Bartonella	Bartonellaceae	Rhizobiales	Alphaproteobacteria	Proteobacteria	Bacteria
NP_967290.1	Bdellovibrio bacteriovorus	Bdellovibrio	Bdellovibrionaceae	Bdellovibrionales	Deltaproteobacteria	Proteobacteria	Bacteria
NP_969411.1	Bdellovibrio bacteriovorus	Bdellovibrio	Bdellovibrionaceae	Bdellovibrionales	Deltaproteobacteria	Proteobacteria	Bacteria
NP_969401.1	Bdellovibrio bacteriovorus	Bdellovibrio	Bdellovibrionaceae	Bdellovibrionales	Deltaproteobacteria	Proteobacteria	Bacteria
ZP_01999494.1	Beggiatoa sp. PS	Beggiatoa	Thiotrichaceae	Thiotrichales	Gammaproteobacteria	Proteobacteria	Bacteria

ZP_02000496.1	Beggiatoa sp. PS	Beggiatoa	Thiotrichaceae	Thiotrichales	Gammaproteobacteria	Proteobacteria	Bacteria
ZP_02001268.1	Beggiatoa sp. PS	Beggiatoa	Thiotrichaceae	Thiotrichales	Gammaproteobacteria	Proteobacteria	Bacteria
YP_001831868.1	Beijerinckia indica	Beijerinckia	Beijerinckiaceae	Rhizobiales	Alphaproteobacteria	Proteobacteria	Bacteria
ZP_01306902.1	Bermanella marisrubri	Bermanella	Oceanospirillaceae	Oceanospirillales	Gammaproteobacteria	Proteobacteria	Bacteria
ZP_01305640.1	Bermanella marisrubri	Bermanella	Oceanospirillaceae	Oceanospirillales	Gammaproteobacteria	Proteobacteria	Bacteria
EDZ65002.1	beta proteobacterium KB13				Betaproteobacteria	Proteobacteria	Bacteria
ZP_01093641.1	Blastopirellula marina	Blastopirellula	Planctomycetaceae	Planctomycetales	Planctomycetacia	Planctomycetes	Bacteria
ZP_01091121.1	Blastopirellula marina	Blastopirellula	Planctomycetaceae	Planctomycetales	Planctomycetacia	Planctomycetes	Bacteria
ZP_01094316.1	Blastopirellula marina	Blastopirellula	Planctomycetaceae	Planctomycetales	Planctomycetacia	Planctomycetes	Bacteria
YP_785316.1	Bordetella avium	Bordetella	Alcaligenaceae	Burkholderiales	Betaproteobacteria	Proteobacteria	Bacteria
NP_891363.1	Bordetella bronchiseptica	Bordetella	Alcaligenaceae	Burkholderiales	Betaproteobacteria	Proteobacteria	Bacteria
NP_891206.1	Bordetella bronchiseptica	Bordetella	Alcaligenaceae	Burkholderiales	Betaproteobacteria	Proteobacteria	Bacteria
NP_887830.1	Bordetella bronchiseptica	Bordetella	Alcaligenaceae	Burkholderiales	Betaproteobacteria	Proteobacteria	Bacteria
NP_887855.1	Bordetella bronchiseptica	Bordetella	Alcaligenaceae	Burkholderiales	Betaproteobacteria	Proteobacteria	Bacteria
NP_889864.1	Bordetella bronchiseptica	Bordetella	Alcaligenaceae	Burkholderiales	Betaproteobacteria	Proteobacteria	Bacteria
NP_886371.1	Bordetella parapertussis	Bordetella	Alcaligenaceae	Burkholderiales	Betaproteobacteria	Proteobacteria	Bacteria
NP_886335.1	Bordetella parapertussis	Bordetella	Alcaligenaceae	Burkholderiales	Betaproteobacteria	Proteobacteria	Bacteria
NP_883388.1	Bordetella parapertussis	Bordetella	Alcaligenaceae	Burkholderiales	Betaproteobacteria	Proteobacteria	Bacteria
NP_883412.1	Bordetella parapertussis	Bordetella	Alcaligenaceae	Burkholderiales	Betaproteobacteria	Proteobacteria	Bacteria
NP_884049.1	Bordetella parapertussis	Bordetella	Alcaligenaceae	Burkholderiales	Betaproteobacteria	Proteobacteria	Bacteria
NP_882246.1	Bordetella pertussis	Bordetella	Alcaligenaceae	Burkholderiales	Betaproteobacteria	Proteobacteria	Bacteria
NP_881514.1	Bordetella pertussis	Bordetella	Alcaligenaceae	Burkholderiales	Betaproteobacteria	Proteobacteria	Bacteria
NP_881338.1	Bordetella pertussis	Bordetella	Alcaligenaceae	Burkholderiales	Betaproteobacteria	Proteobacteria	Bacteria
YP_001628771.1	Bordetella petrii	Bordetella	Alcaligenaceae	Burkholderiales	Betaproteobacteria	Proteobacteria	Bacteria
YP_001630971.1	Bordetella petrii	Bordetella	Alcaligenaceae	Burkholderiales	Betaproteobacteria	Proteobacteria	Bacteria
YP_001631310.1	Bordetella petrii	Bordetella	Alcaligenaceae	Burkholderiales	Betaproteobacteria	Proteobacteria	Bacteria
YP_001632007.1	Bordetella petrii	Bordetella	Alcaligenaceae	Burkholderiales	Betaproteobacteria	Proteobacteria	Bacteria
YP_001631825.1	Bordetella petrii	Bordetella	Alcaligenaceae	Burkholderiales	Betaproteobacteria	Proteobacteria	Bacteria
YP_001631855.1	Bordetella petrii	Bordetella	Alcaligenaceae	Burkholderiales	Betaproteobacteria	Proteobacteria	Bacteria
YP_001632171.1	Bordetella petrii	Bordetella	Alcaligenaceae	Burkholderiales	Betaproteobacteria	Proteobacteria	Bacteria
YP_001632665.1	Bordetella petrii	Bordetella	Alcaligenaceae	Burkholderiales	Betaproteobacteria	Proteobacteria	Bacteria
NP_767811.1	Bradyrhizobium japonicum	Bradyrhizobium	Bradyrhizobiaceae	Rhizobiales	Alphaproteobacteria	Proteobacteria	Bacteria
NP_770424.1	Bradyrhizobium japonicum	Bradyrhizobium	Bradyrhizobiaceae	Rhizobiales	Alphaproteobacteria	Proteobacteria	Bacteria
NP_766790.1	Bradyrhizobium japonicum	Bradyrhizobium	Bradyrhizobiaceae	Rhizobiales	Alphaproteobacteria	Proteobacteria	Bacteria

**Accession**	**species**	**genus**	**order**	**classe**	**family**	**phylum**	**domain**
NP_769355.1	Bradyrhizobium japonicum	Bradyrhizobium	Bradyrhizobiaceae	Rhizobiales	Alphaproteobacteria	Proteobacteria	Bacteria
NP_771120.1	Bradyrhizobium japonicum	Bradyrhizobium	Bradyrhizobiaceae	Rhizobiales	Alphaproteobacteria	Proteobacteria	Bacteria
NP_769403.1	Bradyrhizobium japonicum	Bradyrhizobium	Bradyrhizobiaceae	Rhizobiales	Alphaproteobacteria	Proteobacteria	Bacteria

NP_769855.1	Bradyrhizobium japonicum	Bradyrhizobium	Bradyrhizobiaceae	Rhizobiales	Alphaproteobacteria	Proteobacteria	Bacteria
YP_001236997.1	Bradyrhizobium sp. BTAi1	Bradyrhizobium	Bradyrhizobiaceae	Rhizobiales	Alphaproteobacteria	Proteobacteria	Bacteria
YP_001238127.1	Bradyrhizobium sp. BTAi1	Bradyrhizobium	Bradyrhizobiaceae	Rhizobiales	Alphaproteobacteria	Proteobacteria	Bacteria
YP_001239191.1	Bradyrhizobium sp. BTAi1	Bradyrhizobium	Bradyrhizobiaceae	Rhizobiales	Alphaproteobacteria	Proteobacteria	Bacteria
YP_001238831.1	Bradyrhizobium sp. BTAi1	Bradyrhizobium	Bradyrhizobiaceae	Rhizobiales	Alphaproteobacteria	Proteobacteria	Bacteria
YP_001238182.1	Bradyrhizobium sp. BTAi1	Bradyrhizobium	Bradyrhizobiaceae	Rhizobiales	Alphaproteobacteria	Proteobacteria	Bacteria
YP_001208524.1	Bradyrhizobium sp. ORS278	Bradyrhizobium	Bradyrhizobiaceae	Rhizobiales	Alphaproteobacteria	Proteobacteria	Bacteria
YP_001203834.1	Bradyrhizobium sp. ORS278	Bradyrhizobium	Bradyrhizobiaceae	Rhizobiales	Alphaproteobacteria	Proteobacteria	Bacteria
YP_001206809.1	Bradyrhizobium sp. ORS278	Bradyrhizobium	Bradyrhizobiaceae	Rhizobiales	Alphaproteobacteria	Proteobacteria	Bacteria
YP_001204332.1	Bradyrhizobium sp. ORS278	Bradyrhizobium	Bradyrhizobiaceae	Rhizobiales	Alphaproteobacteria	Proteobacteria	Bacteria
YP_001204500.1	Bradyrhizobium sp. ORS278	Bradyrhizobium	Bradyrhizobiaceae	Rhizobiales	Alphaproteobacteria	Proteobacteria	Bacteria
YP_001203888.1	Bradyrhizobium sp. ORS278	Bradyrhizobium	Bradyrhizobiaceae	Rhizobiales	Alphaproteobacteria	Proteobacteria	Bacteria
ZP_00378590.1	Brevibacterium linens	Brevibacterium	Brevibacteriaceae	Actinomycetales	Actinobacteria (class)	Actinobacteria	Bacteria
EDX81437.1	Brevundimonas sp. BAL3	Brevundimonas	Caulobacteraceae	Caulobacterales	Alphaproteobacteria	Proteobacteria	Bacteria
EDX80508.1	Brevundimonas sp. BAL3	Brevundimonas	Caulobacteraceae	Caulobacterales	Alphaproteobacteria	Proteobacteria	Bacteria
EDX79523.1	Brevundimonas sp. BAL3	Brevundimonas	Caulobacteraceae	Caulobacterales	Alphaproteobacteria	Proteobacteria	Bacteria
YP_001934469.1	Brucella abortus	Brucella	Brucellaceae	Rhizobiales	Alphaproteobacteria	Proteobacteria	Bacteria
YP_221246.1	Brucella abortus	Brucella	Brucellaceae	Rhizobiales	Alphaproteobacteria	Proteobacteria	Bacteria
YP_413953.1	Brucella abortus	Brucella	Brucellaceae	Rhizobiales	Alphaproteobacteria	Proteobacteria	Bacteria
YP_001934376.1	Brucella abortus	Brucella	Brucellaceae	Rhizobiales	Alphaproteobacteria	Proteobacteria	Bacteria
YP_221148.1	Brucella abortus	Brucella	Brucellaceae	Rhizobiales	Alphaproteobacteria	Proteobacteria	Bacteria
YP_413854.1	Brucella abortus	Brucella	Brucellaceae	Rhizobiales	Alphaproteobacteria	Proteobacteria	Bacteria
YP_419097.1	Brucella abortus	Brucella	Brucellaceae	Rhizobiales	Alphaproteobacteria	Proteobacteria	Bacteria
YP_223677.1	Brucella abortus	Brucella	Brucellaceae	Rhizobiales	Alphaproteobacteria	Proteobacteria	Bacteria
YP_001932818.1	Brucella abortus	Brucella	Brucellaceae	Rhizobiales	Alphaproteobacteria	Proteobacteria	Bacteria
YP_001592327.1	Brucella canis	Brucella	Brucellaceae	Rhizobiales	Alphaproteobacteria	Proteobacteria	Bacteria
YP_001591920.1	Brucella canis	Brucella	Brucellaceae	Rhizobiales	Alphaproteobacteria	Proteobacteria	Bacteria
YP_001592230.1	Brucella canis	Brucella	Brucellaceae	Rhizobiales	Alphaproteobacteria	Proteobacteria	Bacteria
YP_001594211.1	Brucella canis	Brucella	Brucellaceae	Rhizobiales	Alphaproteobacteria	Proteobacteria	Bacteria
NP_540382.1	Brucella melitensis	Brucella	Brucellaceae	Rhizobiales	Alphaproteobacteria	Proteobacteria	Bacteria
NP_540817.1	Brucella melitensis	Brucella	Brucellaceae	Rhizobiales	Alphaproteobacteria	Proteobacteria	Bacteria
NP_540481.1	Brucella melitensis	Brucella	Brucellaceae	Rhizobiales	Alphaproteobacteria	Proteobacteria	Bacteria
NP_541976.1	Brucella melitensis	Brucella	Brucellaceae	Rhizobiales	Alphaproteobacteria	Proteobacteria	Bacteria
YP_001258486.1	Brucella ovis	Brucella	Brucellaceae	Rhizobiales	Alphaproteobacteria	Proteobacteria	Bacteria
YP_001258090.1	Brucella ovis	Brucella	Brucellaceae	Rhizobiales	Alphaproteobacteria	Proteobacteria	Bacteria
YP_001258397.1	Brucella ovis	Brucella	Brucellaceae	Rhizobiales	Alphaproteobacteria	Proteobacteria	Bacteria
NP_697496.1	Brucella suis	Brucella	Brucellaceae	Rhizobiales	Alphaproteobacteria	Proteobacteria	Bacteria
YP_001627153.1	Brucella suis	Brucella	Brucellaceae	Rhizobiales	Alphaproteobacteria	Proteobacteria	Bacteria
NP_697085.1	Brucella suis	Brucella	Brucellaceae	Rhizobiales	Alphaproteobacteria	Proteobacteria	Bacteria
YP_001626723.1	Brucella suis	Brucella	Brucellaceae	Rhizobiales	Alphaproteobacteria	Proteobacteria	Bacteria
YP_001627054.1	Brucella suis	Brucella	Brucellaceae	Rhizobiales	Alphaproteobacteria	Proteobacteria	Bacteria
NP_697395.1	Brucella suis	Brucella	Brucellaceae	Rhizobiales	Alphaproteobacteria	Proteobacteria	Bacteria

Accession	species	genus	order	classe	family	phylum	domain
YP_001622088.1	Brucella suis	Brucella	Brucellaceae	Rhizobiales	Alphaproteobacteria	Proteobacteria	Bacteria
NP_699446.1	Brucella suis	Brucella	Brucellaceae	Rhizobiales	Alphaproteobacteria	Proteobacteria	Bacteria
YP_002468760.1	Buchnera aphidicola	Buchnera	Enterobacteriaceae	Enterobacteriales	Gammaproteobacteria	Proteobacteria	Bacteria
NP_240282.1	Buchnera aphidicola	Buchnera	Enterobacteriaceae	Enterobacteriales	Gammaproteobacteria	Proteobacteria	Bacteria
YP_002468207.1	Buchnera aphidicola	Buchnera	Enterobacteriaceae	Enterobacteriales	Gammaproteobacteria	Proteobacteria	Bacteria
NP_660787.1	Buchnera aphidicola	Buchnera	Enterobacteriaceae	Enterobacteriales	Gammaproteobacteria	Proteobacteria	Bacteria
YP_802843.1	Buchnera aphidicola	Buchnera	Enterobacteriaceae	Enterobacteriales	Gammaproteobacteria	Proteobacteria	Bacteria
NP_778021.1	Buchnera aphidicola	Buchnera	Enterobacteriaceae	Enterobacteriales	Gammaproteobacteria	Proteobacteria	Bacteria
YP_001809456.1	Burkholderia ambifaria	Burkholderia	Burkholderiaceae	Burkholderiales	Betaproteobacteria	Proteobacteria	Bacteria
YP_774791.1	Burkholderia ambifaria	Burkholderia	Burkholderiaceae	Burkholderiales	Betaproteobacteria	Proteobacteria	Bacteria
YP_001815927.1	Burkholderia ambifaria	Burkholderia	Burkholderiaceae	Burkholderiales	Betaproteobacteria	Proteobacteria	Bacteria
YP_778088.1	Burkholderia ambifaria	Burkholderia	Burkholderiaceae	Burkholderiales	Betaproteobacteria	Proteobacteria	Bacteria
YP_001808673.1	Burkholderia ambifaria	Burkholderia	Burkholderiaceae	Burkholderiales	Betaproteobacteria	Proteobacteria	Bacteria
YP_773996.1	Burkholderia ambifaria	Burkholderia	Burkholderiaceae	Burkholderiales	Betaproteobacteria	Proteobacteria	Bacteria
YP_001816273.1	Burkholderia ambifaria	Burkholderia	Burkholderiaceae	Burkholderiales	Betaproteobacteria	Proteobacteria	Bacteria
YP_778451.1	Burkholderia ambifaria	Burkholderia	Burkholderiaceae	Burkholderiales	Betaproteobacteria	Proteobacteria	Bacteria
YP_001811293.1	Burkholderia ambifaria	Burkholderia	Burkholderiaceae	Burkholderiales	Betaproteobacteria	Proteobacteria	Bacteria
YP_002229907.1	Burkholderia cenocepacia	Burkholderia	Burkholderiaceae	Burkholderiales	Betaproteobacteria	Proteobacteria	Bacteria
YP_836489.1	Burkholderia cenocepacia	Burkholderia	Burkholderiaceae	Burkholderiales	Betaproteobacteria	Proteobacteria	Bacteria
YP_001766138.1	Burkholderia cenocepacia	Burkholderia	Burkholderiaceae	Burkholderiales	Betaproteobacteria	Proteobacteria	Bacteria
YP_622106.1	Burkholderia cenocepacia	Burkholderia	Burkholderiaceae	Burkholderiales	Betaproteobacteria	Proteobacteria	Bacteria
YP_835714.1	Burkholderia cenocepacia	Burkholderia	Burkholderiaceae	Burkholderiales	Betaproteobacteria	Proteobacteria	Bacteria
YP_625845.1	Burkholderia cenocepacia	Burkholderia	Burkholderiaceae	Burkholderiales	Betaproteobacteria	Proteobacteria	Bacteria
YP_001765372.1	Burkholderia cenocepacia	Burkholderia	Burkholderiaceae	Burkholderiales	Betaproteobacteria	Proteobacteria	Bacteria
YP_002231271.1	Burkholderia cenocepacia	Burkholderia	Burkholderiaceae	Burkholderiales	Betaproteobacteria	Proteobacteria	Bacteria
YP_840161.1	Burkholderia cenocepacia	Burkholderia	Burkholderiaceae	Burkholderiales	Betaproteobacteria	Proteobacteria	Bacteria
YP_621172.1	Burkholderia cenocepacia	Burkholderia	Burkholderiaceae	Burkholderiales	Betaproteobacteria	Proteobacteria	Bacteria
YP_002153874.1	Burkholderia cenocepacia	Burkholderia	Burkholderiaceae	Burkholderiales	Betaproteobacteria	Proteobacteria	Bacteria
YP_001773760.1	Burkholderia cenocepacia	Burkholderia	Burkholderiaceae	Burkholderiales	Betaproteobacteria	Proteobacteria	Bacteria
YP_002097350.1	Burkholderia dolosa	Burkholderia	Burkholderiaceae	Burkholderiales	Betaproteobacteria	Proteobacteria	Bacteria
YP_002098023.1	Burkholderia dolosa	Burkholderia	Burkholderiaceae	Burkholderiales	Betaproteobacteria	Proteobacteria	Bacteria
YP_002101429.1	Burkholderia dolosa	Burkholderia	Burkholderiaceae	Burkholderiales	Betaproteobacteria	Proteobacteria	Bacteria
ZP_02882701.1	Burkholderia graminis	Burkholderia	Burkholderiaceae	Burkholderiales	Betaproteobacteria	Proteobacteria	Bacteria
ZP_02884057.1	Burkholderia graminis	Burkholderia	Burkholderiaceae	Burkholderiales	Betaproteobacteria	Proteobacteria	Bacteria
ZP_02883997.1	Burkholderia graminis	Burkholderia	Burkholderiaceae	Burkholderiales	Betaproteobacteria	Proteobacteria	Bacteria
ZP_02882963.1	Burkholderia graminis	Burkholderia	Burkholderiaceae	Burkholderiales	Betaproteobacteria	Proteobacteria	Bacteria
ZP_02887806.1	Burkholderia graminis	Burkholderia	Burkholderiaceae	Burkholderiales	Betaproteobacteria	Proteobacteria	Bacteria
YP_001082371.1	Burkholderia mallei	Burkholderia	Burkholderiaceae	Burkholderiales	Betaproteobacteria	Proteobacteria	Bacteria

YP_001027406.1	Burkholderia mallei	Burkholderia	Burkholderiaceae	Burkholderiales	Betaproteobacteria	Proteobacteria	Bacteria
YP_104673.1	Burkholderia mallei	Burkholderia	Burkholderiaceae	Burkholderiales	Betaproteobacteria	Proteobacteria	Bacteria
YP_991520.1	Burkholderia mallei	Burkholderia	Burkholderiaceae	Burkholderiales	Betaproteobacteria	Proteobacteria	Bacteria
YP_105022.1	Burkholderia mallei	Burkholderia	Burkholderiaceae	Burkholderiales	Betaproteobacteria	Proteobacteria	Bacteria
YP_001025367.1	Burkholderia mallei	Burkholderia	Burkholderiaceae	Burkholderiales	Betaproteobacteria	Proteobacteria	Bacteria
YP_990966.1	Burkholderia mallei	Burkholderia	Burkholderiaceae	Burkholderiales	Betaproteobacteria	Proteobacteria	Bacteria
YP_001077431.1	Burkholderia mallei	Burkholderia	Burkholderiaceae	Burkholderiales	Betaproteobacteria	Proteobacteria	Bacteria

Accession	species	genus	order	classe	family	phylum	domain
YP_993718.1	Burkholderia mallei	Burkholderia	Burkholderiaceae	Burkholderiales	Betaproteobacteria	Proteobacteria	Bacteria
YP_001081265.1	Burkholderia mallei	Burkholderia	Burkholderiaceae	Burkholderiales	Betaproteobacteria	Proteobacteria	Bacteria
YP_001028824.1	Burkholderia mallei	Burkholderia	Burkholderiaceae	Burkholderiales	Betaproteobacteria	Proteobacteria	Bacteria
YP_993685.1	Burkholderia mallei	Burkholderia	Burkholderiaceae	Burkholderiales	Betaproteobacteria	Proteobacteria	Bacteria
YP_001081235.1	Burkholderia mallei	Burkholderia	Burkholderiaceae	Burkholderiales	Betaproteobacteria	Proteobacteria	Bacteria
YP_001028854.1	Burkholderia mallei	Burkholderia	Burkholderiaceae	Burkholderiales	Betaproteobacteria	Proteobacteria	Bacteria
YP_102415.1	Burkholderia mallei	Burkholderia	Burkholderiaceae	Burkholderiales	Betaproteobacteria	Proteobacteria	Bacteria
YP_001024473.1	Burkholderia mallei	Burkholderia	Burkholderiaceae	Burkholderiales	Betaproteobacteria	Proteobacteria	Bacteria
YP_105512.1	Burkholderia mallei	Burkholderia	Burkholderiaceae	Burkholderiales	Betaproteobacteria	Proteobacteria	Bacteria
YP_990155.1	Burkholderia mallei	Burkholderia	Burkholderiaceae	Burkholderiales	Betaproteobacteria	Proteobacteria	Bacteria
YP_001078795.1	Burkholderia mallei	Burkholderia	Burkholderiaceae	Burkholderiales	Betaproteobacteria	Proteobacteria	Bacteria
ZP_00440513.1	Burkholderia mallei	Burkholderia	Burkholderiaceae	Burkholderiales	Betaproteobacteria	Proteobacteria	Bacteria
YP_001947222.1	Burkholderia multivorans	Burkholderia	Burkholderiaceae	Burkholderiales	Betaproteobacteria	Proteobacteria	Bacteria
YP_001578649.1	Burkholderia multivorans	Burkholderia	Burkholderiaceae	Burkholderiales	Betaproteobacteria	Proteobacteria	Bacteria
YP_001946490.1	Burkholderia multivorans	Burkholderia	Burkholderiaceae	Burkholderiales	Betaproteobacteria	Proteobacteria	Bacteria
YP_001579395.1	Burkholderia multivorans	Burkholderia	Burkholderiaceae	Burkholderiales	Betaproteobacteria	Proteobacteria	Bacteria
YP_001941565.1	Burkholderia multivorans	Burkholderia	Burkholderiaceae	Burkholderiales	Betaproteobacteria	Proteobacteria	Bacteria
YP_001585717.1	Burkholderia multivorans	Burkholderia	Burkholderiaceae	Burkholderiales	Betaproteobacteria	Proteobacteria	Bacteria
YP_001585463.1	Burkholderia multivorans	Burkholderia	Burkholderiaceae	Burkholderiales	Betaproteobacteria	Proteobacteria	Bacteria
YP_001941788.1	Burkholderia multivorans	Burkholderia	Burkholderiaceae	Burkholderiales	Betaproteobacteria	Proteobacteria	Bacteria
ZP_02354324.1	Burkholderia oklahomensis	Burkholderia	Burkholderiaceae	Burkholderiales	Betaproteobacteria	Proteobacteria	Bacteria
ZP_02363473.1	Burkholderia oklahomensis	Burkholderia	Burkholderiaceae	Burkholderiales	Betaproteobacteria	Proteobacteria	Bacteria
ZP_02356343.1	Burkholderia oklahomensis	Burkholderia	Burkholderiaceae	Burkholderiales	Betaproteobacteria	Proteobacteria	Bacteria
YP_001856516.1	Burkholderia phymatum	Burkholderia	Burkholderiaceae	Burkholderiales	Betaproteobacteria	Proteobacteria	Bacteria
YP_001861937.1	Burkholderia phymatum	Burkholderia	Burkholderiaceae	Burkholderiales	Betaproteobacteria	Proteobacteria	Bacteria
YP_001859829.1	Burkholderia phymatum	Burkholderia	Burkholderiaceae	Burkholderiales	Betaproteobacteria	Proteobacteria	Bacteria
YP_001860138.1	Burkholderia phymatum	Burkholderia	Burkholderiaceae	Burkholderiales	Betaproteobacteria	Proteobacteria	Bacteria
YP_001860947.1	Burkholderia phymatum	Burkholderia	Burkholderiaceae	Burkholderiales	Betaproteobacteria	Proteobacteria	Bacteria
YP_001856586.1	Burkholderia phymatum	Burkholderia	Burkholderiaceae	Burkholderiales	Betaproteobacteria	Proteobacteria	Bacteria
YP_001861613.1	Burkholderia phymatum	Burkholderia	Burkholderiaceae	Burkholderiales	Betaproteobacteria	Proteobacteria	Bacteria

Accession	species	genus	order	classe	family	phylum	domain
YP_001894194.1	Burkholderia phytofirmans	Burkholderia	Burkholderiaceae	Burkholderiales	Betaproteobacteria	Proteobacteria	Bacteria
YP_001888744.1	Burkholderia phytofirmans	Burkholderia	Burkholderiaceae	Burkholderiales	Betaproteobacteria	Proteobacteria	Bacteria
YP_001894271.1	Burkholderia phytofirmans	Burkholderia	Burkholderiaceae	Burkholderiales	Betaproteobacteria	Proteobacteria	Bacteria
YP_001895658.1	Burkholderia phytofirmans	Burkholderia	Burkholderiaceae	Burkholderiales	Betaproteobacteria	Proteobacteria	Bacteria
YP_001064791.1	Burkholderia pseudomallei	Burkholderia	Burkholderiaceae	Burkholderiales	Betaproteobacteria	Proteobacteria	Bacteria
YP_001057543.1	Burkholderia pseudomallei	Burkholderia	Burkholderiaceae	Burkholderiales	Betaproteobacteria	Proteobacteria	Bacteria
YP_332087.1	Burkholderia pseudomallei	Burkholderia	Burkholderiaceae	Burkholderiales	Betaproteobacteria	Proteobacteria	Bacteria
YP_107079.1	Burkholderia pseudomallei	Burkholderia	Burkholderiaceae	Burkholderiales	Betaproteobacteria	Proteobacteria	Bacteria
YP_001061143.1	Burkholderia pseudomallei	Burkholderia	Burkholderiaceae	Burkholderiales	Betaproteobacteria	Proteobacteria	Bacteria
YP_336750.1	Burkholderia pseudomallei	Burkholderia	Burkholderiaceae	Burkholderiales	Betaproteobacteria	Proteobacteria	Bacteria
YP_110109.1	Burkholderia pseudomallei	Burkholderia	Burkholderiaceae	Burkholderiales	Betaproteobacteria	Proteobacteria	Bacteria
YP_001074155.1	Burkholderia pseudomallei	Burkholderia	Burkholderiaceae	Burkholderiales	Betaproteobacteria	Proteobacteria	Bacteria
YP_111902.1	Burkholderia pseudomallei	Burkholderia	Burkholderiaceae	Burkholderiales	Betaproteobacteria	Proteobacteria	Bacteria
YP_001076601.1	Burkholderia pseudomallei	Burkholderia	Burkholderiaceae	Burkholderiales	Betaproteobacteria	Proteobacteria	Bacteria
YP_336150.1	Burkholderia pseudomallei	Burkholderia	Burkholderiaceae	Burkholderiales	Betaproteobacteria	Proteobacteria	Bacteria
YP_001063707.1	Burkholderia pseudomallei	Burkholderia	Burkholderiaceae	Burkholderiales	Betaproteobacteria	Proteobacteria	Bacteria
YP_334218.1	Burkholderia pseudomallei	Burkholderia	Burkholderiaceae	Burkholderiales	Betaproteobacteria	Proteobacteria	Bacteria
YP_108972.1	Burkholderia pseudomallei	Burkholderia	Burkholderiaceae	Burkholderiales	Betaproteobacteria	Proteobacteria	Bacteria
YP_001067024.1	Burkholderia pseudomallei	Burkholderia	Burkholderiaceae	Burkholderiales	Betaproteobacteria	Proteobacteria	Bacteria
YP_001059739.1	Burkholderia pseudomallei	Burkholderia	Burkholderiaceae	Burkholderiales	Betaproteobacteria	Proteobacteria	Bacteria
YP_001065632.1	Burkholderia pseudomallei	Burkholderia	Burkholderiaceae	Burkholderiales	Betaproteobacteria	Proteobacteria	Bacteria
YP_107881.1	Burkholderia pseudomallei	Burkholderia	Burkholderiaceae	Burkholderiales	Betaproteobacteria	Proteobacteria	Bacteria
YP_332896.1	Burkholderia pseudomallei	Burkholderia	Burkholderiaceae	Burkholderiales	Betaproteobacteria	Proteobacteria	Bacteria
YP_001058396.1	Burkholderia pseudomallei	Burkholderia	Burkholderiaceae	Burkholderiales	Betaproteobacteria	Proteobacteria	Bacteria
YP_001057822.1	Burkholderia pseudomallei	Burkholderia	Burkholderiaceae	Burkholderiales	Betaproteobacteria	Proteobacteria	Bacteria
YP_107348.1	Burkholderia pseudomallei	Burkholderia	Burkholderiaceae	Burkholderiales	Betaproteobacteria	Proteobacteria	Bacteria
YP_332357.1	Burkholderia pseudomallei	Burkholderia	Burkholderiaceae	Burkholderiales	Betaproteobacteria	Proteobacteria	Bacteria
YP_001065064.1	Burkholderia pseudomallei	Burkholderia	Burkholderiaceae	Burkholderiales	Betaproteobacteria	Proteobacteria	Bacteria
YP_001066989.1	Burkholderia pseudomallei	Burkholderia	Burkholderiaceae	Burkholderiales	Betaproteobacteria	Proteobacteria	Bacteria
YP_108946.1	Burkholderia pseudomallei	Burkholderia	Burkholderiaceae	Burkholderiales	Betaproteobacteria	Proteobacteria	Bacteria
YP_334186.1	Burkholderia pseudomallei	Burkholderia	Burkholderiaceae	Burkholderiales	Betaproteobacteria	Proteobacteria	Bacteria
ZP_02482537.1	Burkholderia pseudomallei	Burkholderia	Burkholderiaceae	Burkholderiales	Betaproteobacteria	Proteobacteria	Bacteria
YP_001059704.1	Burkholderia pseudomallei	Burkholderia	Burkholderiaceae	Burkholderiales	Betaproteobacteria	Proteobacteria	Bacteria
YP_001063055.1	Burkholderia pseudomallei	Burkholderia	Burkholderiaceae	Burkholderiales	Betaproteobacteria	Proteobacteria	Bacteria
YP_001075999.1	Burkholderia pseudomallei	Burkholderia	Burkholderiaceae	Burkholderiales	Betaproteobacteria	Proteobacteria	Bacteria
YP_370413.1	Burkholderia sp. 383	Burkholderia	Burkholderiaceae	Burkholderiales	Betaproteobacteria	Proteobacteria	Bacteria
YP_367049.1	Burkholderia sp. 383	Burkholderia	Burkholderiaceae	Burkholderiales	Betaproteobacteria	Proteobacteria	Bacteria
YP_369613.1	Burkholderia sp. 383	Burkholderia	Burkholderiaceae	Burkholderiales	Betaproteobacteria	Proteobacteria	Bacteria

YP_372161.1	Burkholderia sp. 383	Burkholderia	Burkholderiaceae	Burkholderiales	Betaproteobacteria	Proteobacteria	Bacteria
ZP_03268598.1	Burkholderia sp. H160	Burkholderia	Burkholderiaceae	Burkholderiales	Betaproteobacteria	Proteobacteria	Bacteria
ZP_03268378.1	Burkholderia sp. H160	Burkholderia	Burkholderiaceae	Burkholderiales	Betaproteobacteria	Proteobacteria	Bacteria
ZP_03267566.1	Burkholderia sp. H160	Burkholderia	Burkholderiaceae	Burkholderiales	Betaproteobacteria	Proteobacteria	Bacteria
ZP_03268689.1	Burkholderia sp. H160	Burkholderia	Burkholderiaceae	Burkholderiales	Betaproteobacteria	Proteobacteria	Bacteria
ZP_03264540.1	Burkholderia sp. H160	Burkholderia	Burkholderiaceae	Burkholderiales	Betaproteobacteria	Proteobacteria	Bacteria
YP_440985.1	Burkholderia thailandensis	Burkholderia	Burkholderiaceae	Burkholderiales	Betaproteobacteria	Proteobacteria	Bacteria
YP_438680.1	Burkholderia thailandensis	Burkholderia	Burkholderiaceae	Burkholderiales	Betaproteobacteria	Proteobacteria	Bacteria
YP_442319.1	Burkholderia thailandensis	Burkholderia	Burkholderiaceae	Burkholderiales	Betaproteobacteria	Proteobacteria	Bacteria
YP_443386.1	Burkholderia thailandensis	Burkholderia	Burkholderiaceae	Burkholderiales	Betaproteobacteria	Proteobacteria	Bacteria
YP_439812.1	Burkholderia thailandensis	Burkholderia	Burkholderiaceae	Burkholderiales	Betaproteobacteria	Proteobacteria	Bacteria
YP_442345.1	Burkholderia thailandensis	Burkholderia	Burkholderiaceae	Burkholderiales	Betaproteobacteria	Proteobacteria	Bacteria
YP_439142.1	Burkholderia thailandensis	Burkholderia	Burkholderiaceae	Burkholderiales	Betaproteobacteria	Proteobacteria	Bacteria
ZP_02377758.1	Burkholderia ubonensis	Burkholderia	Burkholderiaceae	Burkholderiales	Betaproteobacteria	Proteobacteria	Bacteria
ZP_02382486.1	Burkholderia ubonensis	Burkholderia	Burkholderiaceae	Burkholderiales	Betaproteobacteria	Proteobacteria	Bacteria
ZP_02381572.1	Burkholderia ubonensis	Burkholderia	Burkholderiaceae	Burkholderiales	Betaproteobacteria	Proteobacteria	Bacteria
ZP_02377878.1	Burkholderia ubonensis	Burkholderia	Burkholderiaceae	Burkholderiales	Betaproteobacteria	Proteobacteria	Bacteria
YP_001120772.1	Burkholderia vietnamiensis	Burkholderia	Burkholderiaceae	Burkholderiales	Betaproteobacteria	Proteobacteria	Bacteria
YP_001119810.1	Burkholderia vietnamiensis	Burkholderia	Burkholderiaceae	Burkholderiales	Betaproteobacteria	Proteobacteria	Bacteria
YP_001120648.1	Burkholderia vietnamiensis	Burkholderia	Burkholderiaceae	Burkholderiales	Betaproteobacteria	Proteobacteria	Bacteria
YP_001115325.1	Burkholderia vietnamiensis	Burkholderia	Burkholderiaceae	Burkholderiales	Betaproteobacteria	Proteobacteria	Bacteria
YP_001115017.1	Burkholderia vietnamiensis	Burkholderia	Burkholderiaceae	Burkholderiales	Betaproteobacteria	Proteobacteria	Bacteria

Accession	species	genus	order	classe	family	phylum	domain
YP_556882.1	Burkholderia xenovorans	Burkholderia	Burkholderiaceae	Burkholderiales	Betaproteobacteria	Proteobacteria	Bacteria
YP_553679.1	Burkholderia xenovorans	Burkholderia	Burkholderiaceae	Burkholderiales	Betaproteobacteria	Proteobacteria	Bacteria
YP_555443.1	Burkholderia xenovorans	Burkholderia	Burkholderiaceae	Burkholderiales	Betaproteobacteria	Proteobacteria	Bacteria
YP_556973.1	Burkholderia xenovorans	Burkholderia	Burkholderiaceae	Burkholderiales	Betaproteobacteria	Proteobacteria	Bacteria
YP_555356.1	Burkholderia xenovorans	Burkholderia	Burkholderiaceae	Burkholderiales	Betaproteobacteria	Proteobacteria	Bacteria
YP_558837.1	Burkholderia xenovorans	Burkholderia	Burkholderiaceae	Burkholderiales	Betaproteobacteria	Proteobacteria	Bacteria
YP_001541744.1	Caldivirga maquilingensis	Caldivirga	Thermoproteaceae	Thermoproteales	Thermoprotei	Crenarchaeota	Archaea
YP_001540124.1	Caldivirga maquilingensis	Caldivirga	Thermoproteaceae	Thermoproteales	Thermoprotei	Crenarchaeota	Archaea
YP_001218898.1	Calyptogena okutanii thioautotrophic				Gammaproteobacteria	Proteobacteria	Bacteria
YP_001218903.1	Calyptogena okutanii thioautotrophic				Gammaproteobacteria	Proteobacteria	Bacteria
ZP_00368201.1	Campylobacter coli	Campylobacter	Campylobacteraceae	Campylobacterales	Epsilonproteobacteria	Proteobacteria	Bacteria
ABW74731.1	Campylobacter concisus	Campylobacter	Campylobacteraceae	Campylobacterales	Epsilonproteobacteria	Proteobacteria	Bacteria
YP_001466413.1	Campylobacter concisus	Campylobacter	Campylobacteraceae	Campylobacterales	Epsilonproteobacteria	Proteobacteria	Bacteria
YP_001407573.1	Campylobacter curvus	Campylobacter	Campylobacteraceae	Campylobacterales	Epsilonproteobacteria	Proteobacteria	Bacteria
YP_001407973.1	Campylobacter curvus	Campylobacter	Campylobacteraceae	Campylobacterales	Epsilonproteobacteria	Proteobacteria	Bacteria
YP_891580.1	Campylobacter fetus	Campylobacter	Campylobacteraceae	Campylobacterales	Epsilonproteobacteria	Proteobacteria	Bacteria

YP_001406948.1	Campylobacter hominis	Campylobacter	Campylobacteraceae	Campylobacterales	Epsilonproteobacteria	Proteobacteria	Bacteria
YP_001001138.1	Campylobacter jejuni	Campylobacter	Campylobacteraceae	Campylobacterales	Epsilonproteobacteria	Proteobacteria	Bacteria
YP_002344870.1	Campylobacter jejuni	Campylobacter	Campylobacteraceae	Campylobacterales	Epsilonproteobacteria	Proteobacteria	Bacteria
YP_001482970.1	Campylobacter jejuni	Campylobacter	Campylobacteraceae	Campylobacterales	Epsilonproteobacteria	Proteobacteria	Bacteria
YP_179644.1	Campylobacter jejuni	Campylobacter	Campylobacteraceae	Campylobacterales	Epsilonproteobacteria	Proteobacteria	Bacteria
YP_001398790.1	Campylobacter jejuni	Campylobacter	Campylobacteraceae	Campylobacterales	Epsilonproteobacteria	Proteobacteria	Bacteria
ZP_00368853.1	Campylobacter lari	Campylobacter	Campylobacteraceae	Campylobacterales	Epsilonproteobacteria	Proteobacteria	Bacteria
ZP_00370506.1	Campylobacter upsaliensis	Campylobacter	Campylobacteraceae	Campylobacterales	Epsilonproteobacteria	Proteobacteria	Bacteria
EDZ63164.1	Campylobacterales bacterium GD 1			Campylobacterales	Epsilonproteobacteria	Proteobacteria	Bacteria
EDZ63129.1	Campylobacterales bacterium GD 1			Campylobacterales	Epsilonproteobacteria	Proteobacteria	Bacteria
YP_588718.1	Candidatus Baumannia cicadellinicola				Gammaproteobacteria	Proteobacteria	Bacteria
NP_878541.1	Candidatus Blochmannia floridanus	Candidatus Blochma	Enterobacteriaceae	Enterobacteriales	Gammaproteobacteria	Proteobacteria	Bacteria
YP_277755.1	Candidatus Blochmannia pennsylvanicus	Candidatus Blochma	Enterobacteriaceae	Enterobacteriales	Gammaproteobacteria	Proteobacteria	Bacteria
YP_802566.1	Candidatus Carsonella ruddii	Candidatus Carsonella			Gammaproteobacteria	Proteobacteria	Bacteria
CAJ71174.1	Candidatus Kuenenia stuttgartiensis	Candidatus Kuenenia		Planctomycetales	Planctomycetacia	Planctomycetes	Bacteria
ZP_03287165.1	Candidatus Liberibacter asiaticus	Candidatus Liberibac	Rhizobiaceae	Rhizobiales	Alphaproteobacteria	Proteobacteria	Bacteria
YP_265561.1	Candidatus Pelagibacter ubique	Candidatus Pelagibacter		Rickettsiales	Alphaproteobacteria	Proteobacteria	Bacteria
ZP_01264700.1	Candidatus Pelagibacter ubique	Candidatus Pelagibacter		Rickettsiales	Alphaproteobacteria	Proteobacteria	Bacteria
YP_008189.1	Candidatus Protochlamydia amoebophila	Candidatus Protochla	Parachlamydiaceae	Chlamydiales	Chlamydiae (class)	Chlamydiae	Bacteria
YP_008977.1	Candidatus Protochlamydia amoebophila	Candidatus Protochla	Parachlamydiaceae	Chlamydiales	Chlamydiae (class)	Chlamydiae	Bacteria
YP_903309.1	Candidatus Ruthia magnifica				Gammaproteobacteria	Proteobacteria	Bacteria
YP_903313.1	Candidatus Ruthia magnifica				Gammaproteobacteria	Proteobacteria	Bacteria
YP_001597952.1	Candidatus Sulcia muelleri	Candidatus Sulcia		Flavobacteriales	Flavobacteria	Bacteroidetes	Bacteria
YP_001686123.1	Caulobacter sp. K31	Caulobacter	Caulobacteraceae	Caulobacterales	Alphaproteobacteria	Proteobacteria	Bacteria
YP_001684062.1	Caulobacter sp. K31	Caulobacter	Caulobacteraceae	Caulobacterales	Alphaproteobacteria	Proteobacteria	Bacteria
NP_422200.1	Caulobacter vibrioides	Caulobacter	Caulobacteraceae	Caulobacterales	Alphaproteobacteria	Proteobacteria	Bacteria
NP_420580.1	Caulobacter vibrioides	Caulobacter	Caulobacteraceae	Caulobacterales	Alphaproteobacteria	Proteobacteria	Bacteria
NP_420214.1	Caulobacter vibrioides	Caulobacter	Caulobacteraceae	Caulobacterales	Alphaproteobacteria	Proteobacteria	Bacteria
YP_001981374.1	Cellvibrio japonicus	Cellvibrio	Pseudomonadaceae	Pseudomonadales	Gammaproteobacteria	Proteobacteria	Bacteria

Accession	species	genus	order	classe	family	phylum	domain
YP_001983094.1	Cellvibrio japonicus	Cellvibrio	Pseudomonadaceae	Pseudomonadales	Gammaproteobacteria	Proteobacteria	Bacteria
YP_001982028.1	Cellvibrio japonicus	Cellvibrio	Pseudomonadaceae	Pseudomonadales	Gammaproteobacteria	Proteobacteria	Bacteria
YP_876890.1	Cenarchaeum symbiosum	Cenarchaeum	Cenarchaeaceae	Cenarchaeales	Thermoprotei	Crenarchaeota	Archaea
YP_001998491.1	Chlorobaculum parvum	Chlorobaculum	Chlorobiaceae	Chlorobiales	Chlorobia	Chlorobi	Bacteria
YP_001999252.1	Chlorobaculum parvum	Chlorobaculum	Chlorobiaceae	Chlorobiales	Chlorobia	Chlorobi	Bacteria
YP_379659.1	Chlorobium chlorochromatii	Chlorobium	Chlorobiaceae	Chlorobiales	Chlorobia	Chlorobi	Bacteria
ZP_01385091.1	Chlorobium ferrooxidans	Chlorobium	Chlorobiaceae	Chlorobiales	Chlorobia	Chlorobi	Bacteria
YP_001943824.1	Chlorobium limicola	Chlorobium	Chlorobiaceae	Chlorobiales	Chlorobia	Chlorobi	Bacteria

YP_001959988.1	Chlorobium phaeobacteroides	Chlorobium	Chlorobiaceae	Chlorobiales	Chlorobia	Chlorobi	Bacteria
YP_002462860.1	Chloroflexus aggregans	Chloroflexus	Chloroflexaceae	Chloroflexales	Chloroflexi (class)	Chloroflexi	Bacteria
ZP_01514910.1	Chloroflexus aggregans	Chloroflexus	Chloroflexaceae	Chloroflexales	Chloroflexi (class)	Chloroflexi	Bacteria
YP_002464262.1	Chloroflexus aggregans	Chloroflexus	Chloroflexaceae	Chloroflexales	Chloroflexi (class)	Chloroflexi	Bacteria
ZP_01516700.1	Chloroflexus aggregans	Chloroflexus	Chloroflexaceae	Chloroflexales	Chloroflexi (class)	Chloroflexi	Bacteria
YP_001635743.1	Chloroflexus aurantiacus	Chloroflexus	Chloroflexaceae	Chloroflexales	Chloroflexi (class)	Chloroflexi	Bacteria
YP_001636024.1	Chloroflexus aurantiacus	Chloroflexus	Chloroflexaceae	Chloroflexales	Chloroflexi (class)	Chloroflexi	Bacteria
NP_900270.1	Chromobacterium violaceum	Chromobacterium	Neisseriaceae	Neisseriales	Betaproteobacteria	Proteobacteria	Bacteria
NP_903665.1	Chromobacterium violaceum	Chromobacterium	Neisseriaceae	Neisseriales	Betaproteobacteria	Proteobacteria	Bacteria
NP_900844.1	Chromobacterium violaceum	Chromobacterium	Neisseriaceae	Neisseriales	Betaproteobacteria	Proteobacteria	Bacteria
NP_903164.1	Chromobacterium violaceum	Chromobacterium	Neisseriaceae	Neisseriales	Betaproteobacteria	Proteobacteria	Bacteria
YP_573011.1	Chromohalobacter salexigens	Chromohalobacter	Halomonadaceae	Oceanospirillales	Gammaproteobacteria	Proteobacteria	Bacteria
ZP_03129065.1	Chthoniobacter flavus	Chthoniobacter			Spartobacteria	Verrucomicrobia	Bacteria
ZP_03130087.1	Chthoniobacter flavus	Chthoniobacter			Spartobacteria	Verrucomicrobia	Bacteria
YP_001454272.1	Citrobacter koseri	Citrobacter	Enterobacteriaceae	Enterobacteriales	Gammaproteobacteria	Proteobacteria	Bacteria
YP_001710118.1	Clavibacter michiganensis	Clavibacter	Microbacteriaceae	Actinomycetales	Actinobacteria (class)	Actinobacteria	Bacteria
YP_001222583.1	Clavibacter michiganensis	Clavibacter	Microbacteriaceae	Actinomycetales	Actinobacteria (class)	Actinobacteria	Bacteria
YP_267002.1	Colwellia psychrerythraea	Colwellia	Colwelliaceae	Alteromonadales	Gammaproteobacteria	Proteobacteria	Bacteria
YP_268725.1	Colwellia psychrerythraea	Colwellia	Colwelliaceae	Alteromonadales	Gammaproteobacteria	Proteobacteria	Bacteria
YP_267351.1	Colwellia psychrerythraea	Colwellia	Colwelliaceae	Alteromonadales	Gammaproteobacteria	Proteobacteria	Bacteria
ZP_01517839.1	Comamonas testosteroni	Comamonas	Comamonadaceae	Burkholderiales	Betaproteobacteria	Proteobacteria	Bacteria
ZP_01518706.1	Comamonas testosteroni	Comamonas	Comamonadaceae	Burkholderiales	Betaproteobacteria	Proteobacteria	Bacteria
ZP_01519090.1	Comamonas testosteroni	Comamonas	Comamonadaceae	Burkholderiales	Betaproteobacteria	Proteobacteria	Bacteria
ZP_01103079.1	Congregibacter litoralis	Congregibacter			Gammaproteobacteria	Proteobacteria	Bacteria
ZP_01101285.1	Congregibacter litoralis	Congregibacter			Gammaproteobacteria	Proteobacteria	Bacteria
ZP_01104031.1	Congregibacter litoralis	Congregibacter			Gammaproteobacteria	Proteobacteria	Bacteria
NP_940201.1	Corynebacterium diphtheriae	Corynebacterium	Corynebacteriaceae	Actinomycetales	Actinobacteria (class)	Actinobacteria	Bacteria
NP_940552.1	Corynebacterium diphtheriae	Corynebacterium	Corynebacteriaceae	Actinomycetales	Actinobacteria (class)	Actinobacteria	Bacteria
NP_739028.1	Corynebacterium efficiens	Corynebacterium	Corynebacteriaceae	Actinomycetales	Actinobacteria (class)	Actinobacteria	Bacteria
YP_226765.1	Corynebacterium glutamicum	Corynebacterium	Corynebacteriaceae	Actinomycetales	Actinobacteria (class)	Actinobacteria	Bacteria
BAB64406.1	Corynebacterium glutamicum	Corynebacterium	Corynebacteriaceae	Actinomycetales	Actinobacteria (class)	Actinobacteria	Bacteria
YP_001139341.1	Corynebacterium glutamicum	Corynebacterium	Corynebacteriaceae	Actinomycetales	Actinobacteria (class)	Actinobacteria	Bacteria
NP_601724.2	Corynebacterium glutamicum	Corynebacterium	Corynebacteriaceae	Actinomycetales	Actinobacteria (class)	Actinobacteria	Bacteria
YP_250256.1	Corynebacterium jeikeium	Corynebacterium	Corynebacteriaceae	Actinomycetales	Actinobacteria (class)	Actinobacteria	Bacteria
YP_001800872.1	Corynebacterium urealyticum	Corynebacterium	Corynebacteriaceae	Actinomycetales	Actinobacteria (class)	Actinobacteria	Bacteria
YP_001424381.1	Coxiella burnetii	Coxiella	Coxiellaceae	Legionellales	Gammaproteobacteria	Proteobacteria	Bacteria
YP_001596689.1	Coxiella burnetii	Coxiella	Coxiellaceae	Legionellales	Gammaproteobacteria	Proteobacteria	Bacteria

Accession	species	genus	order	classe	family	phylum	domain
NP_820041.1	Coxiella burnetii	Coxiella	Coxiellaceae	Legionellales	Gammaproteobacteria	Proteobacteria	Bacteria

YP_002305192.1	Coxiella burnetii	Coxiella	Coxiellaceae	Legionellales	Gammaproteobacteria	Proteobacteria	Bacteria
YP_002303478.1	Coxiella burnetii	Coxiella	Coxiellaceae	Legionellales	Gammaproteobacteria	Proteobacteria	Bacteria
ZP_00949206.1	Croceibacter atlanticus	Croceibacter	Flavobacteriaceae	Flavobacteriales	Flavobacteria	Bacteroidetes	Bacteria
ZP_00514441.1	Crocosphaera watsonii	Crocosphaera		Chroococcales		Cyanobacteria	Bacteria
ZP_00515880.1	Crocosphaera watsonii	Crocosphaera		Chroococcales		Cyanobacteria	Bacteria
YP_001438936.1	Cronobacter sakazakii	Cronobacter	Enterobacteriaceae	Enterobacteriales	Gammaproteobacteria	Proteobacteria	Bacteria
YP_582417.1	Cupriavidus metallidurans	Cupriavidus	Burkholderiaceae	Burkholderiales	Betaproteobacteria	Proteobacteria	Bacteria
YP_586093.1	Cupriavidus metallidurans	Cupriavidus	Burkholderiaceae	Burkholderiales	Betaproteobacteria	Proteobacteria	Bacteria
YP_587920.1	Cupriavidus metallidurans	Cupriavidus	Burkholderiaceae	Burkholderiales	Betaproteobacteria	Proteobacteria	Bacteria
YP_583104.1	Cupriavidus metallidurans	Cupriavidus	Burkholderiaceae	Burkholderiales	Betaproteobacteria	Proteobacteria	Bacteria
YP_587737.1	Cupriavidus metallidurans	Cupriavidus	Burkholderiaceae	Burkholderiales	Betaproteobacteria	Proteobacteria	Bacteria
YP_584192.1	Cupriavidus metallidurans	Cupriavidus	Burkholderiaceae	Burkholderiales	Betaproteobacteria	Proteobacteria	Bacteria
YP_584521.1	Cupriavidus metallidurans	Cupriavidus	Burkholderiaceae	Burkholderiales	Betaproteobacteria	Proteobacteria	Bacteria
YP_586456.1	Cupriavidus metallidurans	Cupriavidus	Burkholderiaceae	Burkholderiales	Betaproteobacteria	Proteobacteria	Bacteria
YP_585308.1	Cupriavidus metallidurans	Cupriavidus	Burkholderiaceae	Burkholderiales	Betaproteobacteria	Proteobacteria	Bacteria
YP_724862.1	Cupriavidus necator	Cupriavidus	Burkholderiaceae	Burkholderiales	Betaproteobacteria	Proteobacteria	Bacteria
YP_841573.1	Cupriavidus necator	Cupriavidus	Burkholderiaceae	Burkholderiales	Betaproteobacteria	Proteobacteria	Bacteria
YP_726142.1	Cupriavidus necator	Cupriavidus	Burkholderiaceae	Burkholderiales	Betaproteobacteria	Proteobacteria	Bacteria
YP_725581.1	Cupriavidus necator	Cupriavidus	Burkholderiaceae	Burkholderiales	Betaproteobacteria	Proteobacteria	Bacteria
YP_729182.1	Cupriavidus necator	Cupriavidus	Burkholderiaceae	Burkholderiales	Betaproteobacteria	Proteobacteria	Bacteria
YP_726783.1	Cupriavidus necator	Cupriavidus	Burkholderiaceae	Burkholderiales	Betaproteobacteria	Proteobacteria	Bacteria
YP_841835.1	Cupriavidus necator	Cupriavidus	Burkholderiaceae	Burkholderiales	Betaproteobacteria	Proteobacteria	Bacteria
AAC45803.1	Cupriavidus necator	Cupriavidus	Burkholderiaceae	Burkholderiales	Betaproteobacteria	Proteobacteria	Bacteria
YP_294541.1	Cupriavidus pinatubonensis	Cupriavidus	Burkholderiaceae	Burkholderiales	Betaproteobacteria	Proteobacteria	Bacteria
YP_297835.1	Cupriavidus pinatubonensis	Cupriavidus	Burkholderiaceae	Burkholderiales	Betaproteobacteria	Proteobacteria	Bacteria
YP_298772.1	Cupriavidus pinatubonensis	Cupriavidus	Burkholderiaceae	Burkholderiales	Betaproteobacteria	Proteobacteria	Bacteria
YP_295206.1	Cupriavidus pinatubonensis	Cupriavidus	Burkholderiaceae	Burkholderiales	Betaproteobacteria	Proteobacteria	Bacteria
YP_299083.1	Cupriavidus pinatubonensis	Cupriavidus	Burkholderiaceae	Burkholderiales	Betaproteobacteria	Proteobacteria	Bacteria
YP_298631.1	Cupriavidus pinatubonensis	Cupriavidus	Burkholderiaceae	Burkholderiales	Betaproteobacteria	Proteobacteria	Bacteria
YP_296249.1	Cupriavidus pinatubonensis	Cupriavidus	Burkholderiaceae	Burkholderiales	Betaproteobacteria	Proteobacteria	Bacteria
YP_299247.1	Cupriavidus pinatubonensis	Cupriavidus	Burkholderiaceae	Burkholderiales	Betaproteobacteria	Proteobacteria	Bacteria
YP_001795680.1	Cupriavidus taiwanensis	Cupriavidus	Burkholderiaceae	Burkholderiales	Betaproteobacteria	Proteobacteria	Bacteria
YP_002008421.1	Cupriavidus taiwanensis	Cupriavidus	Burkholderiaceae	Burkholderiales	Betaproteobacteria	Proteobacteria	Bacteria
YP_002005088.1	Cupriavidus taiwanensis	Cupriavidus	Burkholderiaceae	Burkholderiales	Betaproteobacteria	Proteobacteria	Bacteria
YP_002008864.1	Cupriavidus taiwanensis	Cupriavidus	Burkholderiaceae	Burkholderiales	Betaproteobacteria	Proteobacteria	Bacteria
YP_002005870.1	Cupriavidus taiwanensis	Cupriavidus	Burkholderiaceae	Burkholderiales	Betaproteobacteria	Proteobacteria	Bacteria
YP_002008716.1	Cupriavidus taiwanensis	Cupriavidus	Burkholderiaceae	Burkholderiales	Betaproteobacteria	Proteobacteria	Bacteria
EDY38696.1	Cyanobium sp. PCC 7001	Cyanobium		Chroococcales		Cyanobacteria	Bacteria
EDY39380.1	Cyanobium sp. PCC 7001	Cyanobium		Chroococcales		Cyanobacteria	Bacteria
YP_001806228.1	Cyanothece sp. ATCC 51142	Cyanothece		Chroococcales		Cyanobacteria	Bacteria
YP_001805524.1	Cyanothece sp. ATCC 51142	Cyanothece		Chroococcales		Cyanobacteria	Bacteria
YP_001803392.1	Cyanothece sp. ATCC 51142	Cyanothece		Chroococcales		Cyanobacteria	Bacteria
YP_002378410.1	Cyanothece sp. PCC 7424	Cyanothece		Chroococcales		Cyanobacteria	Bacteria

Accession	species	genus	order	classe	family	phylum	domain
YP_002379313.1	Cyanothece sp. PCC 7424	Cyanothece		Chroococcales		Cyanobacteria	Bacteria
YP_002370814.1	Cyanothece sp. PCC 8801	Cyanothece		Chroococcales		Cyanobacteria	Bacteria
YP_678811.1	Cytophaga hutchinsonii	Cytophaga	Flexibacteraceae	Sphingobacteriales	Sphingobacteria	Bacteroidetes	Bacteria
YP_677753.1	Cytophaga hutchinsonii	Cytophaga	Flexibacteraceae	Sphingobacteriales	Sphingobacteria	Bacteroidetes	Bacteria
YP_284689.1	Dechloromonas aromatica	Dechloromonas	Rhodocyclaceae	Rhodocyclales	Betaproteobacteria	Proteobacteria	Bacteria
YP_283939.1	Dechloromonas aromatica	Dechloromonas	Rhodocyclaceae	Rhodocyclales	Betaproteobacteria	Proteobacteria	Bacteria
YP_286391.1	Dechloromonas aromatica	Dechloromonas	Rhodocyclaceae	Rhodocyclales	Betaproteobacteria	Proteobacteria	Bacteria
YP_603878.1	Deinococcus geothermalis	Deinococcus	Deinococcaceae	Deinococcales	Deinococci	Deinococcus-Thermus	Bacteria
YP_603490.1	Deinococcus geothermalis	Deinococcus	Deinococcaceae	Deinococcales	Deinococci	Deinococcus-Thermus	Bacteria
NP_296339.1	Deinococcus radiodurans	Deinococcus	Deinococcaceae	Deinococcales	Deinococci	Deinococcus-Thermus	Bacteria
YP_001562703.1	Delftia acidovorans	Delftia	Comamonadaceae	Burkholderiales	Betaproteobacteria	Proteobacteria	Bacteria
YP_001565074.1	Delftia acidovorans	Delftia	Comamonadaceae	Burkholderiales	Betaproteobacteria	Proteobacteria	Bacteria
YP_001563798.1	Delftia acidovorans	Delftia	Comamonadaceae	Burkholderiales	Betaproteobacteria	Proteobacteria	Bacteria
ZP_01287602.1	delta proteobacterium MLMS-1				Deltaproteobacteria	Proteobacteria	Bacteria
YP_002458720.1	Desulfitobacterium hafniense	Desulfitobacterium	Peptococcaceae	Clostridiales	Clostridia	Firmicutes	Bacteria
YP_517398.1	Desulfitobacterium hafniense	Desulfitobacterium	Peptococcaceae	Clostridiales	Clostridia	Firmicutes	Bacteria
YP_388315.1	Desulfovibrio desulfuricans	Desulfovibrio	Desulfovibrionaceae	Desulfovibrionales	Deltaproteobacteria	Proteobacteria	Bacteria
BAA06976.1	Desulfovibrio vulgaris	Desulfovibrio	Desulfovibrionaceae	Desulfovibrionales	Deltaproteobacteria	Proteobacteria	Bacteria
YP_966790.1	Desulfovibrio vulgaris	Desulfovibrio	Desulfovibrionaceae	Desulfovibrionales	Deltaproteobacteria	Proteobacteria	Bacteria
YP_011033.1	Desulfovibrio vulgaris	Desulfovibrio	Desulfovibrionaceae	Desulfovibrionales	Deltaproteobacteria	Proteobacteria	Bacteria
YP_002434961.1	Desulfovibrio vulgaris	Desulfovibrio	Desulfovibrionaceae	Desulfovibrionales	Deltaproteobacteria	Proteobacteria	Bacteria
YP_001533720.1	Dinoroseobacter shibae	Dinoroseobacter	Rhodobacteraceae	Rhodobacterales	Alphaproteobacteria	Proteobacteria	Bacteria
YP_001542100.1	Dinoroseobacter shibae	Dinoroseobacter	Rhodobacteraceae	Rhodobacterales	Alphaproteobacteria	Proteobacteria	Bacteria
YP_001532008.1	Dinoroseobacter shibae	Dinoroseobacter	Rhodobacteraceae	Rhodobacterales	Alphaproteobacteria	Proteobacteria	Bacteria
YP_001534518.1	Dinoroseobacter shibae	Dinoroseobacter	Rhodobacteraceae	Rhodobacterales	Alphaproteobacteria	Proteobacteria	Bacteria
ZP_01050582.1	Dokdonia donghaensis	Dokdonia	Flavobacteriaceae	Flavobacteriales	Flavobacteria	Bacteroidetes	Bacteria
ZP_01050474.1	Dokdonia donghaensis	Dokdonia	Flavobacteriaceae	Flavobacteriales	Flavobacteria	Bacteroidetes	Bacteria
YP_303439.1	Ehrlichia canis	Ehrlichia	Anaplasmataceae	Rickettsiales	Alphaproteobacteria	Proteobacteria	Bacteria
YP_507790.1	Ehrlichia chaffeensis	Ehrlichia	Anaplasmataceae	Rickettsiales	Alphaproteobacteria	Proteobacteria	Bacteria
YP_196734.1	Ehrlichia ruminantium	Ehrlichia	Anaplasmataceae	Rickettsiales	Alphaproteobacteria	Proteobacteria	Bacteria
YP_197694.1	Ehrlichia ruminantium	Ehrlichia	Anaplasmataceae	Rickettsiales	Alphaproteobacteria	Proteobacteria	Bacteria
YP_180637.1	Ehrlichia ruminantium	Ehrlichia	Anaplasmataceae	Rickettsiales	Alphaproteobacteria	Proteobacteria	Bacteria
ZP_03281366.1	Enterobacter cancerogenus	Enterobacter	Enterobacteriaceae	Enterobacteriales	Gammaproteobacteria	Proteobacteria	Bacteria
YP_001175633.1	Enterobacter sp. 638	Enterobacter	Enterobacteriaceae	Enterobacteriales	Gammaproteobacteria	Proteobacteria	Bacteria
YP_001908438.1	Erwinia tasmaniensis	Erwinia	Enterobacteriaceae	Enterobacteriales	Gammaproteobacteria	Proteobacteria	Bacteria
YP_457435.1	Erythrobacter litoralis	Erythrobacter	Erythrobacteraceae	Sphingomonadales	Alphaproteobacteria	Proteobacteria	Bacteria

ZP_01041429.1	Erythrobacter sp. NAP1	Erythrobacter	Erythrobacteraceae	Sphingomonadales	Alphaproteobacteria	Proteobacteria	Bacteria
ZP_01864317.1	Erythrobacter sp. SD-21	Erythrobacter	Erythrobacteraceae	Sphingomonadales	Alphaproteobacteria	Proteobacteria	Bacteria
ZP_01865010.1	Erythrobacter sp. SD-21	Erythrobacter	Erythrobacteraceae	Sphingomonadales	Alphaproteobacteria	Proteobacteria	Bacteria
ZP_02902141.1	Escherichia albertii	Escherichia	Enterobacteriaceae	Enterobacteriales	Gammaproteobacteria	Proteobacteria	Bacteria
YP_002396506.1	Escherichia coli	Escherichia	Enterobacteriaceae	Enterobacteriales	Gammaproteobacteria	Proteobacteria	Bacteria
YP_002291731.1	Escherichia coli	Escherichia	Enterobacteriaceae	Enterobacteriales	Gammaproteobacteria	Proteobacteria	Bacteria
YP_002401561.1	Escherichia coli	Escherichia	Enterobacteriaceae	Enterobacteriales	Gammaproteobacteria	Proteobacteria	Bacteria
YP_002390253.1	Escherichia coli	Escherichia	Enterobacteriaceae	Enterobacteriales	Gammaproteobacteria	Proteobacteria	Bacteria
YP_002406286.1	Escherichia coli	Escherichia	Enterobacteriaceae	Enterobacteriales	Gammaproteobacteria	Proteobacteria	Bacteria

Accession	species	genus	order	classe	family	phylum	domain
YP_002269077.1	Escherichia coli	Escherichia	Enterobacteriaceae	Enterobacteriales	Gammaproteobacteria	Proteobacteria	Bacteria
YP_539487.1	Escherichia coli	Escherichia	Enterobacteriaceae	Enterobacteriales	Gammaproteobacteria	Proteobacteria	Bacteria
NP_308512.1	Escherichia coli	Escherichia	Enterobacteriaceae	Enterobacteriales	Gammaproteobacteria	Proteobacteria	Bacteria
YP_001729337.1	Escherichia coli	Escherichia	Enterobacteriaceae	Enterobacteriales	Gammaproteobacteria	Proteobacteria	Bacteria
YP_001726151.1	Escherichia coli	Escherichia	Enterobacteriaceae	Enterobacteriales	Gammaproteobacteria	Proteobacteria	Bacteria
NP_752476.1	Escherichia coli	Escherichia	Enterobacteriaceae	Enterobacteriales	Gammaproteobacteria	Proteobacteria	Bacteria
NP_286173.1	Escherichia coli	Escherichia	Enterobacteriaceae	Enterobacteriales	Gammaproteobacteria	Proteobacteria	Bacteria
YP_001461617.1	Escherichia coli	Escherichia	Enterobacteriaceae	Enterobacteriales	Gammaproteobacteria	Proteobacteria	Bacteria
YP_001457274.1	Escherichia coli	Escherichia	Enterobacteriaceae	Enterobacteriales	Gammaproteobacteria	Proteobacteria	Bacteria
YP_002327943.1	Escherichia coli	Escherichia	Enterobacteriaceae	Enterobacteriales	Gammaproteobacteria	Proteobacteria	Bacteria
YP_851604.1	Escherichia coli	Escherichia	Enterobacteriaceae	Enterobacteriales	Gammaproteobacteria	Proteobacteria	Bacteria
YP_002385910.1	Escherichia coli	Escherichia	Enterobacteriaceae	Enterobacteriales	Gammaproteobacteria	Proteobacteria	Bacteria
YP_002411233.1	Escherichia coli	Escherichia	Enterobacteriaceae	Enterobacteriales	Gammaproteobacteria	Proteobacteria	Bacteria
AP_001081.1	Escherichia coli	Escherichia	Enterobacteriaceae	Enterobacteriales	Gammaproteobacteria	Proteobacteria	Bacteria
NP_414965.1	Escherichia coli	Escherichia	Enterobacteriaceae	Enterobacteriales	Gammaproteobacteria	Proteobacteria	Bacteria
YP_001742572.1	Escherichia coli	Escherichia	Enterobacteriaceae	Enterobacteriales	Gammaproteobacteria	Proteobacteria	Bacteria
YP_668419.1	Escherichia coli	Escherichia	Enterobacteriaceae	Enterobacteriales	Gammaproteobacteria	Proteobacteria	Bacteria
YP_002383692.1	Escherichia fergusonii	Escherichia	Enterobacteriaceae	Enterobacteriales	Gammaproteobacteria	Proteobacteria	Bacteria
YP_001814456.1	Exiguobacterium sibiricum	Exiguobacterium		Bacillales	Bacilli	Firmicutes	Bacteria
YP_001813096.1	Exiguobacterium sibiricum	Exiguobacterium		Bacillales	Bacilli	Firmicutes	Bacteria
ZP_02991613.1	Exiguobacterium sp. AT1b	Exiguobacterium		Bacillales	Bacilli	Firmicutes	Bacteria
ZP_02992279.1	Exiguobacterium sp. AT1b	Exiguobacterium		Bacillales	Bacilli	Firmicutes	Bacteria
ZP_01734088.1	Flavobacteria bacterium BAL38			Flavobacteriales	Flavobacteria	Bacteroidetes	Bacteria
ZP_01734935.1	Flavobacteria bacterium BAL38			Flavobacteriales	Flavobacteria	Bacteroidetes	Bacteria
ZP_02180989.1	Flavobacteriales bacterium ALC-1			Flavobacteriales	Flavobacteria	Bacteroidetes	Bacteria
ZP_01106280.1	Flavobacteriales bacterium HTCC2170			Flavobacteriales	Flavobacteria	Bacteroidetes	Bacteria
YP_001193992.1	Flavobacterium johnsoniae	Flavobacterium	Flavobacteriaceae	Flavobacteriales	Flavobacteria	Bacteroidetes	Bacteria
YP_001194883.1	Flavobacterium johnsoniae	Flavobacterium	Flavobacteriaceae	Flavobacteriales	Flavobacteria	Bacteroidetes	Bacteria
YP_001194759.1	Flavobacterium johnsoniae	Flavobacterium	Flavobacteriaceae	Flavobacteriales	Flavobacteria	Bacteroidetes	Bacteria

YP_001295310.1	Flavobacterium psychrophilum	Flavobacterium	Flavobacteriaceae	Flavobacteriales	Flavobacteria	Bacteroidetes	Bacteria
YP_001296054.1	Flavobacterium psychrophilum	Flavobacterium	Flavobacteriaceae	Flavobacteriales	Flavobacteria	Bacteroidetes	Bacteria
YP_001295831.1	Flavobacterium psychrophilum	Flavobacterium	Flavobacteriaceae	Flavobacteriales	Flavobacteria	Bacteroidetes	Bacteria
YP_897860.1	Francisella novicida	Francisella	Francisellaceae	Thiotrichales	Gammaproteobacteria	Proteobacteria	Bacteria
ZP_03247914.1	Francisella novicida	Francisella	Francisellaceae	Thiotrichales	Gammaproteobacteria	Proteobacteria	Bacteria
YP_001122595.1	Francisella tularensis	Francisella	Francisellaceae	Thiotrichales	Gammaproteobacteria	Proteobacteria	Bacteria
YP_666464.1	Francisella tularensis	Francisella	Francisellaceae	Thiotrichales	Gammaproteobacteria	Proteobacteria	Bacteria
YP_169332.1	Francisella tularensis	Francisella	Francisellaceae	Thiotrichales	Gammaproteobacteria	Proteobacteria	Bacteria
YP_762853.1	Francisella tularensis	Francisella	Francisellaceae	Thiotrichales	Gammaproteobacteria	Proteobacteria	Bacteria
YP_512987.1	Francisella tularensis	Francisella	Francisellaceae	Thiotrichales	Gammaproteobacteria	Proteobacteria	Bacteria
YP_001892168.1	Francisella tularensis	Francisella	Francisellaceae	Thiotrichales	Gammaproteobacteria	Proteobacteria	Bacteria
YP_002167604.1	Francisella tularensis	Francisella	Francisellaceae	Thiotrichales	Gammaproteobacteria	Proteobacteria	Bacteria
YP_001427640.1	Francisella tularensis	Francisella	Francisellaceae	Thiotrichales	Gammaproteobacteria	Proteobacteria	Bacteria
YP_716216.1	Frankia alni	Frankia	Frankiaceae	Actinomycetales	Actinobacteria (class)	Actinobacteria	Bacteria
YP_715300.1	Frankia alni	Frankia	Frankiaceae	Actinomycetales	Actinobacteria (class)	Actinobacteria	Bacteria

Accession	species	genus	order	classe	family	phylum	domain
YP_714342.1	Frankia alni	Frankia	Frankiaceae	Actinomycetales	Actinobacteria (class)	Actinobacteria	Bacteria
YP_481589.1	Frankia sp. CcI3	Frankia	Frankiaceae	Actinomycetales	Actinobacteria (class)	Actinobacteria	Bacteria
YP_482202.1	Frankia sp. CcI3	Frankia	Frankiaceae	Actinomycetales	Actinobacteria (class)	Actinobacteria	Bacteria
YP_482900.1	Frankia sp. CcI3	Frankia	Frankiaceae	Actinomycetales	Actinobacteria (class)	Actinobacteria	Bacteria
YP_481112.1	Frankia sp. CcI3	Frankia	Frankiaceae	Actinomycetales	Actinobacteria (class)	Actinobacteria	Bacteria
YP_001505257.1	Frankia sp. EAN1pec	Frankia	Frankiaceae	Actinomycetales	Actinobacteria (class)	Actinobacteria	Bacteria
YP_001506146.1	Frankia sp. EAN1pec	Frankia	Frankiaceae	Actinomycetales	Actinobacteria (class)	Actinobacteria	Bacteria
ZP_01440921.1	Fulvimarina pelagi	Fulvimarina	Aurantimonadaceae	Rhizobiales	Alphaproteobacteria	Proteobacteria	Bacteria
ZP_01440869.1	Fulvimarina pelagi	Fulvimarina	Aurantimonadaceae	Rhizobiales	Alphaproteobacteria	Proteobacteria	Bacteria
ZP_01438693.1	Fulvimarina pelagi	Fulvimarina	Aurantimonadaceae	Rhizobiales	Alphaproteobacteria	Proteobacteria	Bacteria
ZP_01223355.1	gamma proteobacterium HTCC2207				Gammaproteobacteria	Proteobacteria	Bacteria
ZP_02733610.1	Gemmata obscuriglobus	Gemmata	Planctomycetaceae	Planctomycetales	Planctomycetacia	Planctomycetes	Bacteria
ZP_02737089.1	Gemmata obscuriglobus	Gemmata	Planctomycetaceae	Planctomycetales	Planctomycetacia	Planctomycetes	Bacteria
YP_149311.1	Geobacillus kaustophilus	Geobacillus	Bacillaceae	Bacillales	Bacilli	Firmicutes	Bacteria
YP_146936.1	Geobacillus kaustophilus	Geobacillus	Bacillaceae	Bacillales	Bacilli	Firmicutes	Bacteria
YP_147399.1	Geobacillus kaustophilus	Geobacillus	Bacillaceae	Bacillales	Bacilli	Firmicutes	Bacteria
YP_147524.1	Geobacillus kaustophilus	Geobacillus	Bacillaceae	Bacillales	Bacilli	Firmicutes	Bacteria
YP_146611.1	Geobacillus kaustophilus	Geobacillus	Bacillaceae	Bacillales	Bacilli	Firmicutes	Bacteria
ZP_03146778.1	Geobacillus sp. G11MC16	Geobacillus	Bacillaceae	Bacillales	Bacilli	Firmicutes	Bacteria
ZP_02913585.1	Geobacillus sp. WCH70	Geobacillus	Bacillaceae	Bacillales	Bacilli	Firmicutes	Bacteria
ZP_03036781.1	Geobacillus sp. Y412MC10	Geobacillus	Bacillaceae	Bacillales	Bacilli	Firmicutes	Bacteria

ZP_03037388.1	Geobacillus sp. Y412MC10	Geobacillus	Bacillaceae	Bacillales	Bacilli	Firmicutes	Bacteria
BAA11112.1	Geobacillus stearothermophilus	Geobacillus	Bacillaceae	Bacillales	Bacilli	Firmicutes	Bacteria
BAA35056.1	Geobacillus stearothermophilus	Geobacillus	Bacillaceae	Bacillales	Bacilli	Firmicutes	Bacteria
BAG66139.1	Geobacillus stearothermophilus	Geobacillus	Bacillaceae	Bacillales	Bacilli	Firmicutes	Bacteria
YP_001127472.1	Geobacillus thermodenitrificans	Geobacillus	Bacillaceae	Bacillales	Bacilli	Firmicutes	Bacteria
YP_001125071.1	Geobacillus thermodenitrificans	Geobacillus	Bacillaceae	Bacillales	Bacilli	Firmicutes	Bacteria
YP_001125509.1	Geobacillus thermodenitrificans	Geobacillus	Bacillaceae	Bacillales	Bacilli	Firmicutes	Bacteria
YP_001125633.1	Geobacillus thermodenitrificans	Geobacillus	Bacillaceae	Bacillales	Bacilli	Firmicutes	Bacteria
YP_001124768.1	Geobacillus thermodenitrificans	Geobacillus	Bacillaceae	Bacillales	Bacilli	Firmicutes	Bacteria
YP_002136870.1	Geobacter bemidjiensis	Geobacter	Geobacteraceae	Desulfuromonadales	Deltaproteobacteria	Proteobacteria	Bacteria
YP_002138052.1	Geobacter bemidjiensis	Geobacter	Geobacteraceae	Desulfuromonadales	Deltaproteobacteria	Proteobacteria	Bacteria
YP_002136948.1	Geobacter bemidjiensis	Geobacter	Geobacteraceae	Desulfuromonadales	Deltaproteobacteria	Proteobacteria	Bacteria
YP_002140689.1	Geobacter bemidjiensis	Geobacter	Geobacteraceae	Desulfuromonadales	Deltaproteobacteria	Proteobacteria	Bacteria
YP_002138063.1	Geobacter bemidjiensis	Geobacter	Geobacteraceae	Desulfuromonadales	Deltaproteobacteria	Proteobacteria	Bacteria
YP_383219.1	Geobacter metallireducens	Geobacter	Geobacteraceae	Desulfuromonadales	Deltaproteobacteria	Proteobacteria	Bacteria
YP_386423.1	Geobacter metallireducens	Geobacter	Geobacteraceae	Desulfuromonadales	Deltaproteobacteria	Proteobacteria	Bacteria
ZP_03025377.1	Geobacter sp. M21	Geobacter	Geobacteraceae	Desulfuromonadales	Deltaproteobacteria	Proteobacteria	Bacteria
ZP_03025828.1	Geobacter sp. M21	Geobacter	Geobacteraceae	Desulfuromonadales	Deltaproteobacteria	Proteobacteria	Bacteria
ZP_03025602.1	Geobacter sp. M21	Geobacter	Geobacteraceae	Desulfuromonadales	Deltaproteobacteria	Proteobacteria	Bacteria
ZP_03024466.1	Geobacter sp. M21	Geobacter	Geobacteraceae	Desulfuromonadales	Deltaproteobacteria	Proteobacteria	Bacteria
NP_951280.1	Geobacter sulfurreducens	Geobacter	Geobacteraceae	Desulfuromonadales	Deltaproteobacteria	Proteobacteria	Bacteria
YP_001229197.1	Geobacter uraniireducens	Geobacter	Geobacteraceae	Desulfuromonadales	Deltaproteobacteria	Proteobacteria	Bacteria
NP_925109.1	Gloeobacter violaceus	Gloeobacter		Gloeobacterales	Gloeobacteria	Cyanobacteria	Bacteria
NP_923686.1	Gloeobacter violaceus	Gloeobacter		Gloeobacterales	Gloeobacteria	Cyanobacteria	Bacteria

Accession	species	genus	order	classe	family	phylum	domain
YP_001602839.1	Gluconacetobacter diazotrophicus	Gluconacetobacter	Acetobacteraceae	Rhodospirillales	Alphaproteobacteria	Proteobacteria	Bacteria
YP_002275232.1	Gluconacetobacter diazotrophicus	Gluconacetobacter	Acetobacteraceae	Rhodospirillales	Alphaproteobacteria	Proteobacteria	Bacteria
YP_002274670.1	Gluconacetobacter diazotrophicus	Gluconacetobacter	Acetobacteraceae	Rhodospirillales	Alphaproteobacteria	Proteobacteria	Bacteria
YP_001602279.1	Gluconacetobacter diazotrophicus	Gluconacetobacter	Acetobacteraceae	Rhodospirillales	Alphaproteobacteria	Proteobacteria	Bacteria
YP_192306.1	Gluconobacter oxydans	Gluconobacter	Acetobacteraceae	Rhodospirillales	Alphaproteobacteria	Proteobacteria	Bacteria
NP_954783.1	Gordonia westfalica	Gordonia	Gordoniaceae	Actinomycetales	Actinobacteria (class)	Actinobacteria	Bacteria
YP_860231.1	Gramella forsetii	Gramella	Flavobacteriaceae	Flavobacteriales	Flavobacteria	Bacteroidetes	Bacteria
YP_861474.1	Gramella forsetii	Gramella	Flavobacteriaceae	Flavobacteriales	Flavobacteria	Bacteroidetes	Bacteria
YP_861439.1	Gramella forsetii	Gramella	Flavobacteriaceae	Flavobacteriales	Flavobacteria	Bacteroidetes	Bacteria
YP_746079.1	Granulibacter bethesdensis	Granulibacter	Acetobacteraceae	Rhodospirillales	Alphaproteobacteria	Proteobacteria	Bacteria
YP_745096.1	Granulibacter bethesdensis	Granulibacter	Acetobacteraceae	Rhodospirillales	Alphaproteobacteria	Proteobacteria	Bacteria
YP_431395.1	Hahella chejuensis	Hahella	Hahellaceae	Oceanospirillales	Gammaproteobacteria	Proteobacteria	Bacteria
YP_433526.1	Hahella chejuensis	Hahella	Hahellaceae	Oceanospirillales	Gammaproteobacteria	Proteobacteria	Bacteria

YP_432036.1	Hahella chejuensis	Hahella	Hahellaceae	Oceanospirillales	Gammaproteobacteria	Proteobacteria	Bacteria
YP_435539.1	Hahella chejuensis	Hahella	Hahellaceae	Oceanospirillales	Gammaproteobacteria	Proteobacteria	Bacteria
YP_135922.1	Haloarcula marismortui	Haloarcula	Halobacteriaceae	Halobacteriales	Halobacteria	Euryarchaeota	Archaea
YP_135564.1	Haloarcula marismortui	Haloarcula	Halobacteriaceae	Halobacteriales	Halobacteria	Euryarchaeota	Archaea
YP_136841.1	Haloarcula marismortui	Haloarcula	Halobacteriaceae	Halobacteriales	Halobacteria	Euryarchaeota	Archaea
YP_135811.1	Haloarcula marismortui	Haloarcula	Halobacteriaceae	Halobacteriales	Halobacteria	Euryarchaeota	Archaea
YP_136806.1	Haloarcula marismortui	Haloarcula	Halobacteriaceae	Halobacteriales	Halobacteria	Euryarchaeota	Archaea
YP_001688771.1	Halobacterium salinarum	Halobacterium	Halobacteriaceae	Halobacteriales	Halobacteria	Euryarchaeota	Archaea
NP_279674.1	Halobacterium salinarum	Halobacterium	Halobacteriaceae	Halobacteriales	Halobacteria	Euryarchaeota	Archaea
YP_001689972.1	Halobacterium salinarum	Halobacterium	Halobacteriaceae	Halobacteriales	Halobacteria	Euryarchaeota	Archaea
NP_444237.1	Halobacterium salinarum	Halobacterium	Halobacteriaceae	Halobacteriales	Halobacteria	Euryarchaeota	Archaea
AAK14378.1	Haloferax volcanii	Haloferax	Halobacteriaceae	Halobacteriales	Halobacteria	Euryarchaeota	Archaea
AAK14377.1	Haloferax volcanii	Haloferax	Halobacteriaceae	Halobacteriales	Halobacteria	Euryarchaeota	Archaea
BAA24701.1	Halomonas halodenitrificans	Halomonas	Halomonadaceae	Oceanospirillales	Gammaproteobacteria	Proteobacteria	Bacteria
AAY40171.1	Halomonas maura	Halomonas	Halomonadaceae	Oceanospirillales	Gammaproteobacteria	Proteobacteria	Bacteria
YP_657381.1	Haloquadratum walsbyi	Haloquadratum	Halobacteriaceae	Halobacteriales	Halobacteria	Euryarchaeota	Archaea
ZP_02014739.1	Halorubrum lacusprofundi	Halorubrum	Halobacteriaceae	Halobacteriales	Halobacteria	Euryarchaeota	Archaea
ZP_02015245.1	Halorubrum lacusprofundi	Halorubrum	Halobacteriaceae	Halobacteriales	Halobacteria	Euryarchaeota	Archaea
YP_664165.1	Helicobacter acinonychis	Helicobacter	Helicobacteraceae	Campylobacterales	Epsilonproteobacteria	Proteobacteria	Bacteria
NP_860787.1	Helicobacter hepaticus	Helicobacter	Helicobacteraceae	Campylobacterales	Epsilonproteobacteria	Proteobacteria	Bacteria
YP_002265766.1	Helicobacter pylori	Helicobacter	Helicobacteraceae	Campylobacterales	Epsilonproteobacteria	Proteobacteria	Bacteria
YP_002300783.1	Helicobacter pylori	Helicobacter	Helicobacteraceae	Campylobacterales	Epsilonproteobacteria	Proteobacteria	Bacteria
YP_626883.1	Helicobacter pylori	Helicobacter	Helicobacteraceae	Campylobacterales	Epsilonproteobacteria	Proteobacteria	Bacteria
NP_206943.1	Helicobacter pylori	Helicobacter	Helicobacteraceae	Campylobacterales	Epsilonproteobacteria	Proteobacteria	Bacteria
YP_001909635.1	Helicobacter pylori	Helicobacter	Helicobacteraceae	Campylobacterales	Epsilonproteobacteria	Proteobacteria	Bacteria
BAA33526.1	Helicobacter pylori	Helicobacter	Helicobacteraceae	Campylobacterales	Epsilonproteobacteria	Proteobacteria	Bacteria
NP_222853.1	Helicobacter pylori	Helicobacter	Helicobacteraceae	Campylobacterales	Epsilonproteobacteria	Proteobacteria	Bacteria
YP_001101152.1	Herminiimonas arsenicoxydans	Herminiimonas	Oxalobacteraceae	Burkholderiales	Betaproteobacteria	Proteobacteria	Bacteria
YP_001099422.1	Herminiimonas arsenicoxydans	Herminiimonas	Oxalobacteraceae	Burkholderiales	Betaproteobacteria	Proteobacteria	Bacteria
YP_001100944.1	Herminiimonas arsenicoxydans	Herminiimonas	Oxalobacteraceae	Burkholderiales	Betaproteobacteria	Proteobacteria	Bacteria
CAL61802.2	Herminiimonas arsenicoxydans	Herminiimonas	Oxalobacteraceae	Burkholderiales	Betaproteobacteria	Proteobacteria	Bacteria
YP_001099927.1	Herminiimonas arsenicoxydans	Herminiimonas	Oxalobacteraceae	Burkholderiales	Betaproteobacteria	Proteobacteria	Bacteria

Accession	species	genus	order	classe	family	phylum	domain
YP_001099949.1	Herminiimonas arsenicoxydans	Herminiimonas	Oxalobacteraceae	Burkholderiales	Betaproteobacteria	Proteobacteria	Bacteria
YP_001098413.1	Herminiimonas arsenicoxydans	Herminiimonas	Oxalobacteraceae	Burkholderiales	Betaproteobacteria	Proteobacteria	Bacteria
YP_001542885.1	Herpetosiphon aurantiacus	Herpetosiphon	Herpetosiphonaceae	Herpetosiphonales	Chloroflexi (class)	Chloroflexi	Bacteria
ZP_02167414.1	Hoeflea phototrophica	Hoeflea	Phyllobacteriaceae	Rhizobiales	Alphaproteobacteria	Proteobacteria	Bacteria
ZP_02167623.1	Hoeflea phototrophica	Hoeflea	Phyllobacteriaceae	Rhizobiales	Alphaproteobacteria	Proteobacteria	Bacteria
ZP_02176553.1	Hydrogenivirga sp. 128-5-R1-1	Hydrogenivirga	Aquificaceae	Aquificales	Aquificae (class)	Aquificae	Bacteria

ZP_02177583.1	Hydrogenivirga sp. 128-5-R1-1	Hydrogenivirga	Aquificaceae	Aquificales	Aquificae (class)	Aquificae	Bacteria
ZP_02176982.1	Hydrogenivirga sp. 128-5-R1-1	Hydrogenivirga	Aquificaceae	Aquificales	Aquificae (class)	Aquificae	Bacteria
BAE79481.1	Hydrogenobacter thermophilus	Hydrogenobacter	Aquificaceae	Aquificales	Aquificae (class)	Aquificae	Bacteria
YP_002121488.1	Hydrogenobaculum sp. Y04AAS1	Hydrogenobaculum	Aquificaceae	Aquificales	Aquificae (class)	Aquificae	Bacteria
YP_002121019.1	Hydrogenobaculum sp. Y04AAS1	Hydrogenobaculum	Aquificaceae	Aquificales	Aquificae (class)	Aquificae	Bacteria
YP_759761.1	Hyphomonas neptunium	Hyphomonas	Hyphomonadaceae	Rhodobacterales	Alphaproteobacteria	Proteobacteria	Bacteria
YP_759950.1	Hyphomonas neptunium	Hyphomonas	Hyphomonadaceae	Rhodobacterales	Alphaproteobacteria	Proteobacteria	Bacteria
ZP_01041859.1	Idiomarina baltica	Idiomarina	Idiomarinaceae	Alteromonadales	Gammaproteobacteria	Proteobacteria	Bacteria
ZP_01043949.1	Idiomarina baltica	Idiomarina	Idiomarinaceae	Alteromonadales	Gammaproteobacteria	Proteobacteria	Bacteria
YP_154651.1	Idiomarina loihiensis	Idiomarina	Idiomarinaceae	Alteromonadales	Gammaproteobacteria	Proteobacteria	Bacteria
YP_155686.1	Idiomarina loihiensis	Idiomarina	Idiomarinaceae	Alteromonadales	Gammaproteobacteria	Proteobacteria	Bacteria
YP_154578.1	Idiomarina loihiensis	Idiomarina	Idiomarinaceae	Alteromonadales	Gammaproteobacteria	Proteobacteria	Bacteria
YP_509192.1	Jannaschia sp. CCS1	Jannaschia	Rhodobacteraceae	Rhodobacterales	Alphaproteobacteria	Proteobacteria	Bacteria
YP_511799.1	Jannaschia sp. CCS1	Jannaschia	Rhodobacteraceae	Rhodobacterales	Alphaproteobacteria	Proteobacteria	Bacteria
YP_001354841.1	Janthinobacterium sp. Marseille	Janthinobacterium	Oxalobacteraceae	Burkholderiales	Betaproteobacteria	Proteobacteria	Bacteria
YP_001354329.1	Janthinobacterium sp. Marseille	Janthinobacterium	Oxalobacteraceae	Burkholderiales	Betaproteobacteria	Proteobacteria	Bacteria
YP_001353321.1	Janthinobacterium sp. Marseille	Janthinobacterium	Oxalobacteraceae	Burkholderiales	Betaproteobacteria	Proteobacteria	Bacteria
YP_001362986.1	Kineococcus radiotolerans	Kineococcus	Kineosporiaceae	Actinomycetales	Actinobacteria (class)	Actinobacteria	Bacteria
YP_001334075.1	Klebsiella pneumoniae	Klebsiella	Enterobacteriaceae	Enterobacteriales	Gammaproteobacteria	Proteobacteria	Bacteria
YP_002240090.1	Klebsiella pneumoniae	Klebsiella	Enterobacteriaceae	Enterobacteriales	Gammaproteobacteria	Proteobacteria	Bacteria
YP_001855138.1	Kocuria rhizophila	Kocuria	Micrococcaceae	Actinomycetales	Actinobacteria (class)	Actinobacteria	Bacteria
ZP_02161004.1	Kordia algicida	Kordia	Flavobacteriaceae	Flavobacteriales	Flavobacteria	Bacteroidetes	Bacteria
ZP_01548128.1	Labrenzia aggregata	Labrenzia	Rhodobacteraceae	Rhodobacterales	Alphaproteobacteria	Proteobacteria	Bacteria
ZP_01548610.1	Labrenzia aggregata	Labrenzia	Rhodobacteraceae	Rhodobacterales	Alphaproteobacteria	Proteobacteria	Bacteria
ZP_01549668.1	Labrenzia aggregata	Labrenzia	Rhodobacteraceae	Rhodobacterales	Alphaproteobacteria	Proteobacteria	Bacteria
YP_001844459.1	Lactobacillus fermentum	Lactobacillus	Lactobacillaceae	Lactobacillales	Bacilli	Firmicutes	Bacteria
ZP_01059311.1	Leeuwenhoekiella blandensis	Leeuwenhoekiella	Flavobacteriaceae	Flavobacteriales	Flavobacteria	Bacteroidetes	Bacteria
ZP_01060293.1	Leeuwenhoekiella blandensis	Leeuwenhoekiella	Flavobacteriaceae	Flavobacteriales	Flavobacteria	Bacteroidetes	Bacteria
YP_128137.1	Legionella pneumophila	Legionella	Legionellaceae	Legionellales	Gammaproteobacteria	Proteobacteria	Bacteria
YP_096890.1	Legionella pneumophila	Legionella	Legionellaceae	Legionellales	Gammaproteobacteria	Proteobacteria	Bacteria
YP_125263.1	Legionella pneumophila	Legionella	Legionellaceae	Legionellales	Gammaproteobacteria	Proteobacteria	Bacteria
YP_001252428.1	Legionella pneumophila	Legionella	Legionellaceae	Legionellales	Gammaproteobacteria	Proteobacteria	Bacteria
YP_122635.1	Legionella pneumophila	Legionella	Legionellaceae	Legionellales	Gammaproteobacteria	Proteobacteria	Bacteria
YP_127497.1	Legionella pneumophila	Legionella	Legionellaceae	Legionellales	Gammaproteobacteria	Proteobacteria	Bacteria
YP_124501.1	Legionella pneumophila	Legionella	Legionellaceae	Legionellales	Gammaproteobacteria	Proteobacteria	Bacteria
YP_096248.1	Legionella pneumophila	Legionella	Legionellaceae	Legionellales	Gammaproteobacteria	Proteobacteria	Bacteria
YP_001250986.1	Legionella pneumophila	Legionella	Legionellaceae	Legionellales	Gammaproteobacteria	Proteobacteria	Bacteria
YP_062417.1	Leifsonia xyli	Leifsonia	Microbacteriaceae	Actinomycetales	Actinobacteria (class)	Actinobacteria	Bacteria
ZP_01877020.1	Lentisphaera araneosa	Lentisphaera	Lentisphaeraceae	Lentisphaerales		Lentisphaerae	Bacteria

Accession	species	genus	order	classe	family	phylum	domain
YP_001963663.1	Leptospira biflexa	Leptospira	Leptospiraceae	Spirochaetales	Spirochaetes (class)	Spirochaetes	Bacteria
YP_001840037.1	Leptospira biflexa	Leptospira	Leptospiraceae	Spirochaetales	Spirochaetes (class)	Spirochaetes	Bacteria
YP_001964466.1	Leptospira biflexa	Leptospira	Leptospiraceae	Spirochaetales	Spirochaetes (class)	Spirochaetes	Bacteria
YP_001964801.1	Leptospira biflexa	Leptospira	Leptospiraceae	Spirochaetales	Spirochaetes (class)	Spirochaetes	Bacteria
ABZ95888.1	Leptospira biflexa	Leptospira	Leptospiraceae	Spirochaetales	Spirochaetes (class)	Spirochaetes	Bacteria
YP_001963907.1	Leptospira biflexa	Leptospira	Leptospiraceae	Spirochaetales	Spirochaetes (class)	Spirochaetes	Bacteria
YP_001840298.1	Leptospira biflexa	Leptospira	Leptospiraceae	Spirochaetales	Spirochaetes (class)	Spirochaetes	Bacteria
YP_796822.1	Leptospira borgpetersenii	Leptospira	Leptospiraceae	Spirochaetales	Spirochaetes (class)	Spirochaetes	Bacteria
YP_801959.1	Leptospira borgpetersenii	Leptospira	Leptospiraceae	Spirochaetales	Spirochaetes (class)	Spirochaetes	Bacteria
YP_800805.1	Leptospira borgpetersenii	Leptospira	Leptospiraceae	Spirochaetales	Spirochaetes (class)	Spirochaetes	Bacteria
YP_798081.1	Leptospira borgpetersenii	Leptospira	Leptospiraceae	Spirochaetales	Spirochaetes (class)	Spirochaetes	Bacteria
YP_000200.1	Leptospira interrogans	Leptospira	Leptospiraceae	Spirochaetales	Spirochaetes (class)	Spirochaetes	Bacteria
NP_710424.1	Leptospira interrogans	Leptospira	Leptospiraceae	Spirochaetales	Spirochaetes (class)	Spirochaetes	Bacteria
EAY56843.1	Leptospirillum ferriphilum	Leptospirillum	Nitrospiraceae	Nitrospirales	Nitrospira (class)	Nitrospirae	Bacteria
YP_001792838.1	Leptothrix cholodnii	Leptothrix		Burkholderiales	Betaproteobacteria	Proteobacteria	Bacteria
YP_001789293.1	Leptothrix cholodnii	Leptothrix		Burkholderiales	Betaproteobacteria	Proteobacteria	Bacteria
YP_001791809.1	Leptothrix cholodnii	Leptothrix		Burkholderiales	Betaproteobacteria	Proteobacteria	Bacteria
YP_001790501.1	Leptothrix cholodnii	Leptothrix		Burkholderiales	Betaproteobacteria	Proteobacteria	Bacteria
ZP_01913894.1	Limnobacter sp. MED105	Limnobacter	Burkholderiaceae	Burkholderiales	Betaproteobacteria	Proteobacteria	Bacteria
ZP_01915177.1	Limnobacter sp. MED105	Limnobacter	Burkholderiaceae	Burkholderiales	Betaproteobacteria	Proteobacteria	Bacteria
NP_469361.1	Listeria innocua	Listeria	Listeriaceae	Bacillales	Bacilli	Firmicutes	Bacteria
YP_002351598.1	Listeria monocytogenes	Listeria	Listeriaceae	Bacillales	Bacilli	Firmicutes	Bacteria
YP_012629.1	Listeria monocytogenes	Listeria	Listeriaceae	Bacillales	Bacilli	Firmicutes	Bacteria
NP_463547.1	Listeria monocytogenes	Listeria	Listeriaceae	Bacillales	Bacilli	Firmicutes	Bacteria
YP_848218.1	Listeria welshimeri	Listeria	Listeriaceae	Bacillales	Bacilli	Firmicutes	Bacteria
ZP_01004262.1	Loktanella vestfoldensis	Loktanella	Rhodobacteraceae	Rhodobacterales	Alphaproteobacteria	Proteobacteria	Bacteria
ZP_01004045.1	Loktanella vestfoldensis	Loktanella	Rhodobacteraceae	Rhodobacterales	Alphaproteobacteria	Proteobacteria	Bacteria
ZP_01623743.1	Lyngbya sp. PCC 8106	Lyngbya		Oscillatoriales		Cyanobacteria	Bacteria
ZP_01621649.1	Lyngbya sp. PCC 8106	Lyngbya		Oscillatoriales		Cyanobacteria	Bacteria
YP_001697134.1	Lysinibacillus sphaericus	Lysinibacillus	Planococcaceae	Bacillales	Bacilli	Firmicutes	Bacteria
YP_001698546.1	Lysinibacillus sphaericus	Lysinibacillus	Planococcaceae	Bacillales	Bacilli	Firmicutes	Bacteria
YP_001699583.1	Lysinibacillus sphaericus	Lysinibacillus	Planococcaceae	Bacillales	Bacilli	Firmicutes	Bacteria
YP_866259.1	Magnetococcus sp. MC-1	Magnetococcus				Proteobacteria	Bacteria
YP_864053.1	Magnetococcus sp. MC-1	Magnetococcus				Proteobacteria	Bacteria
CAM74645.1	Magnetospirillum gryphiswaldense	Magnetospirillum	Rhodospirillaceae	Rhodospirillales	Alphaproteobacteria	Proteobacteria	Bacteria
CAM77358.1	Magnetospirillum gryphiswaldense	Magnetospirillum	Rhodospirillaceae	Rhodospirillales	Alphaproteobacteria	Proteobacteria	Bacteria
CAM75374.1	Magnetospirillum gryphiswaldense	Magnetospirillum	Rhodospirillaceae	Rhodospirillales	Alphaproteobacteria	Proteobacteria	Bacteria
YP_421532.1	Magnetospirillum magneticum	Magnetospirillum	Rhodospirillaceae	Rhodospirillales	Alphaproteobacteria	Proteobacteria	Bacteria
YP_422307.1	Magnetospirillum magneticum	Magnetospirillum	Rhodospirillaceae	Rhodospirillales	Alphaproteobacteria	Proteobacteria	Bacteria
ZP_00050953.2	Magnetospirillum magnetotacticum	Magnetospirillum	Rhodospirillaceae	Rhodospirillales	Alphaproteobacteria	Proteobacteria	Bacteria
ZP_00054051.1	Magnetospirillum magnetotacticum	Magnetospirillum	Rhodospirillaceae	Rhodospirillales	Alphaproteobacteria	Proteobacteria	Bacteria

ZP_00055725.1	Magnetospirillum magnetotacticum	Magnetospirillum	Rhodospirillaceae	Rhodospirillales	Alphaproteobacteria	Proteobacteria	Bacteria
YP_087486.1	Mannheimia succiniciproducens	Mannheimia	Pasteurellaceae	Pasteurellales	Gammaproteobacteria	Proteobacteria	Bacteria
YP_757463.1	Maricaulis maris	Maricaulis	Hyphomonadaceae	Rhodobacterales	Alphaproteobacteria	Proteobacteria	Bacteria
YP_756380.1	Maricaulis maris	Maricaulis	Hyphomonadaceae	Rhodobacterales	Alphaproteobacteria	Proteobacteria	Bacteria

Accession	species	genus	order	classe	family	phylum	domain
ZP_01131187.1	marine actinobacterium PHSC20C1				Actinobacteria (class)	Actinobacteria	Bacteria
ZP_01615637.1	marine gamma proteobacterium HTCC2143				Gammaproteobacteria	Proteobacteria	Bacteria
ZP_01617056.1	marine gamma proteobacterium HTCC2143				Gammaproteobacteria	Proteobacteria	Bacteria
ZP_01616294.1	marine gamma proteobacterium HTCC2143				Gammaproteobacteria	Proteobacteria	Bacteria
ZP_01893361.1	Marinobacter algicola	Marinobacter	Alteromonadaceae	Alteromonadales	Gammaproteobacteria	Proteobacteria	Bacteria
ZP_01894120.1	Marinobacter algicola	Marinobacter	Alteromonadaceae	Alteromonadales	Gammaproteobacteria	Proteobacteria	Bacteria
YP_957357.1	Marinobacter hydrocarbonoclasticus	Marinobacter	Alteromonadaceae	Alteromonadales	Gammaproteobacteria	Proteobacteria	Bacteria
YP_959053.1	Marinobacter hydrocarbonoclasticus	Marinobacter	Alteromonadaceae	Alteromonadales	Gammaproteobacteria	Proteobacteria	Bacteria
YP_960376.1	Marinobacter hydrocarbonoclasticus	Marinobacter	Alteromonadaceae	Alteromonadales	Gammaproteobacteria	Proteobacteria	Bacteria
ZP_01736607.1	Marinobacter sp. ELB17	Marinobacter	Alteromonadaceae	Alteromonadales	Gammaproteobacteria	Proteobacteria	Bacteria
ZP_01738263.1	Marinobacter sp. ELB17	Marinobacter	Alteromonadaceae	Alteromonadales	Gammaproteobacteria	Proteobacteria	Bacteria
ZP_01737388.1	Marinobacter sp. ELB17	Marinobacter	Alteromonadaceae	Alteromonadales	Gammaproteobacteria	Proteobacteria	Bacteria
ZP_01736782.1	Marinobacter sp. ELB17	Marinobacter	Alteromonadaceae	Alteromonadales	Gammaproteobacteria	Proteobacteria	Bacteria
ZP_01077262.1	Marinomonas sp. MED121	Marinomonas	Oceanospirillaceae	Oceanospirillales	Gammaproteobacteria	Proteobacteria	Bacteria
ZP_01075802.1	Marinomonas sp. MED121	Marinomonas	Oceanospirillaceae	Oceanospirillales	Gammaproteobacteria	Proteobacteria	Bacteria
YP_001338967.1	Marinomonas sp. MWYL1	Marinomonas	Oceanospirillaceae	Oceanospirillales	Gammaproteobacteria	Proteobacteria	Bacteria
ZP_01453733.1	Mariprofundus ferrooxydans	Mariprofundus	Mariprofundaceae	Mariprofundales	Zetaproteobacteria	Proteobacteria	Bacteria
NP_107796.1	Mesorhizobium loti	Mesorhizobium	Phyllobacteriaceae	Rhizobiales	Alphaproteobacteria	Proteobacteria	Bacteria
NP_102720.1	Mesorhizobium loti	Mesorhizobium	Phyllobacteriaceae	Rhizobiales	Alphaproteobacteria	Proteobacteria	Bacteria
NP_107274.1	Mesorhizobium loti	Mesorhizobium	Phyllobacteriaceae	Rhizobiales	Alphaproteobacteria	Proteobacteria	Bacteria
NP_109481.1	Mesorhizobium loti	Mesorhizobium	Phyllobacteriaceae	Rhizobiales	Alphaproteobacteria	Proteobacteria	Bacteria
NP_106928.1	Mesorhizobium loti	Mesorhizobium	Phyllobacteriaceae	Rhizobiales	Alphaproteobacteria	Proteobacteria	Bacteria
CAD31422.1	Mesorhizobium loti	Mesorhizobium	Phyllobacteriaceae	Rhizobiales	Alphaproteobacteria	Proteobacteria	Bacteria
NP_107099.1	Mesorhizobium loti	Mesorhizobium	Phyllobacteriaceae	Rhizobiales	Alphaproteobacteria	Proteobacteria	Bacteria
YP_673307.1	Mesorhizobium sp. BNC1	Mesorhizobium	Phyllobacteriaceae	Rhizobiales	Alphaproteobacteria	Proteobacteria	Bacteria
YP_675024.1	Mesorhizobium sp. BNC1	Mesorhizobium	Phyllobacteriaceae	Rhizobiales	Alphaproteobacteria	Proteobacteria	Bacteria
YP_676071.1	Mesorhizobium sp. BNC1	Mesorhizobium	Phyllobacteriaceae	Rhizobiales	Alphaproteobacteria	Proteobacteria	Bacteria
YP_675332.1	Mesorhizobium sp. BNC1	Mesorhizobium	Phyllobacteriaceae	Rhizobiales	Alphaproteobacteria	Proteobacteria	Bacteria
YP_674805.1	Mesorhizobium sp. BNC1	Mesorhizobium	Phyllobacteriaceae	Rhizobiales	Alphaproteobacteria	Proteobacteria	Bacteria
YP_001190425.1	Metallosphaera sedula	Metallosphaera	Sulfolobaceae	Sulfolobales	Thermoprotei	Crenarchaeota	Archaea
YP_001192096.1	Metallosphaera sedula	Metallosphaera	Sulfolobaceae	Sulfolobales	Thermoprotei	Crenarchaeota	Archaea
YP_001190669.1	Metallosphaera sedula	Metallosphaera	Sulfolobaceae	Sulfolobales	Thermoprotei	Crenarchaeota	Archaea
YP_001190585.1	Metallosphaera sedula	Metallosphaera	Sulfolobaceae	Sulfolobales	Thermoprotei	Crenarchaeota	Archaea

YP_001190586.1	Metallosphaera sedula	Metallosphaera	Sulfolobaceae	Sulfolobales	Thermoprotei	Crenarchaeota	Archaea
YP_001190391.1	Metallosphaera sedula	Metallosphaera	Sulfolobaceae	Sulfolobales	Thermoprotei	Crenarchaeota	Archaea
YP_001940602.1	Methylacidiphilum infernorum	Methylacidiphilum				Verrucomicrobia	Bacteria
YP_001938924.1	Methylacidiphilum infernorum	Methylacidiphilum				Verrucomicrobia	Bacteria
YP_001939709.1	Methylacidiphilum infernorum	Methylacidiphilum				Verrucomicrobia	Bacteria
YP_001938701.1	Methylacidiphilum infernorum	Methylacidiphilum				Verrucomicrobia	Bacteria
ABX56670.1	Methylacidiphilum infernorum	Methylacidiphilum				Verrucomicrobia	Bacteria
YP_001022368.1	Methylibium petroleiphilum	Methylibium		Burkholderiales	Betaproteobacteria	Proteobacteria	Bacteria
YP_001020937.1	Methylibium petroleiphilum	Methylibium		Burkholderiales	Betaproteobacteria	Proteobacteria	Bacteria
YP_001021668.1	Methylibium petroleiphilum	Methylibium		Burkholderiales	Betaproteobacteria	Proteobacteria	Bacteria
YP_545403.1	Methylobacillus flagellatus	Methylobacillus	Methylophilaceae	Methylophilales	Betaproteobacteria	Proteobacteria	Bacteria
YP_545557.1	Methylobacillus flagellatus	Methylobacillus	Methylophilaceae	Methylophilales	Betaproteobacteria	Proteobacteria	Bacteria

Accession	species	genus	order	classe	family	phylum	domain
YP_544740.1	Methylobacillus flagellatus	Methylobacillus	Methylophilaceae	Methylophilales	Betaproteobacteria	Proteobacteria	Bacteria
YP_545543.1	Methylobacillus flagellatus	Methylobacillus	Methylophilaceae	Methylophilales	Betaproteobacteria	Proteobacteria	Bacteria
ZP_02059811.1	Methylobacterium chloromethanicum	Methylobacterium	Methylobacteriaceae	Rhizobiales	Alphaproteobacteria	Proteobacteria	Bacteria
YP_002422334.1	Methylobacterium chloromethanicum	Methylobacterium	Methylobacteriaceae	Rhizobiales	Alphaproteobacteria	Proteobacteria	Bacteria
YP_002423407.1	Methylobacterium chloromethanicum	Methylobacterium	Methylobacteriaceae	Rhizobiales	Alphaproteobacteria	Proteobacteria	Bacteria
ZP_02057820.1	Methylobacterium chloromethanicum	Methylobacterium	Methylobacteriaceae	Rhizobiales	Alphaproteobacteria	Proteobacteria	Bacteria
YP_002419042.1	Methylobacterium chloromethanicum	Methylobacterium	Methylobacteriaceae	Rhizobiales	Alphaproteobacteria	Proteobacteria	Bacteria
ZP_02056159.1	Methylobacterium chloromethanicum	Methylobacterium	Methylobacteriaceae	Rhizobiales	Alphaproteobacteria	Proteobacteria	Bacteria
YP_001640720.1	Methylobacterium extorquens	Methylobacterium	Methylobacteriaceae	Rhizobiales	Alphaproteobacteria	Proteobacteria	Bacteria
YP_001641776.1	Methylobacterium extorquens	Methylobacterium	Methylobacteriaceae	Rhizobiales	Alphaproteobacteria	Proteobacteria	Bacteria
YP_001637594.1	Methylobacterium extorquens	Methylobacterium	Methylobacteriaceae	Rhizobiales	Alphaproteobacteria	Proteobacteria	Bacteria
YP_001637720.1	Methylobacterium extorquens	Methylobacterium	Methylobacteriaceae	Rhizobiales	Alphaproteobacteria	Proteobacteria	Bacteria
ZP_02123893.1	Methylobacterium nodulans	Methylobacterium	Methylobacteriaceae	Rhizobiales	Alphaproteobacteria	Proteobacteria	Bacteria
ZP_02118651.1	Methylobacterium nodulans	Methylobacterium	Methylobacteriaceae	Rhizobiales	Alphaproteobacteria	Proteobacteria	Bacteria
ZP_02124708.1	Methylobacterium nodulans	Methylobacterium	Methylobacteriaceae	Rhizobiales	Alphaproteobacteria	Proteobacteria	Bacteria
ZP_02118662.1	Methylobacterium nodulans	Methylobacterium	Methylobacteriaceae	Rhizobiales	Alphaproteobacteria	Proteobacteria	Bacteria
ZP_02117357.1	Methylobacterium nodulans	Methylobacterium	Methylobacteriaceae	Rhizobiales	Alphaproteobacteria	Proteobacteria	Bacteria
ZP_02124900.1	Methylobacterium nodulans	Methylobacterium	Methylobacteriaceae	Rhizobiales	Alphaproteobacteria	Proteobacteria	Bacteria
YP_001926144.1	Methylobacterium populi	Methylobacterium	Methylobacteriaceae	Rhizobiales	Alphaproteobacteria	Proteobacteria	Bacteria
YP_001927484.1	Methylobacterium populi	Methylobacterium	Methylobacteriaceae	Rhizobiales	Alphaproteobacteria	Proteobacteria	Bacteria
YP_001922756.1	Methylobacterium populi	Methylobacterium	Methylobacteriaceae	Rhizobiales	Alphaproteobacteria	Proteobacteria	Bacteria
YP_001752742.1	Methylobacterium radiotolerans	Methylobacterium	Methylobacteriaceae	Rhizobiales	Alphaproteobacteria	Proteobacteria	Bacteria
YP_001754410.1	Methylobacterium radiotolerans	Methylobacterium	Methylobacteriaceae	Rhizobiales	Alphaproteobacteria	Proteobacteria	Bacteria
YP_001766956.1	Methylobacterium radiotolerans	Methylobacterium	Methylobacteriaceae	Rhizobiales	Alphaproteobacteria	Proteobacteria	Bacteria
YP_001753729.1	Methylobacterium radiotolerans	Methylobacterium	Methylobacteriaceae	Rhizobiales	Alphaproteobacteria	Proteobacteria	Bacteria

YP_001769241.1	Methylobacterium sp. 4-46	Methylobacterium	Methylobacteriaceae	Rhizobiales	Alphaproteobacteria	Proteobacteria	Bacteria
YP_001770700.1	Methylobacterium sp. 4-46	Methylobacterium	Methylobacteriaceae	Rhizobiales	Alphaproteobacteria	Proteobacteria	Bacteria
YP_001772457.1	Methylobacterium sp. 4-46	Methylobacterium	Methylobacteriaceae	Rhizobiales	Alphaproteobacteria	Proteobacteria	Bacteria
YP_001769836.1	Methylobacterium sp. 4-46	Methylobacterium	Methylobacteriaceae	Rhizobiales	Alphaproteobacteria	Proteobacteria	Bacteria
YP_001772537.1	Methylobacterium sp. 4-46	Methylobacterium	Methylobacteriaceae	Rhizobiales	Alphaproteobacteria	Proteobacteria	Bacteria
YP_001770109.1	Methylobacterium sp. 4-46	Methylobacterium	Methylobacteriaceae	Rhizobiales	Alphaproteobacteria	Proteobacteria	Bacteria
YP_002361569.1	Methylocella silvestris	Methylocella	Beijerinckiaceae	Rhizobiales	Alphaproteobacteria	Proteobacteria	Bacteria
ZP_02957295.1	Methylocella silvestris	Methylocella	Beijerinckiaceae	Rhizobiales	Alphaproteobacteria	Proteobacteria	Bacteria
YP_002363645.1	Methylocella silvestris	Methylocella	Beijerinckiaceae	Rhizobiales	Alphaproteobacteria	Proteobacteria	Bacteria
ZP_02956138.1	Methylocella silvestris	Methylocella	Beijerinckiaceae	Rhizobiales	Alphaproteobacteria	Proteobacteria	Bacteria
YP_002363906.1	Methylocella silvestris	Methylocella	Beijerinckiaceae	Rhizobiales	Alphaproteobacteria	Proteobacteria	Bacteria
YP_002361828.1	Methylocella silvestris	Methylocella	Beijerinckiaceae	Rhizobiales	Alphaproteobacteria	Proteobacteria	Bacteria
ZP_02947292.1	Methylocella silvestris	Methylocella	Beijerinckiaceae	Rhizobiales	Alphaproteobacteria	Proteobacteria	Bacteria
YP_113374.1	Methylococcus capsulatus	Methylococcus	Methylococcaceae	Methylococcales	Gammaproteobacteria	Proteobacteria	Bacteria
YP_114811.1	Methylococcus capsulatus	Methylococcus	Methylococcaceae	Methylococcales	Gammaproteobacteria	Proteobacteria	Bacteria
YP_114815.1	Methylococcus capsulatus	Methylococcus	Methylococcaceae	Methylococcales	Gammaproteobacteria	Proteobacteria	Bacteria
ZP_01552610.1	Methylophilales bacterium HTCC2181			Methylophilales	Betaproteobacteria	Proteobacteria	Bacteria
ZP_02944669.1	Micrococcus luteus	Micrococcus	Micrococcaceae	Actinomycetales	Actinobacteria (class)	Actinobacteria	Bacteria
EDX74433.1	Microcoleus chthonoplastes	Microcoleus		Oscillatoriales		Cyanobacteria	Bacteria
YP_001657308.1	Microcystis aeruginosa	Microcystis		Chroococcales		Cyanobacteria	Bacteria

Accession	species	genus	order	classe	family	phylum	domain
ZP_01690072.1	Microscilla marina	Microscilla	Flexibacteraceae	Sphingobacteriales	Sphingobacteria	Bacteroidetes	Bacteria
ZP_01690773.1	Microscilla marina	Microscilla	Flexibacteraceae	Sphingobacteriales	Sphingobacteria	Bacteroidetes	Bacteria
BAA89393.1	Moritella marina	Moritella	Moritellaceae	Alteromonadales	Gammaproteobacteria	Proteobacteria	Bacteria
ZP_01900412.1	Moritella sp. PE36	Moritella	Moritellaceae	Alteromonadales	Gammaproteobacteria	Proteobacteria	Bacteria
ZP_01897896.1	Moritella sp. PE36	Moritella	Moritellaceae	Alteromonadales	Gammaproteobacteria	Proteobacteria	Bacteria
YP_001703352.1	Mycobacterium abscessus	Mycobacterium	Mycobacteriaceae	Actinomycetales	Actinobacteria (class)	Actinobacteria	Bacteria
YP_001701786.1	Mycobacterium abscessus	Mycobacterium	Mycobacteriaceae	Actinomycetales	Actinobacteria (class)	Actinobacteria	Bacteria
YP_001704119.1	Mycobacterium abscessus	Mycobacterium	Mycobacteriaceae	Actinomycetales	Actinobacteria (class)	Actinobacteria	Bacteria
NP_962025.1	Mycobacterium avium	Mycobacterium	Mycobacteriaceae	Actinomycetales	Actinobacteria (class)	Actinobacteria	Bacteria
YP_883070.1	Mycobacterium avium	Mycobacterium	Mycobacteriaceae	Actinomycetales	Actinobacteria (class)	Actinobacteria	Bacteria
NP_962115.1	Mycobacterium avium	Mycobacterium	Mycobacteriaceae	Actinomycetales	Actinobacteria (class)	Actinobacteria	Bacteria
YP_883164.1	Mycobacterium avium	Mycobacterium	Mycobacteriaceae	Actinomycetales	Actinobacteria (class)	Actinobacteria	Bacteria
NP_856714.1	Mycobacterium bovis	Mycobacterium	Mycobacteriaceae	Actinomycetales	Actinobacteria (class)	Actinobacteria	Bacteria
YP_979151.1	Mycobacterium bovis	Mycobacterium	Mycobacteriaceae	Actinomycetales	Actinobacteria (class)	Actinobacteria	Bacteria
YP_001132466.1	Mycobacterium gilvum	Mycobacterium	Mycobacteriaceae	Actinomycetales	Actinobacteria (class)	Actinobacteria	Bacteria
YP_001134083.1	Mycobacterium gilvum	Mycobacterium	Mycobacteriaceae	Actinomycetales	Actinobacteria (class)	Actinobacteria	Bacteria
YP_001136626.1	Mycobacterium gilvum	Mycobacterium	Mycobacteriaceae	Actinomycetales	Actinobacteria (class)	Actinobacteria	Bacteria

YP_001135534.1	Mycobacterium gilvum	Mycobacterium	Mycobacteriaceae	Actinomycetales	Actinobacteria (class)	Actinobacteria	Bacteria
NP_302190.1	Mycobacterium leprae	Mycobacterium	Mycobacteriaceae	Actinomycetales	Actinobacteria (class)	Actinobacteria	Bacteria
YP_001851577.1	Mycobacterium marinum	Mycobacterium	Mycobacteriaceae	Actinomycetales	Actinobacteria (class)	Actinobacteria	Bacteria
YP_001849956.1	Mycobacterium marinum	Mycobacterium	Mycobacteriaceae	Actinomycetales	Actinobacteria (class)	Actinobacteria	Bacteria
YP_888712.1	Mycobacterium smegmatis	Mycobacterium	Mycobacteriaceae	Actinomycetales	Actinobacteria (class)	Actinobacteria	Bacteria
YP_001070112.1	Mycobacterium sp. JLS	Mycobacterium	Mycobacteriaceae	Actinomycetales	Actinobacteria (class)	Actinobacteria	Bacteria
YP_001071718.1	Mycobacterium sp. JLS	Mycobacterium	Mycobacteriaceae	Actinomycetales	Actinobacteria (class)	Actinobacteria	Bacteria
YP_001069828.1	Mycobacterium sp. JLS	Mycobacterium	Mycobacteriaceae	Actinomycetales	Actinobacteria (class)	Actinobacteria	Bacteria
YP_937889.1	Mycobacterium sp. KMS	Mycobacterium	Mycobacteriaceae	Actinomycetales	Actinobacteria (class)	Actinobacteria	Bacteria
YP_939485.1	Mycobacterium sp. KMS	Mycobacterium	Mycobacteriaceae	Actinomycetales	Actinobacteria (class)	Actinobacteria	Bacteria
YP_937591.1	Mycobacterium sp. KMS	Mycobacterium	Mycobacteriaceae	Actinomycetales	Actinobacteria (class)	Actinobacteria	Bacteria
YP_639017.1	Mycobacterium sp. MCS	Mycobacterium	Mycobacteriaceae	Actinomycetales	Actinobacteria (class)	Actinobacteria	Bacteria
YP_640601.1	Mycobacterium sp. MCS	Mycobacterium	Mycobacteriaceae	Actinomycetales	Actinobacteria (class)	Actinobacteria	Bacteria
YP_638736.1	Mycobacterium sp. MCS	Mycobacterium	Mycobacteriaceae	Actinomycetales	Actinobacteria (class)	Actinobacteria	Bacteria
NP_217559.1	Mycobacterium tuberculosis	Mycobacterium	Mycobacteriaceae	Actinomycetales	Actinobacteria (class)	Actinobacteria	Bacteria
YP_001284419.1	Mycobacterium tuberculosis	Mycobacterium	Mycobacteriaceae	Actinomycetales	Actinobacteria (class)	Actinobacteria	Bacteria
YP_001288988.1	Mycobacterium tuberculosis	Mycobacterium	Mycobacteriaceae	Actinomycetales	Actinobacteria (class)	Actinobacteria	Bacteria
NP_337644.1	Mycobacterium tuberculosis	Mycobacterium	Mycobacteriaceae	Actinomycetales	Actinobacteria (class)	Actinobacteria	Bacteria
YP_905358.1	Mycobacterium ulcerans	Mycobacterium	Mycobacteriaceae	Actinomycetales	Actinobacteria (class)	Actinobacteria	Bacteria
YP_905820.1	Mycobacterium ulcerans	Mycobacterium	Mycobacteriaceae	Actinomycetales	Actinobacteria (class)	Actinobacteria	Bacteria
YP_956381.1	Mycobacterium vanbaalenii	Mycobacterium	Mycobacteriaceae	Actinomycetales	Actinobacteria (class)	Actinobacteria	Bacteria
YP_954495.1	Mycobacterium vanbaalenii	Mycobacterium	Mycobacteriaceae	Actinomycetales	Actinobacteria (class)	Actinobacteria	Bacteria
YP_952903.1	Mycobacterium vanbaalenii	Mycobacterium	Mycobacteriaceae	Actinomycetales	Actinobacteria (class)	Actinobacteria	Bacteria
YP_634222.1	Myxococcus xanthus	Myxococcus	Myxococcaceae	Myxococcales	Deltaproteobacteria	Proteobacteria	Bacteria
YP_632048.1	Myxococcus xanthus	Myxococcus	Myxococcaceae	Myxococcales	Deltaproteobacteria	Proteobacteria	Bacteria
YP_633680.1	Myxococcus xanthus	Myxococcus	Myxococcaceae	Myxococcales	Deltaproteobacteria	Proteobacteria	Bacteria
YP_326879.1	Natronomonas pharaonis	Natronomonas	Halobacteriaceae	Halobacteriales	Halobacteria	Euryarchaeota	Archaea
YP_327130.1	Natronomonas pharaonis	Natronomonas	Halobacteriaceae	Halobacteriales	Halobacteria	Euryarchaeota	Archaea

Accession	species	genus	order	classe	family	phylum	domain
YP_002002235.1	Neisseria gonorrhoeae	Neisseria	Neisseriaceae	Neisseriales	Betaproteobacteria	Proteobacteria	Bacteria
YP_208434.1	Neisseria gonorrhoeae	Neisseria	Neisseriaceae	Neisseriales	Betaproteobacteria	Proteobacteria	Bacteria
YP_002002114.1	Neisseria gonorrhoeae	Neisseria	Neisseriaceae	Neisseriales	Betaproteobacteria	Proteobacteria	Bacteria
YP_208344.1	Neisseria gonorrhoeae	Neisseria	Neisseriaceae	Neisseriales	Betaproteobacteria	Proteobacteria	Bacteria
YP_975604.1	Neisseria meningitidis	Neisseria	Neisseriaceae	Neisseriales	Betaproteobacteria	Proteobacteria	Bacteria
NP_274728.1	Neisseria meningitidis	Neisseria	Neisseriaceae	Neisseriales	Betaproteobacteria	Proteobacteria	Bacteria
YP_002343249.1	Neisseria meningitidis	Neisseria	Neisseriaceae	Neisseriales	Betaproteobacteria	Proteobacteria	Bacteria
YP_001599744.1	Neisseria meningitidis	Neisseria	Neisseriaceae	Neisseriales	Betaproteobacteria	Proteobacteria	Bacteria
YP_975519.1	Neisseria meningitidis	Neisseria	Neisseriaceae	Neisseriales	Betaproteobacteria	Proteobacteria	Bacteria

YP_002343169.1	Neisseria meningitidis	Neisseria	Neisseriaceae	Neisseriales	Betaproteobacteria	Proteobacteria	Bacteria
NP_274628.1	Neisseria meningitidis	Neisseria	Neisseriaceae	Neisseriales	Betaproteobacteria	Proteobacteria	Bacteria
YP_001599647.1	Neisseria meningitidis	Neisseria	Neisseriaceae	Neisseriales	Betaproteobacteria	Proteobacteria	Bacteria
YP_506501.1	Neorickettsia sennetsu	Neorickettsia	Anaplasmataceae	Rickettsiales	Alphaproteobacteria	Proteobacteria	Bacteria
ZP_01166403.1	Neptuniibacter caesariensis	Neptuniibacter	Oceanospirillaceae	Oceanospirillales	Gammaproteobacteria	Proteobacteria	Bacteria
ZP_01166126.1	Neptuniibacter caesariensis	Neptuniibacter	Oceanospirillaceae	Oceanospirillales	Gammaproteobacteria	Proteobacteria	Bacteria
YP_001357081.1	Nitratiruptor sp. SB155-2	Nitratiruptor			Epsilonproteobacteria	Proteobacteria	Bacteria
YP_001357257.1	Nitratiruptor sp. SB155-2	Nitratiruptor			Epsilonproteobacteria	Proteobacteria	Bacteria
YP_578651.1	Nitrobacter hamburgensis	Nitrobacter	Bradyrhizobiaceae	Rhizobiales	Alphaproteobacteria	Proteobacteria	Bacteria
YP_575616.1	Nitrobacter hamburgensis	Nitrobacter	Bradyrhizobiaceae	Rhizobiales	Alphaproteobacteria	Proteobacteria	Bacteria
YP_578831.1	Nitrobacter hamburgensis	Nitrobacter	Bradyrhizobiaceae	Rhizobiales	Alphaproteobacteria	Proteobacteria	Bacteria
YP_571634.1	Nitrobacter hamburgensis	Nitrobacter	Bradyrhizobiaceae	Rhizobiales	Alphaproteobacteria	Proteobacteria	Bacteria
YP_578641.1	Nitrobacter hamburgensis	Nitrobacter	Bradyrhizobiaceae	Rhizobiales	Alphaproteobacteria	Proteobacteria	Bacteria
YP_577950.1	Nitrobacter hamburgensis	Nitrobacter	Bradyrhizobiaceae	Rhizobiales	Alphaproteobacteria	Proteobacteria	Bacteria
ZP_01045192.1	Nitrobacter sp. Nb-311A	Nitrobacter	Bradyrhizobiaceae	Rhizobiales	Alphaproteobacteria	Proteobacteria	Bacteria
ZP_01048077.1	Nitrobacter sp. Nb-311A	Nitrobacter	Bradyrhizobiaceae	Rhizobiales	Alphaproteobacteria	Proteobacteria	Bacteria
YP_317379.1	Nitrobacter winogradskyi	Nitrobacter	Bradyrhizobiaceae	Rhizobiales	Alphaproteobacteria	Proteobacteria	Bacteria
YP_316844.1	Nitrobacter winogradskyi	Nitrobacter	Bradyrhizobiaceae	Rhizobiales	Alphaproteobacteria	Proteobacteria	Bacteria
YP_318920.1	Nitrobacter winogradskyi	Nitrobacter	Bradyrhizobiaceae	Rhizobiales	Alphaproteobacteria	Proteobacteria	Bacteria
YP_317387.1	Nitrobacter winogradskyi	Nitrobacter	Bradyrhizobiaceae	Rhizobiales	Alphaproteobacteria	Proteobacteria	Bacteria
ZP_01127177.1	Nitrococcus mobilis	Nitrococcus	Ectothiorhodospiraceae	Chromatiales	Gammaproteobacteria	Proteobacteria	Bacteria
YP_345017.1	Nitrosococcus oceani	Nitrosococcus	Chromatiaceae	Chromatiales	Gammaproteobacteria	Proteobacteria	Bacteria
YP_343768.1	Nitrosococcus oceani	Nitrosococcus	Chromatiaceae	Chromatiales	Gammaproteobacteria	Proteobacteria	Bacteria
YP_343276.1	Nitrosococcus oceani	Nitrosococcus	Chromatiaceae	Chromatiales	Gammaproteobacteria	Proteobacteria	Bacteria
YP_344942.1	Nitrosococcus oceani	Nitrosococcus	Chromatiaceae	Chromatiales	Gammaproteobacteria	Proteobacteria	Bacteria
YP_343846.1	Nitrosococcus oceani	Nitrosococcus	Chromatiaceae	Chromatiales	Gammaproteobacteria	Proteobacteria	Bacteria
NP_841089.1	Nitrosomonas europaea	Nitrosomonas	Nitrosomonadaceae	Nitrosomonadales	Betaproteobacteria	Proteobacteria	Bacteria
NP_840762.1	Nitrosomonas europaea	Nitrosomonas	Nitrosomonadaceae	Nitrosomonadales	Betaproteobacteria	Proteobacteria	Bacteria
NP_842019.1	Nitrosomonas europaea	Nitrosomonas	Nitrosomonadaceae	Nitrosomonadales	Betaproteobacteria	Proteobacteria	Bacteria
YP_748573.1	Nitrosomonas eutropha	Nitrosomonas	Nitrosomonadaceae	Nitrosomonadales	Betaproteobacteria	Proteobacteria	Bacteria
YP_746933.1	Nitrosomonas eutropha	Nitrosomonas	Nitrosomonadaceae	Nitrosomonadales	Betaproteobacteria	Proteobacteria	Bacteria
YP_748072.1	Nitrosomonas eutropha	Nitrosomonas	Nitrosomonadaceae	Nitrosomonadales	Betaproteobacteria	Proteobacteria	Bacteria
YP_747791.1	Nitrosomonas eutropha	Nitrosomonas	Nitrosomonadaceae	Nitrosomonadales	Betaproteobacteria	Proteobacteria	Bacteria
YP_746761.1	Nitrosomonas eutropha	Nitrosomonas	Nitrosomonadaceae	Nitrosomonadales	Betaproteobacteria	Proteobacteria	Bacteria
YP_001581518.1	Nitrosopumilus maritimus	Nitrosopumilus	Nitrosopumilaceae	Nitrosopumilales	Thermoprotei	Crenarchaeota	Archaea
YP_410884.1	Nitrosospira multiformis	Nitrosospira	Nitrosomonadaceae	Nitrosomonadales	Betaproteobacteria	Proteobacteria	Bacteria

Accession	species	genus	order	classe	family	phylum	domain
YP_412465.1	Nitrosospira multiformis	Nitrosospira	Nitrosomonadaceae	Nitrosomonadales	Betaproteobacteria	Proteobacteria	Bacteria

YP_411159.1	Nitrosospira multiformis	Nitrosospira	Nitrosomonadaceae	Nitrosomonadales	Betaproteobacteria	Proteobacteria	Bacteria
YP_413345.1	Nitrosospira multiformis	Nitrosospira	Nitrosomonadaceae	Nitrosomonadales	Betaproteobacteria	Proteobacteria	Bacteria
YP_411950.1	Nitrosospira multiformis	Nitrosospira	Nitrosomonadaceae	Nitrosomonadales	Betaproteobacteria	Proteobacteria	Bacteria
YP_117403.1	Nocardia farcinica	Nocardia	Nocardiaceae	Actinomycetales	Actinobacteria (class)	Actinobacteria	Bacteria
YP_120507.1	Nocardia farcinica	Nocardia	Nocardiaceae	Actinomycetales	Actinobacteria (class)	Actinobacteria	Bacteria
YP_924329.1	Nocardioides sp. JS614	Nocardioides	Nocardioidaceae	Actinomycetales	Actinobacteria (class)	Actinobacteria	Bacteria
YP_919310.1	Nocardioides sp. JS614	Nocardioides	Nocardioidaceae	Actinomycetales	Actinobacteria (class)	Actinobacteria	Bacteria
ZP_01628240.1	Nodularia spumigena	Nodularia	Nostocaceae	Nostocales		Cyanobacteria	Bacteria
ZP_01628371.1	Nodularia spumigena	Nodularia	Nostocaceae	Nostocales		Cyanobacteria	Bacteria
ZP_01628175.1	Nodularia spumigena	Nodularia	Nostocaceae	Nostocales		Cyanobacteria	Bacteria
ZP_00109104.1	Nostoc punctiforme	Nostoc	Nostocaceae	Nostocales		Cyanobacteria	Bacteria
YP_001865393.1	Nostoc punctiforme	Nostoc	Nostocaceae	Nostocales		Cyanobacteria	Bacteria
YP_001866883.1	Nostoc punctiforme	Nostoc	Nostocaceae	Nostocales		Cyanobacteria	Bacteria
YP_001864063.1	Nostoc punctiforme	Nostoc	Nostocaceae	Nostocales		Cyanobacteria	Bacteria
YP_001868782.1	Nostoc punctiforme	Nostoc	Nostocaceae	Nostocales		Cyanobacteria	Bacteria
ZP_00110624.2	Nostoc punctiforme	Nostoc	Nostocaceae	Nostocales		Cyanobacteria	Bacteria
NP_486772.1	Nostoc sp. PCC 7120	Nostoc	Nostocaceae	Nostocales		Cyanobacteria	Bacteria
NP_486555.1	Nostoc sp. PCC 7120	Nostoc	Nostocaceae	Nostocales		Cyanobacteria	Bacteria
NP_484994.1	Nostoc sp. PCC 7120	Nostoc	Nostocaceae	Nostocales		Cyanobacteria	Bacteria
YP_496193.1	Novosphingobium aromaticivorans	Novosphingobium	Sphingomonadaceae	Sphingomonadales	Alphaproteobacteria	Proteobacteria	Bacteria
YP_497848.1	Novosphingobium aromaticivorans	Novosphingobium	Sphingomonadaceae	Sphingomonadales	Alphaproteobacteria	Proteobacteria	Bacteria
ZP_02154310.1	Oceanibulbus indolifex	Oceanibulbus	Rhodobacteraceae	Rhodobacterales	Alphaproteobacteria	Proteobacteria	Bacteria
ZP_02154201.1	Oceanibulbus indolifex	Oceanibulbus	Rhodobacteraceae	Rhodobacterales	Alphaproteobacteria	Proteobacteria	Bacteria
ZP_02153683.1	Oceanibulbus indolifex	Oceanibulbus	Rhodobacteraceae	Rhodobacterales	Alphaproteobacteria	Proteobacteria	Bacteria
ZP_00953043.1	Oceanicaulis alexandrii	Oceanicaulis	Hyphomonadaceae	Rhodobacterales	Alphaproteobacteria	Proteobacteria	Bacteria
ZP_00953505.1	Oceanicaulis alexandrii	Oceanicaulis	Hyphomonadaceae	Rhodobacterales	Alphaproteobacteria	Proteobacteria	Bacteria
ZP_00997928.1	Oceanicola batsensis	Oceanicola	Rhodobacteraceae	Rhodobacterales	Alphaproteobacteria	Proteobacteria	Bacteria
ZP_01001558.1	Oceanicola batsensis	Oceanicola	Rhodobacteraceae	Rhodobacterales	Alphaproteobacteria	Proteobacteria	Bacteria
ZP_01000584.1	Oceanicola batsensis	Oceanicola	Rhodobacteraceae	Rhodobacterales	Alphaproteobacteria	Proteobacteria	Bacteria
ZP_01157859.1	Oceanicola granulosus	Oceanicola	Rhodobacteraceae	Rhodobacterales	Alphaproteobacteria	Proteobacteria	Bacteria
ZP_01155740.1	Oceanicola granulosus	Oceanicola	Rhodobacteraceae	Rhodobacterales	Alphaproteobacteria	Proteobacteria	Bacteria
ZP_01158274.1	Oceanicola granulosus	Oceanicola	Rhodobacteraceae	Rhodobacterales	Alphaproteobacteria	Proteobacteria	Bacteria
NP_693175.1	Oceanobacillus iheyensis	Oceanobacillus	Bacillaceae	Bacillales	Bacilli	Firmicutes	Bacteria
NP_692359.1	Oceanobacillus iheyensis	Oceanobacillus	Bacillaceae	Bacillales	Bacilli	Firmicutes	Bacteria
NP_692667.1	Oceanobacillus iheyensis	Oceanobacillus	Bacillaceae	Bacillales	Bacilli	Firmicutes	Bacteria
YP_001369142.1	Ochrobactrum anthropi	Ochrobactrum	Brucellaceae	Rhizobiales	Alphaproteobacteria	Proteobacteria	Bacteria
YP_001368609.1	Ochrobactrum anthropi	Ochrobactrum	Brucellaceae	Rhizobiales	Alphaproteobacteria	Proteobacteria	Bacteria
YP_001369029.1	Ochrobactrum anthropi	Ochrobactrum	Brucellaceae	Rhizobiales	Alphaproteobacteria	Proteobacteria	Bacteria
YP_001372916.1	Ochrobactrum anthropi	Ochrobactrum	Brucellaceae	Rhizobiales	Alphaproteobacteria	Proteobacteria	Bacteria
EDY92197.1	Octadecabacter antarcticus	Octadecabacter	Rhodobacteraceae	Rhodobacterales	Alphaproteobacteria	Proteobacteria	Bacteria
YP_002210494.1	Oligotropha carboxidovorans	Oligotropha	Bradyrhizobiaceae	Rhizobiales	Alphaproteobacteria	Proteobacteria	Bacteria
EDT29063.1	Oligotropha carboxidovorans	Oligotropha	Bradyrhizobiaceae	Rhizobiales	Alphaproteobacteria	Proteobacteria	Bacteria
YP_002287693.1	Oligotropha carboxidovorans	Oligotropha	Bradyrhizobiaceae	Rhizobiales	Alphaproteobacteria	Proteobacteria	Bacteria

YP_002290312.1	Oligotropha carboxidovorans	Oligotropha	Bradyrhizobiaceae	Rhizobiales	Alphaproteobacteria	Proteobacteria	Bacteria

Accession	species	genus	order	classe	family	phylum	domain
YP_002213116.1	Oligotropha carboxidovorans	Oligotropha	Bradyrhizobiaceae	Rhizobiales	Alphaproteobacteria	Proteobacteria	Bacteria
YP_002290108.1	Oligotropha carboxidovorans	Oligotropha	Bradyrhizobiaceae	Rhizobiales	Alphaproteobacteria	Proteobacteria	Bacteria
YP_002212912.1	Oligotropha carboxidovorans	Oligotropha	Bradyrhizobiaceae	Rhizobiales	Alphaproteobacteria	Proteobacteria	Bacteria
ZP_02011608.1	Opitutaceae bacterium TAV2		Opitutaceae		Opitutae	Verrucomicrobia	Bacteria
ZP_02013954.1	Opitutaceae bacterium TAV2		Opitutaceae		Opitutae	Verrucomicrobia	Bacteria
YP_001820418.1	Opitutus terrae	Opitutus	Opitutaceae		Opitutae	Verrucomicrobia	Bacteria
YP_001818954.1	Opitutus terrae	Opitutus	Opitutaceae		Opitutae	Verrucomicrobia	Bacteria
YP_001820951.1	Opitutus terrae	Opitutus	Opitutaceae		Opitutae	Verrucomicrobia	Bacteria
YP_001819752.1	Opitutus terrae	Opitutus	Opitutaceae		Opitutae	Verrucomicrobia	Bacteria
YP_001937888.1	Orientia tsutsugamushi	Orientia	Rickettsiaceae	Rickettsiales	Alphaproteobacteria	Proteobacteria	Bacteria
YP_001249069.1	Orientia tsutsugamushi	Orientia	Rickettsiaceae	Rickettsiales	Alphaproteobacteria	Proteobacteria	Bacteria
ZP_02330430.1	Paenibacillus larvae	Paenibacillus	Paenibacillaceae	Bacillales	Bacilli	Firmicutes	Bacteria
ZP_02328499.1	Paenibacillus larvae	Paenibacillus	Paenibacillaceae	Bacillales	Bacilli	Firmicutes	Bacteria
ZP_02845309.1	Paenibacillus sp. JDR-2	Paenibacillus	Paenibacillaceae	Bacillales	Bacilli	Firmicutes	Bacteria
ZP_02850431.1	Paenibacillus sp. JDR-2	Paenibacillus	Paenibacillaceae	Bacillales	Bacilli	Firmicutes	Bacteria
YP_916807.1	Paracoccus denitrificans	Paracoccus	Rhodobacteraceae	Rhodobacterales	Alphaproteobacteria	Proteobacteria	Bacteria
CAA29274.1	Paracoccus denitrificans	Paracoccus	Rhodobacteraceae	Rhodobacterales	Alphaproteobacteria	Proteobacteria	Bacteria
YP_915727.1	Paracoccus denitrificans	Paracoccus	Rhodobacteraceae	Rhodobacterales	Alphaproteobacteria	Proteobacteria	Bacteria
YP_915641.1	Paracoccus denitrificans	Paracoccus	Rhodobacteraceae	Rhodobacterales	Alphaproteobacteria	Proteobacteria	Bacteria
ZP_00629589.1	Paracoccus denitrificans	Paracoccus	Rhodobacteraceae	Rhodobacterales	Alphaproteobacteria	Proteobacteria	Bacteria
YP_916266.1	Paracoccus denitrificans	Paracoccus	Rhodobacteraceae	Rhodobacterales	Alphaproteobacteria	Proteobacteria	Bacteria
ZP_00632126.1	Paracoccus denitrificans	Paracoccus	Rhodobacteraceae	Rhodobacterales	Alphaproteobacteria	Proteobacteria	Bacteria
YP_001411950.1	Parvibaculum lavamentivorans	Parvibaculum	Phyllobacteriaceae	Rhizobiales	Alphaproteobacteria	Proteobacteria	Bacteria
YP_001411844.1	Parvibaculum lavamentivorans	Parvibaculum	Phyllobacteriaceae	Rhizobiales	Alphaproteobacteria	Proteobacteria	Bacteria
YP_001412808.1	Parvibaculum lavamentivorans	Parvibaculum	Phyllobacteriaceae	Rhizobiales	Alphaproteobacteria	Proteobacteria	Bacteria
YP_001412284.1	Parvibaculum lavamentivorans	Parvibaculum	Phyllobacteriaceae	Rhizobiales	Alphaproteobacteria	Proteobacteria	Bacteria
ZP_01016500.1	Parvularcula bermudensis	Parvularcula	Parvularculaceae	Parvularculales	Alphaproteobacteria	Proteobacteria	Bacteria
ACB43160.1	Paulinella chromatophora	Paulinella	Paulinellidae	Euglyphida			Eukaryota
YP_049248.1	Pectobacterium atrosepticum	Pectobacterium	Enterobacteriaceae	Enterobacteriales	Gammaproteobacteria	Proteobacteria	Bacteria
ZP_01883290.1	Pedobacter sp. BAL39	Pedobacter	Sphingobacteriaceae	Sphingobacteriales	Sphingobacteria	Bacteroidetes	Bacteria
ZP_01883863.1	Pedobacter sp. BAL39	Pedobacter	Sphingobacteriaceae	Sphingobacteriales	Sphingobacteria	Bacteroidetes	Bacteria
YP_357934.2	Pelobacter carbinolicus	Pelobacter	Pelobacteraceae	Desulfuromonadales	Deltaproteobacteria	Proteobacteria	Bacteria
YP_002018898.1	Pelodictyon phaeoclathratiforme	Pelodictyon	Chlorobiaceae	Chlorobiales	Chlorobia	Chlorobi	Bacteria
ZP_02145844.1	Phaeobacter gallaeciensis	Phaeobacter	Rhodobacteraceae	Rhodobacterales	Alphaproteobacteria	Proteobacteria	Bacteria
ZP_02147755.1	Phaeobacter gallaeciensis	Phaeobacter	Rhodobacteraceae	Rhodobacterales	Alphaproteobacteria	Proteobacteria	Bacteria
ZP_02150241.1	Phaeobacter gallaeciensis	Phaeobacter	Rhodobacteraceae	Rhodobacterales	Alphaproteobacteria	Proteobacteria	Bacteria
ZP_02151373.1	Phaeobacter gallaeciensis	Phaeobacter	Rhodobacteraceae	Rhodobacterales	Alphaproteobacteria	Proteobacteria	Bacteria

YP_002129386.1	Phenylobacterium zucineum	Phenylobacterium	Caulobacteraceae	Caulobacterales	Alphaproteobacteria	Proteobacteria	Bacteria
YP_002132014.1	Phenylobacterium zucineum	Phenylobacterium	Caulobacteraceae	Caulobacterales	Alphaproteobacteria	Proteobacteria	Bacteria
YP_002130425.1	Phenylobacterium zucineum	Phenylobacterium	Caulobacteraceae	Caulobacterales	Alphaproteobacteria	Proteobacteria	Bacteria
YP_002131652.1	Phenylobacterium zucineum	Phenylobacterium	Caulobacteraceae	Caulobacterales	Alphaproteobacteria	Proteobacteria	Bacteria
YP_002129462.1	Phenylobacterium zucineum	Phenylobacterium	Caulobacteraceae	Caulobacterales	Alphaproteobacteria	Proteobacteria	Bacteria
YP_128409.1	Photobacterium profundum	Photobacterium	Vibrionaceae	Vibrionales	Gammaproteobacteria	Proteobacteria	Bacteria
ZP_01220712.1	Photobacterium profundum	Photobacterium	Vibrionaceae	Vibrionales	Gammaproteobacteria	Proteobacteria	Bacteria
YP_132060.1	Photobacterium profundum	Photobacterium	Vibrionaceae	Vibrionales	Gammaproteobacteria	Proteobacteria	Bacteria

**Accession**	**species**	**genus**	**order**	**classe**	**family**	**phylum**	**domain**
ZP_01219385.1	Photobacterium profundum	Photobacterium	Vibrionaceae	Vibrionales	Gammaproteobacteria	Proteobacteria	Bacteria
ZP_01217907.1	Photobacterium profundum	Photobacterium	Vibrionaceae	Vibrionales	Gammaproteobacteria	Proteobacteria	Bacteria
YP_130037.1	Photobacterium profundum	Photobacterium	Vibrionaceae	Vibrionales	Gammaproteobacteria	Proteobacteria	Bacteria
ZP_01223000.1	Photobacterium profundum	Photobacterium	Vibrionaceae	Vibrionales	Gammaproteobacteria	Proteobacteria	Bacteria
ZP_01219400.1	Photobacterium profundum	Photobacterium	Vibrionaceae	Vibrionales	Gammaproteobacteria	Proteobacteria	Bacteria
ZP_01220308.1	Photobacterium profundum	Photobacterium	Vibrionaceae	Vibrionales	Gammaproteobacteria	Proteobacteria	Bacteria
YP_132726.1	Photobacterium profundum	Photobacterium	Vibrionaceae	Vibrionales	Gammaproteobacteria	Proteobacteria	Bacteria
ZP_01162438.1	Photobacterium sp. SKA34	Photobacterium	Vibrionaceae	Vibrionales	Gammaproteobacteria	Proteobacteria	Bacteria
ZP_01158716.1	Photobacterium sp. SKA34	Photobacterium	Vibrionaceae	Vibrionales	Gammaproteobacteria	Proteobacteria	Bacteria
ZP_01160483.1	Photobacterium sp. SKA34	Photobacterium	Vibrionaceae	Vibrionales	Gammaproteobacteria	Proteobacteria	Bacteria
NP_931081.1	Photorhabdus luminescens	Photorhabdus	Enterobacteriaceae	Enterobacteriales	Gammaproteobacteria	Proteobacteria	Bacteria
YP_024082.1	Picrophilus torridus	Picrophilus	Picrophilaceae	Thermoplasmatales	Thermoplasmata	Euryarchaeota	Archaea
ZP_01855645.1	Planctomyces maris	Planctomyces	Planctomycetaceae	Planctomycetales	Planctomycetacia	Planctomycetes	Bacteria
ZP_01852349.1	Planctomyces maris	Planctomyces	Planctomycetaceae	Planctomycetales	Planctomycetacia	Planctomycetes	Bacteria
ZP_01854217.1	Planctomyces maris	Planctomyces	Planctomycetaceae	Planctomycetales	Planctomycetacia	Planctomycetes	Bacteria
ZP_01857356.1	Planctomyces maris	Planctomyces	Planctomycetaceae	Planctomycetales	Planctomycetacia	Planctomycetes	Bacteria
ZP_01911748.1	Plesiocystis pacifica	Plesiocystis	Nannocystaceae	Myxococcales	Deltaproteobacteria	Proteobacteria	Bacteria
ZP_01117638.1	Polaribacter irgensii	Polaribacter	Flavobacteriaceae	Flavobacteriales	Flavobacteria	Bacteroidetes	Bacteria
ZP_01053676.1	Polaribacter sp. MED152	Polaribacter	Flavobacteriaceae	Flavobacteriales	Flavobacteria	Bacteroidetes	Bacteria
ZP_01052258.1	Polaribacter sp. MED152	Polaribacter	Flavobacteriaceae	Flavobacteriales	Flavobacteria	Bacteroidetes	Bacteria
YP_981117.1	Polaromonas naphthalenivorans	Polaromonas	Comamonadaceae	Burkholderiales	Betaproteobacteria	Proteobacteria	Bacteria
YP_983999.1	Polaromonas naphthalenivorans	Polaromonas	Comamonadaceae	Burkholderiales	Betaproteobacteria	Proteobacteria	Bacteria
YP_981564.1	Polaromonas naphthalenivorans	Polaromonas	Comamonadaceae	Burkholderiales	Betaproteobacteria	Proteobacteria	Bacteria
YP_548095.1	Polaromonas sp. JS666	Polaromonas	Comamonadaceae	Burkholderiales	Betaproteobacteria	Proteobacteria	Bacteria
YP_547581.1	Polaromonas sp. JS666	Polaromonas	Comamonadaceae	Burkholderiales	Betaproteobacteria	Proteobacteria	Bacteria
YP_551095.1	Polaromonas sp. JS666	Polaromonas	Comamonadaceae	Burkholderiales	Betaproteobacteria	Proteobacteria	Bacteria
YP_548116.1	Polaromonas sp. JS666	Polaromonas	Comamonadaceae	Burkholderiales	Betaproteobacteria	Proteobacteria	Bacteria
YP_001798332.1	Polynucleobacter necessarius	Polynucleobacter	Burkholderiaceae	Burkholderiales	Betaproteobacteria	Proteobacteria	Bacteria
YP_001156715.1	Polynucleobacter necessarius	Polynucleobacter	Burkholderiaceae	Burkholderiales	Betaproteobacteria	Proteobacteria	Bacteria

YP_001798088.1	Polynucleobacter necessarius	Polynucleobacter	Burkholderiaceae	Burkholderiales	Betaproteobacteria	Proteobacteria	Bacteria
YP_001155237.1	Polynucleobacter necessarius	Polynucleobacter	Burkholderiaceae	Burkholderiales	Betaproteobacteria	Proteobacteria	Bacteria
YP_001011578.1	Prochlorococcus marinus	Prochlorococcus	Prochlorococcaceae	Prochlorales		Cyanobacteria	Bacteria
NP_892563.1	Prochlorococcus marinus	Prochlorococcus	Prochlorococcaceae	Prochlorales		Cyanobacteria	Bacteria
YP_001008895.1	Prochlorococcus marinus	Prochlorococcus	Prochlorococcaceae	Prochlorales		Cyanobacteria	Bacteria
YP_001090693.1	Prochlorococcus marinus	Prochlorococcus	Prochlorococcaceae	Prochlorales		Cyanobacteria	Bacteria
YP_396941.1	Prochlorococcus marinus	Prochlorococcus	Prochlorococcaceae	Prochlorales		Cyanobacteria	Bacteria
YP_001484863.1	Prochlorococcus marinus	Prochlorococcus	Prochlorococcaceae	Prochlorales		Cyanobacteria	Bacteria
YP_001014326.1	Prochlorococcus marinus	Prochlorococcus	Prochlorococcaceae	Prochlorales		Cyanobacteria	Bacteria
YP_292967.1	Prochlorococcus marinus	Prochlorococcus	Prochlorococcaceae	Prochlorales		Cyanobacteria	Bacteria
YP_001550327.1	Prochlorococcus marinus	Prochlorococcus	Prochlorococcaceae	Prochlorales		Cyanobacteria	Bacteria
NP_874835.1	Prochlorococcus marinus	Prochlorococcus	Prochlorococcaceae	Prochlorales		Cyanobacteria	Bacteria
YP_001016658.1	Prochlorococcus marinus	Prochlorococcus	Prochlorococcaceae	Prochlorales		Cyanobacteria	Bacteria
NP_895169.1	Prochlorococcus marinus	Prochlorococcus	Prochlorococcaceae	Prochlorales		Cyanobacteria	Bacteria
YP_001010823.1	Prochlorococcus marinus	Prochlorococcus	Prochlorococcaceae	Prochlorales		Cyanobacteria	Bacteria
YP_001483726.1	Prochlorococcus marinus	Prochlorococcus	Prochlorococcaceae	Prochlorales		Cyanobacteria	Bacteria

Accession	species	genus	order	classe	family	phylum	domain
YP_055417.1	Propionibacterium acnes	Propionibacterium	Propionibacteriaceae	Actinomycetales	Actinobacteria (class)	Actinobacteria	Bacteria
YP_056649.1	Propionibacterium acnes	Propionibacterium	Propionibacteriaceae	Actinomycetales	Actinobacteria (class)	Actinobacteria	Bacteria
YP_002149891.1	Proteus mirabilis	Proteus	Enterobacteriaceae	Enterobacteriales	Gammaproteobacteria	Proteobacteria	Bacteria
ZP_02962546.1	Providencia stuartii	Providencia	Enterobacteriaceae	Enterobacteriales	Gammaproteobacteria	Proteobacteria	Bacteria
YP_663798.1	Pseudoalteromonas atlantica	Pseudoalteromonas	Pseudoalteromonadacea	Alteromonadales	Gammaproteobacteria	Proteobacteria	Bacteria
YP_660871.1	Pseudoalteromonas atlantica	Pseudoalteromonas	Pseudoalteromonadacea	Alteromonadales	Gammaproteobacteria	Proteobacteria	Bacteria
YP_661645.1	Pseudoalteromonas atlantica	Pseudoalteromonas	Pseudoalteromonadacea	Alteromonadales	Gammaproteobacteria	Proteobacteria	Bacteria
YP_341349.1	Pseudoalteromonas haloplanktis	Pseudoalteromonas	Pseudoalteromonadacea	Alteromonadales	Gammaproteobacteria	Proteobacteria	Bacteria
YP_340728.1	Pseudoalteromonas haloplanktis	Pseudoalteromonas	Pseudoalteromonadacea	Alteromonadales	Gammaproteobacteria	Proteobacteria	Bacteria
YP_340357.1	Pseudoalteromonas haloplanktis	Pseudoalteromonas	Pseudoalteromonadacea	Alteromonadales	Gammaproteobacteria	Proteobacteria	Bacteria
YP_340908.1	Pseudoalteromonas haloplanktis	Pseudoalteromonas	Pseudoalteromonadacea	Alteromonadales	Gammaproteobacteria	Proteobacteria	Bacteria
ZP_01135737.1	Pseudoalteromonas tunicata	Pseudoalteromonas	Pseudoalteromonadacea	Alteromonadales	Gammaproteobacteria	Proteobacteria	Bacteria
ZP_01134282.1	Pseudoalteromonas tunicata	Pseudoalteromonas	Pseudoalteromonadacea	Alteromonadales	Gammaproteobacteria	Proteobacteria	Bacteria
YP_788264.1	Pseudomonas aeruginosa	Pseudomonas	Pseudomonadaceae	Pseudomonadales	Gammaproteobacteria	Proteobacteria	Bacteria
NP_248796.1	Pseudomonas aeruginosa	Pseudomonas	Pseudomonadaceae	Pseudomonadales	Gammaproteobacteria	Proteobacteria	Bacteria
YP_002437715.1	Pseudomonas aeruginosa	Pseudomonas	Pseudomonadaceae	Pseudomonadales	Gammaproteobacteria	Proteobacteria	Bacteria
YP_001345577.1	Pseudomonas aeruginosa	Pseudomonas	Pseudomonadaceae	Pseudomonadales	Gammaproteobacteria	Proteobacteria	Bacteria
YP_002441446.1	Pseudomonas aeruginosa	Pseudomonas	Pseudomonadaceae	Pseudomonadales	Gammaproteobacteria	Proteobacteria	Bacteria
YP_791927.1	Pseudomonas aeruginosa	Pseudomonas	Pseudomonadaceae	Pseudomonadales	Gammaproteobacteria	Proteobacteria	Bacteria
NP_250009.1	Pseudomonas aeruginosa	Pseudomonas	Pseudomonadaceae	Pseudomonadales	Gammaproteobacteria	Proteobacteria	Bacteria
YP_001349426.1	Pseudomonas aeruginosa	Pseudomonas	Pseudomonadaceae	Pseudomonadales	Gammaproteobacteria	Proteobacteria	Bacteria

YP_002438446.1	Pseudomonas aeruginosa	Pseudomonas	Pseudomonadaceae	Pseudomonadales	Gammaproteobacteria	Proteobacteria	Bacteria
YP_788989.1	Pseudomonas aeruginosa	Pseudomonas	Pseudomonadaceae	Pseudomonadales	Gammaproteobacteria	Proteobacteria	Bacteria
NP_252822.1	Pseudomonas aeruginosa	Pseudomonas	Pseudomonadaceae	Pseudomonadales	Gammaproteobacteria	Proteobacteria	Bacteria
YP_001346344.1	Pseudomonas aeruginosa	Pseudomonas	Pseudomonadaceae	Pseudomonadales	Gammaproteobacteria	Proteobacteria	Bacteria
YP_791698.1	Pseudomonas aeruginosa	Pseudomonas	Pseudomonadaceae	Pseudomonadales	Gammaproteobacteria	Proteobacteria	Bacteria
NP_250245.1	Pseudomonas aeruginosa	Pseudomonas	Pseudomonadaceae	Pseudomonadales	Gammaproteobacteria	Proteobacteria	Bacteria
YP_002441357.1	Pseudomonas aeruginosa	Pseudomonas	Pseudomonadaceae	Pseudomonadales	Gammaproteobacteria	Proteobacteria	Bacteria
YP_001349131.1	Pseudomonas aeruginosa	Pseudomonas	Pseudomonadaceae	Pseudomonadales	Gammaproteobacteria	Proteobacteria	Bacteria
YP_002441054.1	Pseudomonas aeruginosa	Pseudomonas	Pseudomonadaceae	Pseudomonadales	Gammaproteobacteria	Proteobacteria	Bacteria
NP_250547.1	Pseudomonas aeruginosa	Pseudomonas	Pseudomonadaceae	Pseudomonadales	Gammaproteobacteria	Proteobacteria	Bacteria
YP_791385.1	Pseudomonas aeruginosa	Pseudomonas	Pseudomonadaceae	Pseudomonadales	Gammaproteobacteria	Proteobacteria	Bacteria
YP_001348796.1	Pseudomonas aeruginosa	Pseudomonas	Pseudomonadaceae	Pseudomonadales	Gammaproteobacteria	Proteobacteria	Bacteria
YP_002441354.1	Pseudomonas aeruginosa	Pseudomonas	Pseudomonadaceae	Pseudomonadales	Gammaproteobacteria	Proteobacteria	Bacteria
YP_791695.1	Pseudomonas aeruginosa	Pseudomonas	Pseudomonadaceae	Pseudomonadales	Gammaproteobacteria	Proteobacteria	Bacteria
YP_001349127.1	Pseudomonas aeruginosa	Pseudomonas	Pseudomonadaceae	Pseudomonadales	Gammaproteobacteria	Proteobacteria	Bacteria
NP_250248.1	Pseudomonas aeruginosa	Pseudomonas	Pseudomonadaceae	Pseudomonadales	Gammaproteobacteria	Proteobacteria	Bacteria
NP_249215.1	Pseudomonas aeruginosa	Pseudomonas	Pseudomonadaceae	Pseudomonadales	Gammaproteobacteria	Proteobacteria	Bacteria
ZP_01363428.1	Pseudomonas aeruginosa	Pseudomonas	Pseudomonadaceae	Pseudomonadales	Gammaproteobacteria	Proteobacteria	Bacteria
YP_002438129.1	Pseudomonas aeruginosa	Pseudomonas	Pseudomonadaceae	Pseudomonadales	Gammaproteobacteria	Proteobacteria	Bacteria
YP_788694.1	Pseudomonas aeruginosa	Pseudomonas	Pseudomonadaceae	Pseudomonadales	Gammaproteobacteria	Proteobacteria	Bacteria
YP_001346020.1	Pseudomonas aeruginosa	Pseudomonas	Pseudomonadaceae	Pseudomonadales	Gammaproteobacteria	Proteobacteria	Bacteria
YP_605868.1	Pseudomonas entomophila	Pseudomonas	Pseudomonadaceae	Pseudomonadales	Gammaproteobacteria	Proteobacteria	Bacteria
YP_606676.1	Pseudomonas entomophila	Pseudomonas	Pseudomonadaceae	Pseudomonadales	Gammaproteobacteria	Proteobacteria	Bacteria
YP_607451.1	Pseudomonas entomophila	Pseudomonas	Pseudomonadaceae	Pseudomonadales	Gammaproteobacteria	Proteobacteria	Bacteria

Accession	species	genus	order	classe	family	phylum	domain
YP_607455.1	Pseudomonas entomophila	Pseudomonas	Pseudomonadaceae	Pseudomonadales	Gammaproteobacteria	Proteobacteria	Bacteria
YP_345811.1	Pseudomonas fluorescens	Pseudomonas	Pseudomonadaceae	Pseudomonadales	Gammaproteobacteria	Proteobacteria	Bacteria
YP_257214.1	Pseudomonas fluorescens	Pseudomonas	Pseudomonadaceae	Pseudomonadales	Gammaproteobacteria	Proteobacteria	Bacteria
YP_350375.1	Pseudomonas fluorescens	Pseudomonas	Pseudomonadaceae	Pseudomonadales	Gammaproteobacteria	Proteobacteria	Bacteria
YP_262115.1	Pseudomonas fluorescens	Pseudomonas	Pseudomonadaceae	Pseudomonadales	Gammaproteobacteria	Proteobacteria	Bacteria
YP_261816.1	Pseudomonas fluorescens	Pseudomonas	Pseudomonadaceae	Pseudomonadales	Gammaproteobacteria	Proteobacteria	Bacteria
YP_348243.1	Pseudomonas fluorescens	Pseudomonas	Pseudomonadaceae	Pseudomonadales	Gammaproteobacteria	Proteobacteria	Bacteria
YP_259940.1	Pseudomonas fluorescens	Pseudomonas	Pseudomonadaceae	Pseudomonadales	Gammaproteobacteria	Proteobacteria	Bacteria
YP_347555.1	Pseudomonas fluorescens	Pseudomonas	Pseudomonadaceae	Pseudomonadales	Gammaproteobacteria	Proteobacteria	Bacteria
YP_259040.1	Pseudomonas fluorescens	Pseudomonas	Pseudomonadaceae	Pseudomonadales	Gammaproteobacteria	Proteobacteria	Bacteria
YP_347559.1	Pseudomonas fluorescens	Pseudomonas	Pseudomonadaceae	Pseudomonadales	Gammaproteobacteria	Proteobacteria	Bacteria
YP_259044.1	Pseudomonas fluorescens	Pseudomonas	Pseudomonadaceae	Pseudomonadales	Gammaproteobacteria	Proteobacteria	Bacteria
YP_001185649.1	Pseudomonas mendocina	Pseudomonas	Pseudomonadaceae	Pseudomonadales	Gammaproteobacteria	Proteobacteria	Bacteria

YP_001187757.1	Pseudomonas mendocina	Pseudomonas	Pseudomonadaceae	Pseudomonadales	Gammaproteobacteria	Proteobacteria	Bacteria
YP_001188092.1	Pseudomonas mendocina	Pseudomonas	Pseudomonadaceae	Pseudomonadales	Gammaproteobacteria	Proteobacteria	Bacteria
YP_001188087.1	Pseudomonas mendocina	Pseudomonas	Pseudomonadaceae	Pseudomonadales	Gammaproteobacteria	Proteobacteria	Bacteria
YP_001666370.1	Pseudomonas putida	Pseudomonas	Pseudomonadaceae	Pseudomonadales	Gammaproteobacteria	Proteobacteria	Bacteria
NP_742274.1	Pseudomonas putida	Pseudomonas	Pseudomonadaceae	Pseudomonadales	Gammaproteobacteria	Proteobacteria	Bacteria
YP_001265479.1	Pseudomonas putida	Pseudomonas	Pseudomonadaceae	Pseudomonadales	Gammaproteobacteria	Proteobacteria	Bacteria
YP_001746998.1	Pseudomonas putida	Pseudomonas	Pseudomonadaceae	Pseudomonadales	Gammaproteobacteria	Proteobacteria	Bacteria
NP_742974.1	Pseudomonas putida	Pseudomonas	Pseudomonadaceae	Pseudomonadales	Gammaproteobacteria	Proteobacteria	Bacteria
YP_001266182.1	Pseudomonas putida	Pseudomonas	Pseudomonadaceae	Pseudomonadales	Gammaproteobacteria	Proteobacteria	Bacteria
YP_001751224.1	Pseudomonas putida	Pseudomonas	Pseudomonadaceae	Pseudomonadales	Gammaproteobacteria	Proteobacteria	Bacteria
YP_001667096.1	Pseudomonas putida	Pseudomonas	Pseudomonadaceae	Pseudomonadales	Gammaproteobacteria	Proteobacteria	Bacteria
NP_746371.1	Pseudomonas putida	Pseudomonas	Pseudomonadaceae	Pseudomonadales	Gammaproteobacteria	Proteobacteria	Bacteria
YP_001266953.1	Pseudomonas putida	Pseudomonas	Pseudomonadaceae	Pseudomonadales	Gammaproteobacteria	Proteobacteria	Bacteria
YP_001670046.1	Pseudomonas putida	Pseudomonas	Pseudomonadaceae	Pseudomonadales	Gammaproteobacteria	Proteobacteria	Bacteria
YP_001750424.1	Pseudomonas putida	Pseudomonas	Pseudomonadaceae	Pseudomonadales	Gammaproteobacteria	Proteobacteria	Bacteria
YP_001266958.1	Pseudomonas putida	Pseudomonas	Pseudomonadaceae	Pseudomonadales	Gammaproteobacteria	Proteobacteria	Bacteria
NP_746366.1	Pseudomonas putida	Pseudomonas	Pseudomonadaceae	Pseudomonadales	Gammaproteobacteria	Proteobacteria	Bacteria
YP_001670042.1	Pseudomonas putida	Pseudomonas	Pseudomonadaceae	Pseudomonadales	Gammaproteobacteria	Proteobacteria	Bacteria
YP_001750420.1	Pseudomonas putida	Pseudomonas	Pseudomonadaceae	Pseudomonadales	Gammaproteobacteria	Proteobacteria	Bacteria
AAL37189.1	Pseudomonas putida	Pseudomonas	Pseudomonadaceae	Pseudomonadales	Gammaproteobacteria	Proteobacteria	Bacteria
BAA76357.1	Pseudomonas putida	Pseudomonas	Pseudomonadaceae	Pseudomonadales	Gammaproteobacteria	Proteobacteria	Bacteria
YP_273477.1	Pseudomonas savastanoi	Pseudomonas	Pseudomonadaceae	Pseudomonadales	Gammaproteobacteria	Proteobacteria	Bacteria
YP_275495.1	Pseudomonas savastanoi	Pseudomonas	Pseudomonadaceae	Pseudomonadales	Gammaproteobacteria	Proteobacteria	Bacteria
AAC79449.1	Pseudomonas sp. G-179		Rhizobiaceae	Rhizobiales	Alphaproteobacteria	Proteobacteria	Bacteria
YP_001174628.1	Pseudomonas stutzeri	Pseudomonas	Pseudomonadaceae	Pseudomonadales	Gammaproteobacteria	Proteobacteria	Bacteria
YP_001172366.1	Pseudomonas stutzeri	Pseudomonas	Pseudomonadaceae	Pseudomonadales	Gammaproteobacteria	Proteobacteria	Bacteria
YP_001172356.1	Pseudomonas stutzeri	Pseudomonas	Pseudomonadaceae	Pseudomonadales	Gammaproteobacteria	Proteobacteria	Bacteria
YP_001172359.1	Pseudomonas stutzeri	Pseudomonas	Pseudomonadaceae	Pseudomonadales	Gammaproteobacteria	Proteobacteria	Bacteria
YP_001173991.1	Pseudomonas stutzeri	Pseudomonas	Pseudomonadaceae	Pseudomonadales	Gammaproteobacteria	Proteobacteria	Bacteria
YP_234234.1	Pseudomonas syringae	Pseudomonas	Pseudomonadaceae	Pseudomonadales	Gammaproteobacteria	Proteobacteria	Bacteria
YP_236483.1	Pseudomonas syringae	Pseudomonas	Pseudomonadaceae	Pseudomonadales	Gammaproteobacteria	Proteobacteria	Bacteria
NP_791153.1	Pseudomonas syringae group genomosp. 3	Pseudomonas	Pseudomonadaceae	Pseudomonadales	Gammaproteobacteria	Proteobacteria	Bacteria

Accession	species	genus	order	classe	family	phylum	domain
NP_791827.1	Pseudomonas syringae group genomosp. 3	Pseudomonas	Pseudomonadaceae	Pseudomonadales	Gammaproteobacteria	Proteobacteria	Bacteria
EEA94034.1	Pseudovibrio sp. JE062	Pseudovibrio	Rhodobacteraceae	Rhodobacterales	Alphaproteobacteria	Proteobacteria	Bacteria
EEA93213.1	Pseudovibrio sp. JE062	Pseudovibrio	Rhodobacteraceae	Rhodobacterales	Alphaproteobacteria	Proteobacteria	Bacteria
EEA94158.1	Pseudovibrio sp. JE062	Pseudovibrio	Rhodobacteraceae	Rhodobacterales	Alphaproteobacteria	Proteobacteria	Bacteria
EEA93383.1	Pseudovibrio sp. JE062	Pseudovibrio	Rhodobacteraceae	Rhodobacterales	Alphaproteobacteria	Proteobacteria	Bacteria

EEA92005.1	Pseudovibrio sp. JE062	Pseudovibrio	Rhodobacteraceae	Rhodobacterales	Alphaproteobacteria	Proteobacteria	Bacteria
YP_264065.1	Psychrobacter arcticus	Psychrobacter	Moraxellaceae	Pseudomonadales	Gammaproteobacteria	Proteobacteria	Bacteria
YP_580051.1	Psychrobacter cryohalolentis	Psychrobacter	Moraxellaceae	Pseudomonadales	Gammaproteobacteria	Proteobacteria	Bacteria
YP_001280634.1	Psychrobacter sp. PRwf-1	Psychrobacter	Moraxellaceae	Pseudomonadales	Gammaproteobacteria	Proteobacteria	Bacteria
YP_001280413.1	Psychrobacter sp. PRwf-1	Psychrobacter	Moraxellaceae	Pseudomonadales	Gammaproteobacteria	Proteobacteria	Bacteria
ZP_01253939.1	Psychroflexus torquis	Psychroflexus	Flavobacteriaceae	Flavobacteriales	Flavobacteria	Bacteroidetes	Bacteria
ZP_01254673.1	Psychroflexus torquis	Psychroflexus	Flavobacteriaceae	Flavobacteriales	Flavobacteria	Bacteroidetes	Bacteria
YP_944025.1	Psychromonas ingrahamii	Psychromonas	Psychromonadaceae	Alteromonadales	Gammaproteobacteria	Proteobacteria	Bacteria
YP_942380.1	Psychromonas ingrahamii	Psychromonas	Psychromonadaceae	Alteromonadales	Gammaproteobacteria	Proteobacteria	Bacteria
ZP_01215617.1	Psychromonas sp. CNPT3	Psychromonas	Psychromonadaceae	Alteromonadales	Gammaproteobacteria	Proteobacteria	Bacteria
NP_559235.1	Pyrobaculum aerophilum	Pyrobaculum	Thermoproteaceae	Thermoproteales	Thermoprotei	Crenarchaeota	Archaea
NP_559246.1	Pyrobaculum aerophilum	Pyrobaculum	Thermoproteaceae	Thermoproteales	Thermoprotei	Crenarchaeota	Archaea
NP_560853.1	Pyrobaculum aerophilum	Pyrobaculum	Thermoproteaceae	Thermoproteales	Thermoprotei	Crenarchaeota	Archaea
YP_001152749.1	Pyrobaculum arsenaticum	Pyrobaculum	Thermoproteaceae	Thermoproteales	Thermoprotei	Crenarchaeota	Archaea
YP_001056825.1	Pyrobaculum calidifontis	Pyrobaculum	Thermoproteaceae	Thermoproteales	Thermoprotei	Crenarchaeota	Archaea
YP_001056831.1	Pyrobaculum calidifontis	Pyrobaculum	Thermoproteaceae	Thermoproteales	Thermoprotei	Crenarchaeota	Archaea
YP_001056790.1	Pyrobaculum calidifontis	Pyrobaculum	Thermoproteaceae	Thermoproteales	Thermoprotei	Crenarchaeota	Archaea
BAC56132.1	Pyrobaculum oguniense	Pyrobaculum	Thermoproteaceae	Thermoproteales	Thermoprotei	Crenarchaeota	Archaea
BAC56140.1	Pyrobaculum oguniense	Pyrobaculum	Thermoproteaceae	Thermoproteales	Thermoprotei	Crenarchaeota	Archaea
ZP_02005784.1	Ralstonia pickettii	Ralstonia	Burkholderiaceae	Burkholderiales	Betaproteobacteria	Proteobacteria	Bacteria
ZP_02005338.1	Ralstonia pickettii	Ralstonia	Burkholderiaceae	Burkholderiales	Betaproteobacteria	Proteobacteria	Bacteria
ZP_02008323.1	Ralstonia pickettii	Ralstonia	Burkholderiaceae	Burkholderiales	Betaproteobacteria	Proteobacteria	Bacteria
ZP_02009629.1	Ralstonia pickettii	Ralstonia	Burkholderiaceae	Burkholderiales	Betaproteobacteria	Proteobacteria	Bacteria
ZP_02007918.1	Ralstonia pickettii	Ralstonia	Burkholderiaceae	Burkholderiales	Betaproteobacteria	Proteobacteria	Bacteria
ZP_02008815.1	Ralstonia pickettii	Ralstonia	Burkholderiaceae	Burkholderiales	Betaproteobacteria	Proteobacteria	Bacteria
ZP_02005927.1	Ralstonia pickettii	Ralstonia	Burkholderiaceae	Burkholderiales	Betaproteobacteria	Proteobacteria	Bacteria
NP_518484.1	Ralstonia solanacearum	Ralstonia	Burkholderiaceae	Burkholderiales	Betaproteobacteria	Proteobacteria	Bacteria
NP_523124.1	Ralstonia solanacearum	Ralstonia	Burkholderiaceae	Burkholderiales	Betaproteobacteria	Proteobacteria	Bacteria
NP_519980.1	Ralstonia solanacearum	Ralstonia	Burkholderiaceae	Burkholderiales	Betaproteobacteria	Proteobacteria	Bacteria
NP_521037.1	Ralstonia solanacearum	Ralstonia	Burkholderiaceae	Burkholderiales	Betaproteobacteria	Proteobacteria	Bacteria
NP_519397.1	Ralstonia solanacearum	Ralstonia	Burkholderiaceae	Burkholderiales	Betaproteobacteria	Proteobacteria	Bacteria
ZP_00945695.1	Ralstonia solanacearum	Ralstonia	Burkholderiaceae	Burkholderiales	Betaproteobacteria	Proteobacteria	Bacteria
ZP_00944709.1	Ralstonia solanacearum	Ralstonia	Burkholderiaceae	Burkholderiales	Betaproteobacteria	Proteobacteria	Bacteria
ZP_00946507.1	Ralstonia solanacearum	Ralstonia	Burkholderiaceae	Burkholderiales	Betaproteobacteria	Proteobacteria	Bacteria
ZP_01114101.1	Reinekea sp. MED297	Reinekea			Gammaproteobacteria	Proteobacteria	Bacteria
ZP_01113912.1	Reinekea sp. MED297	Reinekea			Gammaproteobacteria	Proteobacteria	Bacteria
YP_001624733.1	Renibacterium salmoninarum	Renibacterium	Micrococcaceae	Actinomycetales	Actinobacteria (class)	Actinobacteria	Bacteria
AAP22949.1	Rhizobium arachis	Rhizobium	Rhizobiaceae	Rhizobiales	Alphaproteobacteria	Proteobacteria	Bacteria
YP_001977211.1	Rhizobium etli	Rhizobium	Rhizobiaceae	Rhizobiales	Alphaproteobacteria	Proteobacteria	Bacteria
YP_468491.1	Rhizobium etli	Rhizobium	Rhizobiaceae	Rhizobiales	Alphaproteobacteria	Proteobacteria	Bacteria

Accession	species	genus	order	classe	family	phylum	domain
YP_001978875.1	Rhizobium etli	Rhizobium	Rhizobiaceae	Rhizobiales	Alphaproteobacteria	Proteobacteria	Bacteria
YP_470115.1	Rhizobium etli	Rhizobium	Rhizobiaceae	Rhizobiales	Alphaproteobacteria	Proteobacteria	Bacteria
YP_472195.1	Rhizobium etli	Rhizobium	Rhizobiaceae	Rhizobiales	Alphaproteobacteria	Proteobacteria	Bacteria
YP_471835.1	Rhizobium etli	Rhizobium	Rhizobiaceae	Rhizobiales	Alphaproteobacteria	Proteobacteria	Bacteria
YP_468520.1	Rhizobium etli	Rhizobium	Rhizobiaceae	Rhizobiales	Alphaproteobacteria	Proteobacteria	Bacteria
AAC15888.1	Rhizobium etli	Rhizobium	Rhizobiaceae	Rhizobiales	Alphaproteobacteria	Proteobacteria	Bacteria
YP_473132.1	Rhizobium etli	Rhizobium	Rhizobiaceae	Rhizobiales	Alphaproteobacteria	Proteobacteria	Bacteria
YP_766633.1	Rhizobium leguminosarum	Rhizobium	Rhizobiaceae	Rhizobiales	Alphaproteobacteria	Proteobacteria	Bacteria
ZP_02293248.1	Rhizobium leguminosarum	Rhizobium	Rhizobiaceae	Rhizobiales	Alphaproteobacteria	Proteobacteria	Bacteria
YP_002280126.1	Rhizobium leguminosarum	Rhizobium	Rhizobiaceae	Rhizobiales	Alphaproteobacteria	Proteobacteria	Bacteria
YP_002281771.1	Rhizobium leguminosarum	Rhizobium	Rhizobiaceae	Rhizobiales	Alphaproteobacteria	Proteobacteria	Bacteria
YP_768626.1	Rhizobium leguminosarum	Rhizobium	Rhizobiaceae	Rhizobiales	Alphaproteobacteria	Proteobacteria	Bacteria
ZP_02295062.1	Rhizobium leguminosarum	Rhizobium	Rhizobiaceae	Rhizobiales	Alphaproteobacteria	Proteobacteria	Bacteria
ZP_02295284.1	Rhizobium leguminosarum	Rhizobium	Rhizobiaceae	Rhizobiales	Alphaproteobacteria	Proteobacteria	Bacteria
ZP_02292511.1	Rhizobium leguminosarum	Rhizobium	Rhizobiaceae	Rhizobiales	Alphaproteobacteria	Proteobacteria	Bacteria
ZP_02293874.1	Rhizobium leguminosarum	Rhizobium	Rhizobiaceae	Rhizobiales	Alphaproteobacteria	Proteobacteria	Bacteria
YP_765315.1	Rhizobium leguminosarum	Rhizobium	Rhizobiaceae	Rhizobiales	Alphaproteobacteria	Proteobacteria	Bacteria
YP_766146.1	Rhizobium leguminosarum	Rhizobium	Rhizobiaceae	Rhizobiales	Alphaproteobacteria	Proteobacteria	Bacteria
AAC46108.1	Rhodobacter capsulatus	Rhodobacter	Rhodobacteraceae	Rhodobacterales	Alphaproteobacteria	Proteobacteria	Bacteria
YP_001042415.1	Rhodobacter sphaeroides	Rhodobacter	Rhodobacteraceae	Rhodobacterales	Alphaproteobacteria	Proteobacteria	Bacteria
YP_351928.1	Rhodobacter sphaeroides	Rhodobacter	Rhodobacteraceae	Rhodobacterales	Alphaproteobacteria	Proteobacteria	Bacteria
YP_001166875.1	Rhodobacter sphaeroides	Rhodobacter	Rhodobacteraceae	Rhodobacterales	Alphaproteobacteria	Proteobacteria	Bacteria
YP_001045676.1	Rhodobacter sphaeroides	Rhodobacter	Rhodobacteraceae	Rhodobacterales	Alphaproteobacteria	Proteobacteria	Bacteria
YP_354614.1	Rhodobacter sphaeroides	Rhodobacter	Rhodobacteraceae	Rhodobacterales	Alphaproteobacteria	Proteobacteria	Bacteria
YP_001043635.1	Rhodobacter sphaeroides	Rhodobacter	Rhodobacteraceae	Rhodobacterales	Alphaproteobacteria	Proteobacteria	Bacteria
YP_353192.1	Rhodobacter sphaeroides	Rhodobacter	Rhodobacteraceae	Rhodobacterales	Alphaproteobacteria	Proteobacteria	Bacteria
YP_001167902.1	Rhodobacter sphaeroides	Rhodobacter	Rhodobacteraceae	Rhodobacterales	Alphaproteobacteria	Proteobacteria	Bacteria
YP_001044225.1	Rhodobacter sphaeroides	Rhodobacter	Rhodobacteraceae	Rhodobacterales	Alphaproteobacteria	Proteobacteria	Bacteria
YP_353773.1	Rhodobacter sphaeroides	Rhodobacter	Rhodobacteraceae	Rhodobacterales	Alphaproteobacteria	Proteobacteria	Bacteria
AAB02556.1	Rhodobacter sphaeroides	Rhodobacter	Rhodobacteraceae	Rhodobacterales	Alphaproteobacteria	Proteobacteria	Bacteria
YP_001166745.1	Rhodobacter sphaeroides	Rhodobacter	Rhodobacteraceae	Rhodobacterales	Alphaproteobacteria	Proteobacteria	Bacteria
YP_001043844.1	Rhodobacter sphaeroides	Rhodobacter	Rhodobacteraceae	Rhodobacterales	Alphaproteobacteria	Proteobacteria	Bacteria
YP_353400.1	Rhodobacter sphaeroides	Rhodobacter	Rhodobacteraceae	Rhodobacterales	Alphaproteobacteria	Proteobacteria	Bacteria
YP_001167177.1	Rhodobacter sphaeroides	Rhodobacter	Rhodobacteraceae	Rhodobacterales	Alphaproteobacteria	Proteobacteria	Bacteria
ZP_01450302.1	Rhodobacterales bacterium HTCC2255			Rhodobacterales	Alphaproteobacteria	Proteobacteria	Bacteria
ZP_01449141.1	Rhodobacterales bacterium HTCC2255			Rhodobacterales	Alphaproteobacteria	Proteobacteria	Bacteria
ZP_01449753.1	Rhodobacterales bacterium HTCC2255			Rhodobacterales	Alphaproteobacteria	Proteobacteria	Bacteria
ZP_01012401.1	Rhodobacterales bacterium HTCC2654			Rhodobacterales	Alphaproteobacteria	Proteobacteria	Bacteria

Accession	species	genus	order	classe	family	phylum	domain
EDZ45255.1	Rhodobacterales bacterium Y4I			Rhodobacterales	Alphaproteobacteria	Proteobacteria	Bacteria
YP_345557.1	Rhodococcus erythropolis	Rhodococcus	Nocardiaceae	Actinomycetales	Actinobacteria (class)	Actinobacteria	Bacteria
YP_707487.1	Rhodococcus jostii	Rhodococcus	Nocardiaceae	Actinomycetales	Actinobacteria (class)	Actinobacteria	Bacteria
YP_704173.1	Rhodococcus jostii	Rhodococcus	Nocardiaceae	Actinomycetales	Actinobacteria (class)	Actinobacteria	Bacteria
YP_706379.1	Rhodococcus jostii	Rhodococcus	Nocardiaceae	Actinomycetales	Actinobacteria (class)	Actinobacteria	Bacteria
YP_522942.1	Rhodoferax ferrireducens	Rhodoferax	Comamonadaceae	Burkholderiales	Betaproteobacteria	Proteobacteria	Bacteria
YP_523187.1	Rhodoferax ferrireducens	Rhodoferax	Comamonadaceae	Burkholderiales	Betaproteobacteria	Proteobacteria	Bacteria
YP_525356.1	Rhodoferax ferrireducens	Rhodoferax	Comamonadaceae	Burkholderiales	Betaproteobacteria	Proteobacteria	Bacteria
YP_523143.1	Rhodoferax ferrireducens	Rhodoferax	Comamonadaceae	Burkholderiales	Betaproteobacteria	Proteobacteria	Bacteria
NP_866086.1	Rhodopirellula baltica	Rhodopirellula	Planctomycetaceae	Planctomycetales	Planctomycetacia	Planctomycetes	Bacteria
NP_870646.1	Rhodopirellula baltica	Rhodopirellula	Planctomycetaceae	Planctomycetales	Planctomycetacia	Planctomycetes	Bacteria
NP_867199.1	Rhodopirellula baltica	Rhodopirellula	Planctomycetaceae	Planctomycetales	Planctomycetacia	Planctomycetes	Bacteria
YP_001989933.1	Rhodopseudomonas palustris	Rhodopseudomonas	Bradyrhizobiaceae	Rhizobiales	Alphaproteobacteria	Proteobacteria	Bacteria
NP_946185.1	Rhodopseudomonas palustris	Rhodopseudomonas	Bradyrhizobiaceae	Rhizobiales	Alphaproteobacteria	Proteobacteria	Bacteria
YP_534629.1	Rhodopseudomonas palustris	Rhodopseudomonas	Bradyrhizobiaceae	Rhizobiales	Alphaproteobacteria	Proteobacteria	Bacteria
YP_783651.1	Rhodopseudomonas palustris	Rhodopseudomonas	Bradyrhizobiaceae	Rhizobiales	Alphaproteobacteria	Proteobacteria	Bacteria
YP_567954.1	Rhodopseudomonas palustris	Rhodopseudomonas	Bradyrhizobiaceae	Rhizobiales	Alphaproteobacteria	Proteobacteria	Bacteria
YP_488181.1	Rhodopseudomonas palustris	Rhodopseudomonas	Bradyrhizobiaceae	Rhizobiales	Alphaproteobacteria	Proteobacteria	Bacteria
YP_567735.1	Rhodopseudomonas palustris	Rhodopseudomonas	Bradyrhizobiaceae	Rhizobiales	Alphaproteobacteria	Proteobacteria	Bacteria
YP_483863.1	Rhodopseudomonas palustris	Rhodopseudomonas	Bradyrhizobiaceae	Rhizobiales	Alphaproteobacteria	Proteobacteria	Bacteria
YP_533702.1	Rhodopseudomonas palustris	Rhodopseudomonas	Bradyrhizobiaceae	Rhizobiales	Alphaproteobacteria	Proteobacteria	Bacteria
YP_782773.1	Rhodopseudomonas palustris	Rhodopseudomonas	Bradyrhizobiaceae	Rhizobiales	Alphaproteobacteria	Proteobacteria	Bacteria
YP_483778.1	Rhodopseudomonas palustris	Rhodopseudomonas	Bradyrhizobiaceae	Rhizobiales	Alphaproteobacteria	Proteobacteria	Bacteria
YP_001989058.1	Rhodopseudomonas palustris	Rhodopseudomonas	Bradyrhizobiaceae	Rhizobiales	Alphaproteobacteria	Proteobacteria	Bacteria
NP_945375.1	Rhodopseudomonas palustris	Rhodopseudomonas	Bradyrhizobiaceae	Rhizobiales	Alphaproteobacteria	Proteobacteria	Bacteria
YP_567869.1	Rhodopseudomonas palustris	Rhodopseudomonas	Bradyrhizobiaceae	Rhizobiales	Alphaproteobacteria	Proteobacteria	Bacteria
YP_483640.1	Rhodopseudomonas palustris	Rhodopseudomonas	Bradyrhizobiaceae	Rhizobiales	Alphaproteobacteria	Proteobacteria	Bacteria
YP_529913.1	Rhodopseudomonas palustris	Rhodopseudomonas	Bradyrhizobiaceae	Rhizobiales	Alphaproteobacteria	Proteobacteria	Bacteria
YP_778959.1	Rhodopseudomonas palustris	Rhodopseudomonas	Bradyrhizobiaceae	Rhizobiales	Alphaproteobacteria	Proteobacteria	Bacteria
YP_001990646.1	Rhodopseudomonas palustris	Rhodopseudomonas	Bradyrhizobiaceae	Rhizobiales	Alphaproteobacteria	Proteobacteria	Bacteria
NP_946806.1	Rhodopseudomonas palustris	Rhodopseudomonas	Bradyrhizobiaceae	Rhizobiales	Alphaproteobacteria	Proteobacteria	Bacteria
YP_779559.1	Rhodopseudomonas palustris	Rhodopseudomonas	Bradyrhizobiaceae	Rhizobiales	Alphaproteobacteria	Proteobacteria	Bacteria
YP_002299792.1	Rhodospirillum centenum	Rhodospirillum	Rhodospirillaceae	Rhodospirillales	Alphaproteobacteria	Proteobacteria	Bacteria
YP_002297025.1	Rhodospirillum centenum	Rhodospirillum	Rhodospirillaceae	Rhodospirillales	Alphaproteobacteria	Proteobacteria	Bacteria
YP_002297500.1	Rhodospirillum centenum	Rhodospirillum	Rhodospirillaceae	Rhodospirillales	Alphaproteobacteria	Proteobacteria	Bacteria
YP_002300129.1	Rhodospirillum centenum	Rhodospirillum	Rhodospirillaceae	Rhodospirillales	Alphaproteobacteria	Proteobacteria	Bacteria
YP_428413.1	Rhodospirillum rubrum	Rhodospirillum	Rhodospirillaceae	Rhodospirillales	Alphaproteobacteria	Proteobacteria	Bacteria

CAC08532.1	Rhodothermus marinus	Rhodothermus	Rhodothermaceae	Sphingobacteriales	Sphingobacteria	Bacteroidetes	Bacteria
AAY23178.1	Rhodothermus marinus	Rhodothermus	Rhodothermaceae	Sphingobacteriales	Sphingobacteria	Bacteroidetes	Bacteria
ZP_02336393.1	Rickettsia africae	Rickettsia	Rickettsiaceae	Rickettsiales	Alphaproteobacteria	Proteobacteria	Bacteria
YP_001493401.1	Rickettsia akari	Rickettsia	Rickettsiaceae	Rickettsiales	Alphaproteobacteria	Proteobacteria	Bacteria
YP_538061.1	Rickettsia bellii	Rickettsia	Rickettsiaceae	Rickettsiales	Alphaproteobacteria	Proteobacteria	Bacteria
YP_001495898.1	Rickettsia bellii	Rickettsia	Rickettsiaceae	Rickettsiales	Alphaproteobacteria	Proteobacteria	Bacteria
YP_001492392.1	Rickettsia canadensis	Rickettsia	Rickettsiaceae	Rickettsiales	Alphaproteobacteria	Proteobacteria	Bacteria
NP_360190.1	Rickettsia conorii	Rickettsia	Rickettsiaceae	Rickettsiales	Alphaproteobacteria	Proteobacteria	Bacteria
YP_246643.1	Rickettsia felis	Rickettsia	Rickettsiaceae	Rickettsiales	Alphaproteobacteria	Proteobacteria	Bacteria
YP_001499310.1	Rickettsia massiliae	Rickettsia	Rickettsiaceae	Rickettsiales	Alphaproteobacteria	Proteobacteria	Bacteria
NP_220786.1	Rickettsia prowazekii	Rickettsia	Rickettsiaceae	Rickettsiales	Alphaproteobacteria	Proteobacteria	Bacteria
YP_001649930.1	Rickettsia rickettsii	Rickettsia	Rickettsiaceae	Rickettsiales	Alphaproteobacteria	Proteobacteria	Bacteria
YP_001494671.1	Rickettsia rickettsii	Rickettsia	Rickettsiaceae	Rickettsiales	Alphaproteobacteria	Proteobacteria	Bacteria
ZP_00142009.1	Rickettsia sibirica	Rickettsia	Rickettsiaceae	Rickettsiales	Alphaproteobacteria	Proteobacteria	Bacteria
ZP_02062292.1	Rickettsiella grylli	Rickettsiella	Coxiellaceae	Legionellales	Gammaproteobacteria	Proteobacteria	Bacteria

Accession	species	genus	order	classe	family	phylum	domain
ZP_01119437.1	Robiginitalea biformata	Robiginitalea	Flavobacteriaceae	Flavobacteriales	Flavobacteria	Bacteroidetes	Bacteria
ZP_01119531.1	Robiginitalea biformata	Robiginitalea	Flavobacteriaceae	Flavobacteriales	Flavobacteria	Bacteroidetes	Bacteria
YP_001433624.1	Roseiflexus castenholzii	Roseiflexus	Chloroflexaceae	Chloroflexales	Chloroflexi (class)	Chloroflexi	Bacteria
YP_001431686.1	Roseiflexus castenholzii	Roseiflexus	Chloroflexaceae	Chloroflexales	Chloroflexi (class)	Chloroflexi	Bacteria
YP_001276593.1	Roseiflexus sp. RS-1	Roseiflexus	Chloroflexaceae	Chloroflexales	Chloroflexi (class)	Chloroflexi	Bacteria
YP_001275293.1	Roseiflexus sp. RS-1	Roseiflexus	Chloroflexaceae	Chloroflexales	Chloroflexi (class)	Chloroflexi	Bacteria
YP_682261.1	Roseobacter denitrificans	Roseobacter	Rhodobacteraceae	Rhodobacterales	Alphaproteobacteria	Proteobacteria	Bacteria
YP_681602.1	Roseobacter denitrificans	Roseobacter	Rhodobacteraceae	Rhodobacterales	Alphaproteobacteria	Proteobacteria	Bacteria
YP_681875.1	Roseobacter denitrificans	Roseobacter	Rhodobacteraceae	Rhodobacterales	Alphaproteobacteria	Proteobacteria	Bacteria
BAB84303.1	Roseobacter denitrificans	Roseobacter	Rhodobacteraceae	Rhodobacterales	Alphaproteobacteria	Proteobacteria	Bacteria
ZP_02140791.1	Roseobacter litoralis	Roseobacter	Rhodobacteraceae	Rhodobacterales	Alphaproteobacteria	Proteobacteria	Bacteria
ZP_02141397.1	Roseobacter litoralis	Roseobacter	Rhodobacteraceae	Rhodobacterales	Alphaproteobacteria	Proteobacteria	Bacteria
ZP_02141655.1	Roseobacter litoralis	Roseobacter	Rhodobacteraceae	Rhodobacterales	Alphaproteobacteria	Proteobacteria	Bacteria
ZP_01904953.1	Roseobacter sp. AzwK-3b	Roseobacter	Rhodobacteraceae	Rhodobacterales	Alphaproteobacteria	Proteobacteria	Bacteria
ZP_01751185.1	Roseobacter sp. CCS2	Roseobacter	Rhodobacteraceae	Rhodobacterales	Alphaproteobacteria	Proteobacteria	Bacteria
ZP_01754686.1	Roseobacter sp. SK209-2-6	Roseobacter	Rhodobacteraceae	Rhodobacterales	Alphaproteobacteria	Proteobacteria	Bacteria
ZP_00959150.1	Roseovarius nubinhibens	Roseovarius	Rhodobacteraceae	Rhodobacterales	Alphaproteobacteria	Proteobacteria	Bacteria
ZP_00959081.1	Roseovarius nubinhibens	Roseovarius	Rhodobacteraceae	Rhodobacterales	Alphaproteobacteria	Proteobacteria	Bacteria
ZP_01038050.1	Roseovarius sp. 217	Roseovarius	Rhodobacteraceae	Rhodobacterales	Alphaproteobacteria	Proteobacteria	Bacteria
ZP_01445618.1	Roseovarius sp. HTCC2601	Roseovarius	Rhodobacteraceae	Rhodobacterales	Alphaproteobacteria	Proteobacteria	Bacteria
ZP_01446254.1	Roseovarius sp. HTCC2601	Roseovarius	Rhodobacteraceae	Rhodobacterales	Alphaproteobacteria	Proteobacteria	Bacteria
ZP_01445338.1	Roseovarius sp. HTCC2601	Roseovarius	Rhodobacteraceae	Rhodobacterales	Alphaproteobacteria	Proteobacteria	Bacteria
ZP_01444008.1	Roseovarius sp. HTCC2601	Roseovarius	Rhodobacteraceae	Rhodobacterales	Alphaproteobacteria	Proteobacteria	Bacteria

AAV66315.1	Rubrivivax gelatinosus	Rubrivivax		Burkholderiales	Betaproteobacteria	Proteobacteria	Bacteria
AAW66132.1	Rubrivivax gelatinosus	Rubrivivax		Burkholderiales	Betaproteobacteria	Proteobacteria	Bacteria
YP_166625.1	Ruegeria pomeroyi	Ruegeria	Rhodobacteraceae	Rhodobacterales	Alphaproteobacteria	Proteobacteria	Bacteria
YP_168721.1	Ruegeria pomeroyi	Ruegeria	Rhodobacteraceae	Rhodobacterales	Alphaproteobacteria	Proteobacteria	Bacteria
YP_165019.1	Ruegeria pomeroyi	Ruegeria	Rhodobacteraceae	Rhodobacterales	Alphaproteobacteria	Proteobacteria	Bacteria
YP_165045.1	Ruegeria pomeroyi	Ruegeria	Rhodobacteraceae	Rhodobacterales	Alphaproteobacteria	Proteobacteria	Bacteria
YP_614285.1	Ruegeria sp. TM1040	Ruegeria	Rhodobacteraceae	Rhodobacterales	Alphaproteobacteria	Proteobacteria	Bacteria
YP_614279.1	Ruegeria sp. TM1040	Ruegeria	Rhodobacteraceae	Rhodobacterales	Alphaproteobacteria	Proteobacteria	Bacteria
YP_614539.1	Ruegeria sp. TM1040	Ruegeria	Rhodobacteraceae	Rhodobacterales	Alphaproteobacteria	Proteobacteria	Bacteria
YP_525513.1	Saccharophagus degradans	Saccharophagus	Alteromonadaceae	Alteromonadales	Gammaproteobacteria	Proteobacteria	Bacteria
YP_527894.1	Saccharophagus degradans	Saccharophagus	Alteromonadaceae	Alteromonadales	Gammaproteobacteria	Proteobacteria	Bacteria
YP_001109127.1	Saccharopolyspora erythraea	Saccharopolyspora	Pseudonocardiaceae	Actinomycetales	Actinobacteria (class)	Actinobacteria	Bacteria
YP_001103438.1	Saccharopolyspora erythraea	Saccharopolyspora	Pseudonocardiaceae	Actinomycetales	Actinobacteria (class)	Actinobacteria	Bacteria
ZP_01744693.1	Sagittula stellata	Sagittula	Rhodobacteraceae	Rhodobacterales	Alphaproteobacteria	Proteobacteria	Bacteria
ZP_01746009.1	Sagittula stellata	Sagittula	Rhodobacteraceae	Rhodobacterales	Alphaproteobacteria	Proteobacteria	Bacteria
ZP_01747947.1	Sagittula stellata	Sagittula	Rhodobacteraceae	Rhodobacterales	Alphaproteobacteria	Proteobacteria	Bacteria
ZP_01746085.1	Sagittula stellata	Sagittula	Rhodobacteraceae	Rhodobacterales	Alphaproteobacteria	Proteobacteria	Bacteria
YP_446205.1	Salinibacter ruber	Salinibacter	Rhodothermaceae	Sphingobacteriales	Sphingobacteria	Bacteroidetes	Bacteria
YP_444460.1	Salinibacter ruber	Salinibacter	Rhodothermaceae	Sphingobacteriales	Sphingobacteria	Bacteroidetes	Bacteria
YP_444468.1	Salinibacter ruber	Salinibacter	Rhodothermaceae	Sphingobacteriales	Sphingobacteria	Bacteroidetes	Bacteria
YP_001537144.1	Salinispora arenicola	Salinispora	Micromonosporaceae	Actinomycetales	Actinobacteria (class)	Actinobacteria	Bacteria
YP_001535885.1	Salinispora arenicola	Salinispora	Micromonosporaceae	Actinomycetales	Actinobacteria (class)	Actinobacteria	Bacteria

Accession	species	genus	order	classe	family	phylum	domain
YP_001158972.1	Salinispora tropica	Salinispora	Micromonosporaceae	Actinomycetales	Actinobacteria (class)	Actinobacteria	Bacteria
YP_001157943.1	Salinispora tropica	Salinispora	Micromonosporaceae	Actinomycetales	Actinobacteria (class)	Actinobacteria	Bacteria
YP_002242573.1	Salmonella enterica	Salmonella	Enterobacteriaceae	Enterobacteriales	Gammaproteobacteria	Proteobacteria	Bacteria
YP_002214396.1	Salmonella enterica	Salmonella	Enterobacteriaceae	Enterobacteriales	Gammaproteobacteria	Proteobacteria	Bacteria
YP_002145423.1	Salmonella enterica	Salmonella	Enterobacteriaceae	Enterobacteriales	Gammaproteobacteria	Proteobacteria	Bacteria
YP_002225554.1	Salmonella enterica	Salmonella	Enterobacteriaceae	Enterobacteriales	Gammaproteobacteria	Proteobacteria	Bacteria
YP_002039684.1	Salmonella enterica	Salmonella	Enterobacteriaceae	Enterobacteriales	Gammaproteobacteria	Proteobacteria	Bacteria
YP_002044477.1	Salmonella enterica	Salmonella	Enterobacteriaceae	Enterobacteriales	Gammaproteobacteria	Proteobacteria	Bacteria
YP_002113473.1	Salmonella enterica	Salmonella	Enterobacteriaceae	Enterobacteriales	Gammaproteobacteria	Proteobacteria	Bacteria
NP_806148.1	Salmonella enterica	Salmonella	Enterobacteriaceae	Enterobacteriales	Gammaproteobacteria	Proteobacteria	Bacteria
NP_455039.1	Salmonella enterica	Salmonella	Enterobacteriaceae	Enterobacteriales	Gammaproteobacteria	Proteobacteria	Bacteria
NP_459438.1	Salmonella enterica	Salmonella	Enterobacteriaceae	Enterobacteriales	Gammaproteobacteria	Proteobacteria	Bacteria
YP_151477.1	Salmonella enterica	Salmonella	Enterobacteriaceae	Enterobacteriales	Gammaproteobacteria	Proteobacteria	Bacteria
YP_215471.1	Salmonella enterica	Salmonella	Enterobacteriaceae	Enterobacteriales	Gammaproteobacteria	Proteobacteria	Bacteria
YP_001589333.1	Salmonella enterica	Salmonella	Enterobacteriaceae	Enterobacteriales	Gammaproteobacteria	Proteobacteria	Bacteria
YP_001571493.1	Salmonella enterica	Salmonella	Enterobacteriaceae	Enterobacteriales	Gammaproteobacteria	Proteobacteria	Bacteria

YP_001477321.1	Serratia proteamaculans	Serratia	Enterobacteriaceae	Enterobacteriales	Gammaproteobacteria	Proteobacteria	Bacteria
YP_929356.1	Shewanella amazonensis	Shewanella	Shewanellaceae	Alteromonadales	Gammaproteobacteria	Proteobacteria	Bacteria
YP_927666.1	Shewanella amazonensis	Shewanella	Shewanellaceae	Alteromonadales	Gammaproteobacteria	Proteobacteria	Bacteria
YP_926606.1	Shewanella amazonensis	Shewanella	Shewanellaceae	Alteromonadales	Gammaproteobacteria	Proteobacteria	Bacteria
YP_002360007.1	Shewanella baltica	Shewanella	Shewanellaceae	Alteromonadales	Gammaproteobacteria	Proteobacteria	Bacteria
YP_001048556.1	Shewanella baltica	Shewanella	Shewanellaceae	Alteromonadales	Gammaproteobacteria	Proteobacteria	Bacteria
YP_001556730.1	Shewanella baltica	Shewanella	Shewanellaceae	Alteromonadales	Gammaproteobacteria	Proteobacteria	Bacteria
YP_001368360.1	Shewanella baltica	Shewanella	Shewanellaceae	Alteromonadales	Gammaproteobacteria	Proteobacteria	Bacteria
YP_002360041.1	Shewanella baltica	Shewanella	Shewanellaceae	Alteromonadales	Gammaproteobacteria	Proteobacteria	Bacteria
YP_001368398.1	Shewanella baltica	Shewanella	Shewanellaceae	Alteromonadales	Gammaproteobacteria	Proteobacteria	Bacteria
YP_001556769.1	Shewanella baltica	Shewanella	Shewanellaceae	Alteromonadales	Gammaproteobacteria	Proteobacteria	Bacteria
YP_002358136.1	Shewanella baltica	Shewanella	Shewanellaceae	Alteromonadales	Gammaproteobacteria	Proteobacteria	Bacteria
YP_001041605.1	Shewanella baltica	Shewanella	Shewanellaceae	Alteromonadales	Gammaproteobacteria	Proteobacteria	Bacteria
YP_001554649.1	Shewanella baltica	Shewanella	Shewanellaceae	Alteromonadales	Gammaproteobacteria	Proteobacteria	Bacteria
YP_001050566.1	Shewanella baltica	Shewanella	Shewanellaceae	Alteromonadales	Gammaproteobacteria	Proteobacteria	Bacteria
YP_001366375.1	Shewanella baltica	Shewanella	Shewanellaceae	Alteromonadales	Gammaproteobacteria	Proteobacteria	Bacteria
ZP_02156063.1	Shewanella benthica	Shewanella	Shewanellaceae	Alteromonadales	Gammaproteobacteria	Proteobacteria	Bacteria
ZP_02158631.1	Shewanella benthica	Shewanella	Shewanellaceae	Alteromonadales	Gammaproteobacteria	Proteobacteria	Bacteria
ZP_02156284.1	Shewanella benthica	Shewanella	Shewanellaceae	Alteromonadales	Gammaproteobacteria	Proteobacteria	Bacteria
YP_564516.1	Shewanella denitrificans	Shewanella	Shewanellaceae	Alteromonadales	Gammaproteobacteria	Proteobacteria	Bacteria
YP_562858.1	Shewanella denitrificans	Shewanella	Shewanellaceae	Alteromonadales	Gammaproteobacteria	Proteobacteria	Bacteria
YP_562986.1	Shewanella denitrificans	Shewanella	Shewanellaceae	Alteromonadales	Gammaproteobacteria	Proteobacteria	Bacteria
YP_748958.1	Shewanella frigidimarina	Shewanella	Shewanellaceae	Alteromonadales	Gammaproteobacteria	Proteobacteria	Bacteria
YP_748924.1	Shewanella frigidimarina	Shewanella	Shewanellaceae	Alteromonadales	Gammaproteobacteria	Proteobacteria	Bacteria
YP_750694.1	Shewanella frigidimarina	Shewanella	Shewanellaceae	Alteromonadales	Gammaproteobacteria	Proteobacteria	Bacteria
YP_751928.1	Shewanella frigidimarina	Shewanella	Shewanellaceae	Alteromonadales	Gammaproteobacteria	Proteobacteria	Bacteria
YP_001672492.1	Shewanella halifaxensis	Shewanella	Shewanellaceae	Alteromonadales	Gammaproteobacteria	Proteobacteria	Bacteria
YP_001674415.1	Shewanella halifaxensis	Shewanella	Shewanellaceae	Alteromonadales	Gammaproteobacteria	Proteobacteria	Bacteria
YP_001675756.1	Shewanella halifaxensis	Shewanella	Shewanellaceae	Alteromonadales	Gammaproteobacteria	Proteobacteria	Bacteria

Accession	species	genus	order	classe	family	phylum	domain
YP_001092255.1	Shewanella loihica	Shewanella	Shewanellaceae	Alteromonadales	Gammaproteobacteria	Proteobacteria	Bacteria
YP_001092498.1	Shewanella loihica	Shewanella	Shewanellaceae	Alteromonadales	Gammaproteobacteria	Proteobacteria	Bacteria
YP_001094110.1	Shewanella loihica	Shewanella	Shewanellaceae	Alteromonadales	Gammaproteobacteria	Proteobacteria	Bacteria
YP_001092788.1	Shewanella loihica	Shewanella	Shewanellaceae	Alteromonadales	Gammaproteobacteria	Proteobacteria	Bacteria
NP_720123.1	Shewanella oneidensis	Shewanella	Shewanellaceae	Alteromonadales	Gammaproteobacteria	Proteobacteria	Bacteria
NP_717954.1	Shewanella oneidensis	Shewanella	Shewanellaceae	Alteromonadales	Gammaproteobacteria	Proteobacteria	Bacteria
YP_001503845.1	Shewanella pealeana	Shewanella	Shewanellaceae	Alteromonadales	Gammaproteobacteria	Proteobacteria	Bacteria
YP_001502069.1	Shewanella pealeana	Shewanella	Shewanellaceae	Alteromonadales	Gammaproteobacteria	Proteobacteria	Bacteria

Accession	species	genus	order	classe	family	phylum	domain
YP_001500817.1	Shewanella pealeana	Shewanella	Shewanellaceae	Alteromonadales	Gammaproteobacteria	Proteobacteria	Bacteria
YP_002314036.1	Shewanella piezotolerans	Shewanella	Shewanellaceae	Alteromonadales	Gammaproteobacteria	Proteobacteria	Bacteria
YP_002310796.1	Shewanella piezotolerans	Shewanella	Shewanellaceae	Alteromonadales	Gammaproteobacteria	Proteobacteria	Bacteria
YP_002311898.1	Shewanella piezotolerans	Shewanella	Shewanellaceae	Alteromonadales	Gammaproteobacteria	Proteobacteria	Bacteria
YP_002313394.1	Shewanella piezotolerans	Shewanella	Shewanellaceae	Alteromonadales	Gammaproteobacteria	Proteobacteria	Bacteria
YP_002310231.1	Shewanella piezotolerans	Shewanella	Shewanellaceae	Alteromonadales	Gammaproteobacteria	Proteobacteria	Bacteria
YP_001185289.1	Shewanella putrefaciens	Shewanella	Shewanellaceae	Alteromonadales	Gammaproteobacteria	Proteobacteria	Bacteria
ZP_01706832.1	Shewanella putrefaciens	Shewanella	Shewanellaceae	Alteromonadales	Gammaproteobacteria	Proteobacteria	Bacteria
ZP_01706816.1	Shewanella putrefaciens	Shewanella	Shewanellaceae	Alteromonadales	Gammaproteobacteria	Proteobacteria	Bacteria
YP_001185364.1	Shewanella putrefaciens	Shewanella	Shewanellaceae	Alteromonadales	Gammaproteobacteria	Proteobacteria	Bacteria
YP_001183481.1	Shewanella putrefaciens	Shewanella	Shewanellaceae	Alteromonadales	Gammaproteobacteria	Proteobacteria	Bacteria
ZP_01705821.1	Shewanella putrefaciens	Shewanella	Shewanellaceae	Alteromonadales	Gammaproteobacteria	Proteobacteria	Bacteria
YP_001184584.1	Shewanella putrefaciens	Shewanella	Shewanellaceae	Alteromonadales	Gammaproteobacteria	Proteobacteria	Bacteria
ZP_01705890.1	Shewanella putrefaciens	Shewanella	Shewanellaceae	Alteromonadales	Gammaproteobacteria	Proteobacteria	Bacteria
YP_001471938.1	Shewanella sediminis	Shewanella	Shewanellaceae	Alteromonadales	Gammaproteobacteria	Proteobacteria	Bacteria
YP_001473948.1	Shewanella sediminis	Shewanella	Shewanellaceae	Alteromonadales	Gammaproteobacteria	Proteobacteria	Bacteria
YP_001475554.1	Shewanella sediminis	Shewanella	Shewanellaceae	Alteromonadales	Gammaproteobacteria	Proteobacteria	Bacteria
YP_871616.1	Shewanella sp. ANA-3	Shewanella	Shewanellaceae	Alteromonadales	Gammaproteobacteria	Proteobacteria	Bacteria
YP_871655.1	Shewanella sp. ANA-3	Shewanella	Shewanellaceae	Alteromonadales	Gammaproteobacteria	Proteobacteria	Bacteria
YP_869743.1	Shewanella sp. ANA-3	Shewanella	Shewanellaceae	Alteromonadales	Gammaproteobacteria	Proteobacteria	Bacteria
YP_868490.1	Shewanella sp. ANA-3	Shewanella	Shewanellaceae	Alteromonadales	Gammaproteobacteria	Proteobacteria	Bacteria
YP_735912.1	Shewanella sp. MR-4	Shewanella	Shewanellaceae	Alteromonadales	Gammaproteobacteria	Proteobacteria	Bacteria
YP_735944.1	Shewanella sp. MR-4	Shewanella	Shewanellaceae	Alteromonadales	Gammaproteobacteria	Proteobacteria	Bacteria
YP_734136.1	Shewanella sp. MR-4	Shewanella	Shewanellaceae	Alteromonadales	Gammaproteobacteria	Proteobacteria	Bacteria
YP_735217.1	Shewanella sp. MR-4	Shewanella	Shewanellaceae	Alteromonadales	Gammaproteobacteria	Proteobacteria	Bacteria
YP_739899.1	Shewanella sp. MR-7	Shewanella	Shewanellaceae	Alteromonadales	Gammaproteobacteria	Proteobacteria	Bacteria
YP_739949.1	Shewanella sp. MR-7	Shewanella	Shewanellaceae	Alteromonadales	Gammaproteobacteria	Proteobacteria	Bacteria
YP_738015.1	Shewanella sp. MR-7	Shewanella	Shewanellaceae	Alteromonadales	Gammaproteobacteria	Proteobacteria	Bacteria
YP_736939.1	Shewanella sp. MR-7	Shewanella	Shewanellaceae	Alteromonadales	Gammaproteobacteria	Proteobacteria	Bacteria
YP_961539.1	Shewanella sp. W3-18-1	Shewanella	Shewanellaceae	Alteromonadales	Gammaproteobacteria	Proteobacteria	Bacteria
YP_961501.1	Shewanella sp. W3-18-1	Shewanella	Shewanellaceae	Alteromonadales	Gammaproteobacteria	Proteobacteria	Bacteria
YP_963429.1	Shewanella sp. W3-18-1	Shewanella	Shewanellaceae	Alteromonadales	Gammaproteobacteria	Proteobacteria	Bacteria
YP_962277.1	Shewanella sp. W3-18-1	Shewanella	Shewanellaceae	Alteromonadales	Gammaproteobacteria	Proteobacteria	Bacteria
BAA94865.1	Shewanella violacea	Shewanella	Shewanellaceae	Alteromonadales	Gammaproteobacteria	Proteobacteria	Bacteria
YP_001758573.1	Shewanella woodyi	Shewanella	Shewanellaceae	Alteromonadales	Gammaproteobacteria	Proteobacteria	Bacteria
YP_001758825.1	Shewanella woodyi	Shewanella	Shewanellaceae	Alteromonadales	Gammaproteobacteria	Proteobacteria	Bacteria
YP_001760798.1	Shewanella woodyi	Shewanella	Shewanellaceae	Alteromonadales	Gammaproteobacteria	Proteobacteria	Bacteria

YP_001759151.1	Shewanella woodyi	Shewanella	Shewanellaceae	Alteromonadales	Gammaproteobacteria	Proteobacteria	Bacteria
YP_001879141.1	Shigella boydii	Shigella	Enterobacteriaceae	Enterobacteriales	Gammaproteobacteria	Proteobacteria	Bacteria
YP_406866.1	Shigella boydii	Shigella	Enterobacteriaceae	Enterobacteriales	Gammaproteobacteria	Proteobacteria	Bacteria
YP_309425.1	Shigella sonnei	Shigella	Enterobacteriaceae	Enterobacteriales	Gammaproteobacteria	Proteobacteria	Bacteria
YP_001326210.1	Sinorhizobium medicae	Sinorhizobium	Rhizobiaceae	Rhizobiales	Alphaproteobacteria	Proteobacteria	Bacteria
YP_001328093.1	Sinorhizobium medicae	Sinorhizobium	Rhizobiaceae	Rhizobiales	Alphaproteobacteria	Proteobacteria	Bacteria
YP_001313108.1	Sinorhizobium medicae	Sinorhizobium	Rhizobiaceae	Rhizobiales	Alphaproteobacteria	Proteobacteria	Bacteria
YP_001314880.1	Sinorhizobium medicae	Sinorhizobium	Rhizobiaceae	Rhizobiales	Alphaproteobacteria	Proteobacteria	Bacteria
YP_001313492.1	Sinorhizobium medicae	Sinorhizobium	Rhizobiaceae	Rhizobiales	Alphaproteobacteria	Proteobacteria	Bacteria
YP_001314560.1	Sinorhizobium medicae	Sinorhizobium	Rhizobiaceae	Rhizobiales	Alphaproteobacteria	Proteobacteria	Bacteria
YP_001314551.1	Sinorhizobium medicae	Sinorhizobium	Rhizobiaceae	Rhizobiales	Alphaproteobacteria	Proteobacteria	Bacteria
YP_001314819.1	Sinorhizobium medicae	Sinorhizobium	Rhizobiaceae	Rhizobiales	Alphaproteobacteria	Proteobacteria	Bacteria
NP_385011.1	Sinorhizobium meliloti	Sinorhizobium	Rhizobiaceae	Rhizobiales	Alphaproteobacteria	Proteobacteria	Bacteria
NP_386623.1	Sinorhizobium meliloti	Sinorhizobium	Rhizobiaceae	Rhizobiales	Alphaproteobacteria	Proteobacteria	Bacteria
NP_437856.1	Sinorhizobium meliloti	Sinorhizobium	Rhizobiaceae	Rhizobiales	Alphaproteobacteria	Proteobacteria	Bacteria
NP_437488.1	Sinorhizobium meliloti	Sinorhizobium	Rhizobiaceae	Rhizobiales	Alphaproteobacteria	Proteobacteria	Bacteria
NP_435656.1	Sinorhizobium meliloti	Sinorhizobium	Rhizobiaceae	Rhizobiales	Alphaproteobacteria	Proteobacteria	Bacteria
NP_435566.1	Sinorhizobium meliloti	Sinorhizobium	Rhizobiaceae	Rhizobiales	Alphaproteobacteria	Proteobacteria	Bacteria
NP_435940.1	Sinorhizobium meliloti	Sinorhizobium	Rhizobiaceae	Rhizobiales	Alphaproteobacteria	Proteobacteria	Bacteria
YP_454346.1	Sodalis glossinidius	Sodalis	Enterobacteriaceae	Enterobacteriales	Gammaproteobacteria	Proteobacteria	Bacteria
YP_826822.1	Solibacter usitatus	Solibacter	Solibacteraceae	Solibacterales	Solibacteres	Acidobacteria	Bacteria
YP_828106.1	Solibacter usitatus	Solibacter	Solibacteraceae	Solibacterales	Solibacteres	Acidobacteria	Bacteria
YP_828774.1	Solibacter usitatus	Solibacter	Solibacteraceae	Solibacterales	Solibacteres	Acidobacteria	Bacteria
YP_821793.1	Solibacter usitatus	Solibacter	Solibacteraceae	Solibacterales	Solibacteres	Acidobacteria	Bacteria
YP_824765.1	Solibacter usitatus	Solibacter	Solibacteraceae	Solibacterales	Solibacteres	Acidobacteria	Bacteria
YP_824206.1	Solibacter usitatus	Solibacter	Solibacteraceae	Solibacterales	Solibacteres	Acidobacteria	Bacteria
YP_001618242.1	Sorangium cellulosum	Sorangium	Polyangiaceae	Myxococcales	Deltaproteobacteria	Proteobacteria	Bacteria
YP_001615167.1	Sorangium cellulosum	Sorangium	Polyangiaceae	Myxococcales	Deltaproteobacteria	Proteobacteria	Bacteria
YP_001613920.1	Sorangium cellulosum	Sorangium	Polyangiaceae	Myxococcales	Deltaproteobacteria	Proteobacteria	Bacteria
YP_001619234.1	Sorangium cellulosum	Sorangium	Polyangiaceae	Myxococcales	Deltaproteobacteria	Proteobacteria	Bacteria
AAR05968.1	Sphingobium indicum	Sphingobium	Sphingomonadaceae	Sphingomonadales	Alphaproteobacteria	Proteobacteria	Bacteria
ZP_01302641.1	Sphingomonas sp. SKA58	Sphingomonas	Sphingomonadaceae	Sphingomonadales	Alphaproteobacteria	Proteobacteria	Bacteria
ZP_01305192.1	Sphingomonas sp. SKA58	Sphingomonas	Sphingomonadaceae	Sphingomonadales	Alphaproteobacteria	Proteobacteria	Bacteria
ZP_01301620.1	Sphingomonas sp. SKA58	Sphingomonas	Sphingomonadaceae	Sphingomonadales	Alphaproteobacteria	Proteobacteria	Bacteria
ZP_01301694.1	Sphingomonas sp. SKA58	Sphingomonas	Sphingomonadaceae	Sphingomonadales	Alphaproteobacteria	Proteobacteria	Bacteria
ZP_01302542.1	Sphingomonas sp. SKA58	Sphingomonas	Sphingomonadaceae	Sphingomonadales	Alphaproteobacteria	Proteobacteria	Bacteria
YP_001264359.1	Sphingomonas wittichii	Sphingomonas	Sphingomonadaceae	Sphingomonadales	Alphaproteobacteria	Proteobacteria	Bacteria
YP_001262300.1	Sphingomonas wittichii	Sphingomonas	Sphingomonadaceae	Sphingomonadales	Alphaproteobacteria	Proteobacteria	Bacteria
YP_001260078.1	Sphingomonas wittichii	Sphingomonas	Sphingomonadaceae	Sphingomonadales	Alphaproteobacteria	Proteobacteria	Bacteria
YP_001265090.1	Sphingomonas wittichii	Sphingomonas	Sphingomonadaceae	Sphingomonadales	Alphaproteobacteria	Proteobacteria	Bacteria
YP_616451.1	Sphingopyxis alaskensis	Sphingopyxis	Sphingomonadaceae	Sphingomonadales	Alphaproteobacteria	Proteobacteria	Bacteria
YP_616553.1	Sphingopyxis alaskensis	Sphingopyxis	Sphingomonadaceae	Sphingomonadales	Alphaproteobacteria	Proteobacteria	Bacteria
YP_616722.1	Sphingopyxis alaskensis	Sphingopyxis	Sphingomonadaceae	Sphingomonadales	Alphaproteobacteria	Proteobacteria	Bacteria

YP_040448.1	Staphylococcus aureus	Staphylococcus	Staphylococcaceae	Bacillales	Bacilli	Firmicutes	Bacteria
YP_039724.1	Staphylococcus aureus	Staphylococcus	Staphylococcaceae	Bacillales	Bacilli	Firmicutes	Bacteria

Accession	species	genus	order	classe	family	phylum	domain
YP_001246495.1	Staphylococcus aureus	Staphylococcus	Staphylococcaceae	Bacillales	Bacilli	Firmicutes	Bacteria
YP_001331963.1	Staphylococcus aureus	Staphylococcus	Staphylococcaceae	Bacillales	Bacilli	Firmicutes	Bacteria
YP_001574903.1	Staphylococcus aureus	Staphylococcus	Staphylococcaceae	Bacillales	Bacilli	Firmicutes	Bacteria
YP_001441642.1	Staphylococcus aureus	Staphylococcus	Staphylococcaceae	Bacillales	Bacilli	Firmicutes	Bacteria
YP_493660.1	Staphylococcus aureus	Staphylococcus	Staphylococcaceae	Bacillales	Bacilli	Firmicutes	Bacteria
YP_499553.1	Staphylococcus aureus	Staphylococcus	Staphylococcaceae	Bacillales	Bacilli	Firmicutes	Bacteria
YP_043120.1	Staphylococcus aureus	Staphylococcus	Staphylococcaceae	Bacillales	Bacilli	Firmicutes	Bacteria
NP_374179.1	Staphylococcus aureus	Staphylococcus	Staphylococcaceae	Bacillales	Bacilli	Firmicutes	Bacteria
NP_371584.1	Staphylococcus aureus	Staphylococcus	Staphylococcaceae	Bacillales	Bacilli	Firmicutes	Bacteria
YP_416411.1	Staphylococcus aureus	Staphylococcus	Staphylococcaceae	Bacillales	Bacilli	Firmicutes	Bacteria
YP_001316283.1	Staphylococcus aureus	Staphylococcus	Staphylococcaceae	Bacillales	Bacilli	Firmicutes	Bacteria
NP_645760.1	Staphylococcus aureus	Staphylococcus	Staphylococcaceae	Bacillales	Bacilli	Firmicutes	Bacteria
YP_185933.1	Staphylococcus aureus	Staphylococcus	Staphylococcaceae	Bacillales	Bacilli	Firmicutes	Bacteria
YP_188230.1	Staphylococcus epidermidis	Staphylococcus	Staphylococcaceae	Bacillales	Bacilli	Firmicutes	Bacteria
NP_764313.1	Staphylococcus epidermidis	Staphylococcus	Staphylococcaceae	Bacillales	Bacilli	Firmicutes	Bacteria
YP_253817.1	Staphylococcus haemolyticus	Staphylococcus	Staphylococcaceae	Bacillales	Bacilli	Firmicutes	Bacteria
YP_301821.1	Staphylococcus saprophyticus	Staphylococcus	Staphylococcaceae	Bacillales	Bacilli	Firmicutes	Bacteria
YP_002026699.1	Stenotrophomonas maltophilia	Stenotrophomonas	Xanthomonadaceae	Xanthomonadales	Gammaproteobacteria	Proteobacteria	Bacteria
YP_001970342.1	Stenotrophomonas maltophilia	Stenotrophomonas	Xanthomonadaceae	Xanthomonadales	Gammaproteobacteria	Proteobacteria	Bacteria
YP_001971217.1	Stenotrophomonas maltophilia	Stenotrophomonas	Xanthomonadaceae	Xanthomonadales	Gammaproteobacteria	Proteobacteria	Bacteria
YP_002027529.1	Stenotrophomonas maltophilia	Stenotrophomonas	Xanthomonadaceae	Xanthomonadales	Gammaproteobacteria	Proteobacteria	Bacteria
YP_002030176.1	Stenotrophomonas maltophilia	Stenotrophomonas	Xanthomonadaceae	Xanthomonadales	Gammaproteobacteria	Proteobacteria	Bacteria
YP_001974059.1	Stenotrophomonas maltophilia	Stenotrophomonas	Xanthomonadaceae	Xanthomonadales	Gammaproteobacteria	Proteobacteria	Bacteria
CAJ89853.1	Streptomyces ambofaciens	Streptomyces	Streptomycetaceae	Actinomycetales	Actinobacteria (class)	Actinobacteria	Bacteria
NP_827713.1	Streptomyces avermitilis	Streptomyces	Streptomycetaceae	Actinomycetales	Actinobacteria (class)	Actinobacteria	Bacteria
NP_827224.1	Streptomyces avermitilis	Streptomyces	Streptomycetaceae	Actinomycetales	Actinobacteria (class)	Actinobacteria	Bacteria
ZP_03185674.1	Streptomyces clavuligerus	Streptomyces	Streptomycetaceae	Actinomycetales	Actinobacteria (class)	Actinobacteria	Bacteria
ZP_03183291.1	Streptomyces clavuligerus	Streptomyces	Streptomycetaceae	Actinomycetales	Actinobacteria (class)	Actinobacteria	Bacteria
NP_631290.1	Streptomyces coelicolor	Streptomyces	Streptomycetaceae	Actinomycetales	Actinobacteria (class)	Actinobacteria	Bacteria
NP_626411.1	Streptomyces coelicolor	Streptomyces	Streptomycetaceae	Actinomycetales	Actinobacteria (class)	Actinobacteria	Bacteria
YP_001821828.1	Streptomyces griseus	Streptomyces	Streptomycetaceae	Actinomycetales	Actinobacteria (class)	Actinobacteria	Bacteria
YP_001826865.1	Streptomyces griseus	Streptomyces	Streptomycetaceae	Actinomycetales	Actinobacteria (class)	Actinobacteria	Bacteria
YP_002196331.1	Streptomyces pristinaespiralis	Streptomyces	Streptomycetaceae	Actinomycetales	Actinobacteria (class)	Actinobacteria	Bacteria
YP_002198657.1	Streptomyces pristinaespiralis	Streptomyces	Streptomycetaceae	Actinomycetales	Actinobacteria (class)	Actinobacteria	Bacteria
YP_002196596.1	Streptomyces pristinaespiralis	Streptomyces	Streptomycetaceae	Actinomycetales	Actinobacteria (class)	Actinobacteria	Bacteria

Accession	species	genus	order	classe	family	phylum	domain
ZP_03171263.1	Streptomyces sp. Mg1	Streptomyces	Streptomycetaceae	Actinomycetales	Actinobacteria (class)	Actinobacteria	Bacteria
YP_002204627.1	Streptomyces sviceus	Streptomyces	Streptomycetaceae	Actinomycetales	Actinobacteria (class)	Actinobacteria	Bacteria
ZP_03196979.1	Streptomyces sviceus	Streptomyces	Streptomycetaceae	Actinomycetales	Actinobacteria (class)	Actinobacteria	Bacteria
YP_002204028.1	Streptomyces sviceus	Streptomyces	Streptomycetaceae	Actinomycetales	Actinobacteria (class)	Actinobacteria	Bacteria
ZP_00955289.1	Sulfitobacter sp. EE-36	Sulfitobacter	Rhodobacteraceae	Rhodobacterales	Alphaproteobacteria	Proteobacteria	Bacteria
ZP_00956065.1	Sulfitobacter sp. EE-36	Sulfitobacter	Rhodobacteraceae	Rhodobacterales	Alphaproteobacteria	Proteobacteria	Bacteria
ZP_00956569.1	Sulfitobacter sp. EE-36	Sulfitobacter	Rhodobacteraceae	Rhodobacterales	Alphaproteobacteria	Proteobacteria	Bacteria
YP_256842.1	Sulfolobus acidocaldarius	Sulfolobus	Sulfolobaceae	Sulfolobales	Thermoprotei	Crenarchaeota	Archaea
YP_254815.1	Sulfolobus acidocaldarius	Sulfolobus	Sulfolobaceae	Sulfolobales	Thermoprotei	Crenarchaeota	Archaea
YP_256675.1	Sulfolobus acidocaldarius	Sulfolobus	Sulfolobaceae	Sulfolobales	Thermoprotei	Crenarchaeota	Archaea
ABG91823.1	Sulfolobus metallicus	Sulfolobus	Sulfolobaceae	Sulfolobales	Thermoprotei	Crenarchaeota	Archaea
NP_344288.1	Sulfolobus solfataricus	Sulfolobus	Sulfolobaceae	Sulfolobales	Thermoprotei	Crenarchaeota	Archaea
NP_341619.1	Sulfolobus solfataricus	Sulfolobus	Sulfolobaceae	Sulfolobales	Thermoprotei	Crenarchaeota	Archaea
NP_343985.1	Sulfolobus solfataricus	Sulfolobus	Sulfolobaceae	Sulfolobales	Thermoprotei	Crenarchaeota	Archaea
NP_342994.1	Sulfolobus solfataricus	Sulfolobus	Sulfolobaceae	Sulfolobales	Thermoprotei	Crenarchaeota	Archaea
NP_375951.1	Sulfolobus tokodaii	Sulfolobus	Sulfolobaceae	Sulfolobales	Thermoprotei	Crenarchaeota	Archaea
NP_378042.1	Sulfolobus tokodaii	Sulfolobus	Sulfolobaceae	Sulfolobales	Thermoprotei	Crenarchaeota	Archaea
NP_376500.1	Sulfolobus tokodaii	Sulfolobus	Sulfolobaceae	Sulfolobales	Thermoprotei	Crenarchaeota	Archaea
NP_376567.1	Sulfolobus tokodaii	Sulfolobus	Sulfolobaceae	Sulfolobales	Thermoprotei	Crenarchaeota	Archaea
NP_378599.1	Sulfolobus tokodaii	Sulfolobus	Sulfolobaceae	Sulfolobales	Thermoprotei	Crenarchaeota	Archaea
NP_375983.1	Sulfolobus tokodaii	Sulfolobus	Sulfolobaceae	Sulfolobales	Thermoprotei	Crenarchaeota	Archaea
NP_378396.1	Sulfolobus tokodaii	Sulfolobus	Sulfolobaceae	Sulfolobales	Thermoprotei	Crenarchaeota	Archaea
YP_001930359.1	Sulfurihydrogenibium sp. YO3AOP1	Sulfurihydrogenibium	Hydrogenothermaceae	Aquificales	Aquificae (class)	Aquificae	Bacteria
YP_001930667.1	Sulfurihydrogenibium sp. YO3AOP1	Sulfurihydrogenibium	Hydrogenothermaceae	Aquificales	Aquificae (class)	Aquificae	Bacteria
YP_392597.1	Sulfurimonas denitrificans	Sulfurimonas	Helicobacteraceae	Campylobacterales	Epsilonproteobacteria	Proteobacteria	Bacteria
YP_394493.1	Sulfurimonas denitrificans	Sulfurimonas	Helicobacteraceae	Campylobacterales	Epsilonproteobacteria	Proteobacteria	Bacteria
YP_001357495.1	Sulfurovum sp. NBC37-1	Sulfurovum			Epsilonproteobacteria	Proteobacteria	Bacteria
YP_001357561.1	Sulfurovum sp. NBC37-1	Sulfurovum			Epsilonproteobacteria	Proteobacteria	Bacteria
YP_075926.1	Symbiobacterium thermophilum	Symbiobacterium	Clostridiales Family XVIII	Clostridiales	Clostridia	Firmicutes	Bacteria
YP_076979.1	Symbiobacterium thermophilum	Symbiobacterium	Clostridiales Family XVIII	Clostridiales	Clostridia	Firmicutes	Bacteria
YP_401620.1	Synechococcus elongatus	Synechococcus		Chroococcales		Cyanobacteria	Bacteria
YP_172217.1	Synechococcus elongatus	Synechococcus		Chroococcales		Cyanobacteria	Bacteria
YP_172019.1	Synechococcus elongatus	Synechococcus		Chroococcales		Cyanobacteria	Bacteria
YP_399221.1	Synechococcus elongatus	Synechococcus		Chroococcales		Cyanobacteria	Bacteria
YP_731131.1	Synechococcus sp. CC9311	Synechococcus		Chroococcales		Cyanobacteria	Bacteria
YP_731328.1	Synechococcus sp. CC9311	Synechococcus		Chroococcales		Cyanobacteria	Bacteria
YP_381293.1	Synechococcus sp. CC9605	Synechococcus		Chroococcales		Cyanobacteria	Bacteria

YP_380936.1	Synechococcus sp. CC9605	Synechococcus		Chroococcales		Cyanobacteria	Bacteria
YP_377756.1	Synechococcus sp. CC9902	Synechococcus		Chroococcales		Cyanobacteria	Bacteria
YP_478886.1	Synechococcus sp. JA-2-3B'a(2-13)	Synechococcus		Chroococcales		Cyanobacteria	Bacteria
YP_474942.1	Synechococcus sp. JA-3-3Ab	Synechococcus		Chroococcales		Cyanobacteria	Bacteria
YP_001733987.1	Synechococcus sp. PCC 7002	Synechococcus		Chroococcales		Cyanobacteria	Bacteria
YP_001734418.1	Synechococcus sp. PCC 7002	Synechococcus		Chroococcales		Cyanobacteria	Bacteria
YP_001228191.1	Synechococcus sp. RCC307	Synechococcus		Chroococcales		Cyanobacteria	Bacteria
YP_001226927.1	Synechococcus sp. RCC307	Synechococcus		Chroococcales		Cyanobacteria	Bacteria
YP_001224437.1	Synechococcus sp. WH 7803	Synechococcus		Chroococcales		Cyanobacteria	Bacteria
YP_001225595.1	Synechococcus sp. WH 7803	Synechococcus		Chroococcales		Cyanobacteria	Bacteria
NP_897622.1	Synechococcus sp. WH 8102	Synechococcus		Chroococcales		Cyanobacteria	Bacteria
NP_897953.1	Synechococcus sp. WH 8102	Synechococcus		Chroococcales		Cyanobacteria	Bacteria
NP_440609.1	Synechocystis sp. PCC 6803	Synechocystis		Chroococcales		Cyanobacteria	Bacteria
NP_441291.1	Synechocystis sp. PCC 6803	Synechocystis		Chroococcales		Cyanobacteria	Bacteria
NP_442983.1	Synechocystis sp. PCC 6803	Synechocystis		Chroococcales		Cyanobacteria	Bacteria
YP_847042.1	Syntrophobacter fumaroxidans	Syntrophobacter	Syntrophobacteraceae	Syntrophobacterales	Deltaproteobacteria	Proteobacteria	Bacteria
ZP_02844227.1	Thauera sp. MZ1T	Thauera	Rhodocyclaceae	Rhodocyclales	Betaproteobacteria	Proteobacteria	Bacteria
ZP_02843808.1	Thauera sp. MZ1T	Thauera	Rhodocyclaceae	Rhodocyclales	Betaproteobacteria	Proteobacteria	Bacteria

**Accession**	**species**	**genus**	**order**	**classe**	**family**	**phylum**	**domain**
ZP_02841424.1	Thauera sp. MZ1T	Thauera	Rhodocyclaceae	Rhodocyclales	Betaproteobacteria	Proteobacteria	Bacteria
YP_289753.1	Thermobifida fusca	Thermobifida	Nocardiopsaceae	Actinomycetales	Actinobacteria (class)	Actinobacteria	Bacteria
YP_289077.1	Thermobifida fusca	Thermobifida	Nocardiopsaceae	Actinomycetales	Actinobacteria (class)	Actinobacteria	Bacteria
YP_288942.1	Thermobifida fusca	Thermobifida	Nocardiopsaceae	Actinomycetales	Actinobacteria (class)	Actinobacteria	Bacteria
NP_682800.1	Thermosynechococcus elongatus	Thermosynechococcus		Chroococcales		Cyanobacteria	Bacteria
BAA41041.1	Thermosynechococcus vulcanus	Thermosynechococcus		Chroococcales		Cyanobacteria	Bacteria
ZP_03134051.1	Thermus aquaticus	Thermus	Thermaceae	Thermales	Deinococci	Deinococcus-Thermus	Bacteria
YP_143578.1	Thermus thermophilus	Thermus	Thermaceae	Thermales	Deinococci	Deinococcus-Thermus	Bacteria
YP_005640.1	Thermus thermophilus	Thermus	Thermaceae	Thermales	Deinococci	Deinococcus-Thermus	Bacteria
YP_144401.1	Thermus thermophilus	Thermus	Thermaceae	Thermales	Deinococci	Deinococcus-Thermus	Bacteria
YP_004743.1	Thermus thermophilus	Thermus	Thermaceae	Thermales	Deinococci	Deinococcus-Thermus	Bacteria
AAB00370.1	Thermus thermophilus	Thermus	Thermaceae	Thermales	Deinococci	Deinococcus-Thermus	Bacteria
ZP_03278562.1	Thioalkalivibrio sp. HL-EbGR7	Thioalkalivibrio	Ectothiorhodospiraceae	Chromatiales	Gammaproteobacteria	Proteobacteria	Bacteria
ZP_03278292.1	Thioalkalivibrio sp. HL-EbGR7	Thioalkalivibrio	Ectothiorhodospiraceae	Chromatiales	Gammaproteobacteria	Proteobacteria	Bacteria
ZP_03278572.1	Thioalkalivibrio sp. HL-EbGR7	Thioalkalivibrio	Ectothiorhodospiraceae	Chromatiales	Gammaproteobacteria	Proteobacteria	Bacteria
YP_314084.1	Thiobacillus denitrificans	Thiobacillus	Hydrogenophilaceae	Hydrogenophilales	Betaproteobacteria	Proteobacteria	Bacteria
YP_314401.1	Thiobacillus denitrificans	Thiobacillus	Hydrogenophilaceae	Hydrogenophilales	Betaproteobacteria	Proteobacteria	Bacteria
YP_314096.1	Thiobacillus denitrificans	Thiobacillus	Hydrogenophilaceae	Hydrogenophilales	Betaproteobacteria	Proteobacteria	Bacteria
YP_314319.1	Thiobacillus denitrificans	Thiobacillus	Hydrogenophilaceae	Hydrogenophilales	Betaproteobacteria	Proteobacteria	Bacteria
YP_314581.1	Thiobacillus denitrificans	Thiobacillus	Hydrogenophilaceae	Hydrogenophilales	Betaproteobacteria	Proteobacteria	Bacteria
YP_392229.1	Thiomicrospira crunogena	Thiomicrospira	Piscirickettsiaceae	Thiotrichales	Gammaproteobacteria	Proteobacteria	Bacteria

YP_720232.1	Trichodesmium erythraeum	Trichodesmium		Oscillatoriales		Cyanobacteria	Bacteria
YP_721510.1	Trichodesmium erythraeum	Trichodesmium		Oscillatoriales		Cyanobacteria	Bacteria
NP_787372.1	Tropheryma whipplei	Tropheryma		Actinomycetales	Actinobacteria (class)	Actinobacteria	Bacteria
NP_789455.1	Tropheryma whipplei	Tropheryma		Actinomycetales	Actinobacteria (class)	Actinobacteria	Bacteria
YP_996163.1	Verminephrobacter eiseniae	Verminephrobacter	Comamonadaceae	Burkholderiales	Betaproteobacteria	Proteobacteria	Bacteria
YP_995960.1	Verminephrobacter eiseniae	Verminephrobacter	Comamonadaceae	Burkholderiales	Betaproteobacteria	Proteobacteria	Bacteria
EDY82944.1	Verrucomicrobiae bacterium DG1235			Verrucomicrobiales	Verrucomicrobiae	Verrucomicrobia	Bacteria
EDY83083.1	Verrucomicrobiae bacterium DG1235			Verrucomicrobiales	Verrucomicrobiae	Verrucomicrobia	Bacteria
ZP_02927147.1	Verrucomicrobium spinosum	Verrucomicrobium	Verrucomicrobiaceae	Verrucomicrobiales	Verrucomicrobiae	Verrucomicrobia	Bacteria
ZP_02925765.1	Verrucomicrobium spinosum	Verrucomicrobium	Verrucomicrobiaceae	Verrucomicrobiales	Verrucomicrobiae	Verrucomicrobia	Bacteria
ZP_01262713.1	Vibrio alginolyticus	Vibrio	Vibrionaceae	Vibrionales	Gammaproteobacteria	Proteobacteria	Bacteria
ZP_01260779.1	Vibrio alginolyticus	Vibrio	Vibrionaceae	Vibrionales	Gammaproteobacteria	Proteobacteria	Bacteria
ZP_01258960.1	Vibrio alginolyticus	Vibrio	Vibrionaceae	Vibrionales	Gammaproteobacteria	Proteobacteria	Bacteria
ZP_01237208.1	Vibrio angustum	Vibrio	Vibrionaceae	Vibrionales	Gammaproteobacteria	Proteobacteria	Bacteria
ZP_01235351.1	Vibrio angustum	Vibrio	Vibrionaceae	Vibrionales	Gammaproteobacteria	Proteobacteria	Bacteria
ZP_01234682.1	Vibrio angustum	Vibrio	Vibrionaceae	Vibrionales	Gammaproteobacteria	Proteobacteria	Bacteria
ZP_02197718.1	Vibrio campbellii	Vibrio	Vibrionaceae	Vibrionales	Gammaproteobacteria	Proteobacteria	Bacteria
ZP_02194303.1	Vibrio campbellii	Vibrio	Vibrionaceae	Vibrionales	Gammaproteobacteria	Proteobacteria	Bacteria
ZP_02194798.1	Vibrio campbellii	Vibrio	Vibrionaceae	Vibrionales	Gammaproteobacteria	Proteobacteria	Bacteria
ZP_02194286.1	Vibrio campbellii	Vibrio	Vibrionaceae	Vibrionales	Gammaproteobacteria	Proteobacteria	Bacteria
YP_001216999.1	Vibrio cholerae	Vibrio	Vibrionaceae	Vibrionales	Gammaproteobacteria	Proteobacteria	Bacteria
NP_231085.1	Vibrio cholerae	Vibrio	Vibrionaceae	Vibrionales	Gammaproteobacteria	Proteobacteria	Bacteria
ZP_01953281.1	Vibrio cholerae	Vibrio	Vibrionaceae	Vibrionales	Gammaproteobacteria	Proteobacteria	Bacteria
YP_002156096.1	Vibrio fischeri	Aliivibrio	Vibrionaceae	Vibrionales	Gammaproteobacteria	Proteobacteria	Bacteria

Accession	species	genus	order	classe	family	phylum	domain
YP_204682.1	Vibrio fischeri	Aliivibrio	Vibrionaceae	Vibrionales	Gammaproteobacteria	Proteobacteria	Bacteria
YP_001448390.1	Vibrio harveyi	Vibrio	Vibrionaceae	Vibrionales	Gammaproteobacteria	Proteobacteria	Bacteria
YP_001447623.1	Vibrio harveyi	Vibrio	Vibrionaceae	Vibrionales	Gammaproteobacteria	Proteobacteria	Bacteria
YP_001445492.1	Vibrio harveyi	Vibrio	Vibrionaceae	Vibrionales	Gammaproteobacteria	Proteobacteria	Bacteria
YP_001447597.1	Vibrio harveyi	Vibrio	Vibrionaceae	Vibrionales	Gammaproteobacteria	Proteobacteria	Bacteria
NP_800047.1	Vibrio parahaemolyticus	Vibrio	Vibrionaceae	Vibrionales	Gammaproteobacteria	Proteobacteria	Bacteria
ZP_01990060.1	Vibrio parahaemolyticus	Vibrio	Vibrionaceae	Vibrionales	Gammaproteobacteria	Proteobacteria	Bacteria
NP_800138.1	Vibrio parahaemolyticus	Vibrio	Vibrionaceae	Vibrionales	Gammaproteobacteria	Proteobacteria	Bacteria
ZP_01990224.1	Vibrio parahaemolyticus	Vibrio	Vibrionaceae	Vibrionales	Gammaproteobacteria	Proteobacteria	Bacteria
NP_797923.1	Vibrio parahaemolyticus	Vibrio	Vibrionaceae	Vibrionales	Gammaproteobacteria	Proteobacteria	Bacteria
ZP_01865541.1	Vibrio shilonii	Vibrio	Vibrionaceae	Vibrionales	Gammaproteobacteria	Proteobacteria	Bacteria
ZP_01867969.1	Vibrio shilonii	Vibrio	Vibrionaceae	Vibrionales	Gammaproteobacteria	Proteobacteria	Bacteria
ZP_01869570.1	Vibrio shilonii	Vibrio	Vibrionaceae	Vibrionales	Gammaproteobacteria	Proteobacteria	Bacteria

Accession	species	genus	order	classe	family	phylum	domain
ZP_01865726.1	Vibrio shilonii	Vibrio	Vibrionaceae	Vibrionales	Gammaproteobacteria	Proteobacteria	Bacteria
YP_002075077.1	Vibrio sp. Ex25	Vibrio	Vibrionaceae	Vibrionales	Gammaproteobacteria	Proteobacteria	Bacteria
YP_002074576.1	Vibrio sp. Ex25	Vibrio	Vibrionaceae	Vibrionales	Gammaproteobacteria	Proteobacteria	Bacteria
ZP_01064301.1	Vibrio sp. MED222	Vibrio	Vibrionaceae	Vibrionales	Gammaproteobacteria	Proteobacteria	Bacteria
ZP_01063507.1	Vibrio sp. MED222	Vibrio	Vibrionaceae	Vibrionales	Gammaproteobacteria	Proteobacteria	Bacteria
ZP_00990597.1	Vibrio splendidus	Vibrio	Vibrionaceae	Vibrionales	Gammaproteobacteria	Proteobacteria	Bacteria
ZP_00989662.1	Vibrio splendidus	Vibrio	Vibrionaceae	Vibrionales	Gammaproteobacteria	Proteobacteria	Bacteria
ZP_00988490.1	Vibrio splendidus	Vibrio	Vibrionaceae	Vibrionales	Gammaproteobacteria	Proteobacteria	Bacteria
ZP_00988835.1	Vibrio splendidus	Vibrio	Vibrionaceae	Vibrionales	Gammaproteobacteria	Proteobacteria	Bacteria
NP_937171.1	Vibrio vulnificus	Vibrio	Vibrionaceae	Vibrionales	Gammaproteobacteria	Proteobacteria	Bacteria
NP_762523.1	Vibrio vulnificus	Vibrio	Vibrionaceae	Vibrionales	Gammaproteobacteria	Proteobacteria	Bacteria
NP_934464.1	Vibrio vulnificus	Vibrio	Vibrionaceae	Vibrionales	Gammaproteobacteria	Proteobacteria	Bacteria
NP_761442.1	Vibrio vulnificus	Vibrio	Vibrionaceae	Vibrionales	Gammaproteobacteria	Proteobacteria	Bacteria
ZP_01812538.1	Vibrionales bacterium SWAT-3			Vibrionales	Gammaproteobacteria	Proteobacteria	Bacteria
ZP_01815045.1	Vibrionales bacterium SWAT-3			Vibrionales	Gammaproteobacteria	Proteobacteria	Bacteria
ZP_01815176.1	Vibrionales bacterium SWAT-3			Vibrionales	Gammaproteobacteria	Proteobacteria	Bacteria
ZP_01812411.1	Vibrionales bacterium SWAT-3			Vibrionales	Gammaproteobacteria	Proteobacteria	Bacteria
AAM20915.1	Vitreoscilla sp. C1	Vitreoscilla	Neisseriaceae	Neisseriales	Betaproteobacteria	Proteobacteria	Bacteria
NP_871151.1	Wigglesworthia glossinidia	Wigglesworthia	Enterobacteriaceae	Enterobacteriales	Gammaproteobacteria	Proteobacteria	Bacteria
YP_198138.1	Wolbachia endosym. of Brugia malayi	Wolbachia	Rickettsiaceae	Rickettsiales	Alphaproteobacteria	Proteobacteria	Bacteria
YP_001974910.1	Wolbachia endosym.of Culex quinquefasciatus	Wolbachia	Rickettsiaceae	Rickettsiales	Alphaproteobacteria	Proteobacteria	Bacteria
NP_966102.1	Wolbachia endosym.of Drosophila melanogast	Wolbachia	Rickettsiaceae	Rickettsiales	Alphaproteobacteria	Proteobacteria	Bacteria
ZP_00372535.1	Wolbachia endosym of Drosophila simulans	Wolbachia	Rickettsiaceae	Rickettsiales	Alphaproteobacteria	Proteobacteria	Bacteria
NP_906440.1	Wolinella succinogenes	Wolinella	Helicobacteraceae	Campylobacterales	Epsilonproteobacteria	Proteobacteria	Bacteria
YP_001419524.1	Xanthobacter autotrophicus	Xanthobacter	Xanthobacteraceae	Rhizobiales	Alphaproteobacteria	Proteobacteria	Bacteria
YP_001415374.1	Xanthobacter autotrophicus	Xanthobacter	Xanthobacteraceae	Rhizobiales	Alphaproteobacteria	Proteobacteria	Bacteria
YP_001417931.1	Xanthobacter autotrophicus	Xanthobacter	Xanthobacteraceae	Rhizobiales	Alphaproteobacteria	Proteobacteria	Bacteria
NP_644193.1	Xanthomonas axonopodis	Xanthomonas	Xanthomonadaceae	Xanthomonadales	Gammaproteobacteria	Proteobacteria	Bacteria
NP_641594.1	Xanthomonas axonopodis	Xanthomonas	Xanthomonadaceae	Xanthomonadales	Gammaproteobacteria	Proteobacteria	Bacteria
YP_001905423.1	Xanthomonas campestris	Xanthomonas	Xanthomonadaceae	Xanthomonadales	Gammaproteobacteria	Proteobacteria	Bacteria
NP_639177.1	Xanthomonas campestris	Xanthomonas	Xanthomonadaceae	Xanthomonadales	Gammaproteobacteria	Proteobacteria	Bacteria
YP_244964.1	Xanthomonas campestris	Xanthomonas	Xanthomonadaceae	Xanthomonadales	Gammaproteobacteria	Proteobacteria	Bacteria
YP_001904584.1	Xanthomonas campestris	Xanthomonas	Xanthomonadaceae	Xanthomonadales	Gammaproteobacteria	Proteobacteria	Bacteria
YP_244149.1	Xanthomonas campestris	Xanthomonas	Xanthomonadaceae	Xanthomonadales	Gammaproteobacteria	Proteobacteria	Bacteria
NP_636534.1	Xanthomonas campestris	Xanthomonas	Xanthomonadaceae	Xanthomonadales	Gammaproteobacteria	Proteobacteria	Bacteria
YP_365736.1	Xanthomonas euvesicatoria	Xanthomonas	Xanthomonadaceae	Xanthomonadales	Gammaproteobacteria	Proteobacteria	Bacteria
YP_363039.1	Xanthomonas euvesicatoria	Xanthomonas	Xanthomonadaceae	Xanthomonadales	Gammaproteobacteria	Proteobacteria	Bacteria
YP_452948.1	Xanthomonas oryzae	Xanthomonas	Xanthomonadaceae	Xanthomonadales	Gammaproteobacteria	Proteobacteria	Bacteria

YP_202779.1	Xanthomonas oryzae	Xanthomonas	Xanthomonadaceae	Xanthomonadales	Gammaproteobacteria	Proteobacteria	Bacteria
YP_001911827.1	Xanthomonas oryzae	Xanthomonas	Xanthomonadaceae	Xanthomonadales	Gammaproteobacteria	Proteobacteria	Bacteria
YP_001912706.1	Xanthomonas oryzae	Xanthomonas	Xanthomonadaceae	Xanthomonadales	Gammaproteobacteria	Proteobacteria	Bacteria
YP_200269.1	Xanthomonas oryzae	Xanthomonas	Xanthomonadaceae	Xanthomonadales	Gammaproteobacteria	Proteobacteria	Bacteria
YP_450545.1	Xanthomonas oryzae	Xanthomonas	Xanthomonadaceae	Xanthomonadales	Gammaproteobacteria	Proteobacteria	Bacteria
YP_001775363.1	Xylella fastidiosa	Xylella	Xanthomonadaceae	Xanthomonadales	Gammaproteobacteria	Proteobacteria	Bacteria
YP_001829371.1	Xylella fastidiosa	Xylella	Xanthomonadaceae	Xanthomonadales	Gammaproteobacteria	Proteobacteria	Bacteria
NP_778846.1	Xylella fastidiosa	Xylella	Xanthomonadaceae	Xanthomonadales	Gammaproteobacteria	Proteobacteria	Bacteria
NP_298678.1	Xylella fastidiosa	Xylella	Xanthomonadaceae	Xanthomonadales	Gammaproteobacteria	Proteobacteria	Bacteria
ZP_00682022.1	Xylella fastidiosa	Xylella	Xanthomonadaceae	Xanthomonadales	Gammaproteobacteria	Proteobacteria	Bacteria
ZP_00821016.1	Yersinia bercovieri	Yersinia	Enterobacteriaceae	Enterobacteriales	Gammaproteobacteria	Proteobacteria	Bacteria
YP_001007324.1	Yersinia enterocolitica	Yersinia	Enterobacteriaceae	Enterobacteriales	Gammaproteobacteria	Proteobacteria	Bacteria
ZP_00829851.1	Yersinia frederiksenii	Yersinia	Enterobacteriaceae	Enterobacteriales	Gammaproteobacteria	Proteobacteria	Bacteria
ZP_00834817.1	Yersinia intermedia	Yersinia	Enterobacteriaceae	Enterobacteriales	Gammaproteobacteria	Proteobacteria	Bacteria
YP_001607427.1	Yersinia pestis	Yersinia	Enterobacteriaceae	Enterobacteriales	Gammaproteobacteria	Proteobacteria	Bacteria
NP_992154.1	Yersinia pestis	Yersinia	Enterobacteriaceae	Enterobacteriales	Gammaproteobacteria	Proteobacteria	Bacteria
NP_668350.1	Yersinia pestis	Yersinia	Enterobacteriaceae	Enterobacteriales	Gammaproteobacteria	Proteobacteria	Bacteria
YP_646858.1	Yersinia pestis	Yersinia	Enterobacteriaceae	Enterobacteriales	Gammaproteobacteria	Proteobacteria	Bacteria
NP_406640.1	Yersinia pestis	Yersinia	Enterobacteriaceae	Enterobacteriales	Gammaproteobacteria	Proteobacteria	Bacteria
YP_652566.1	Yersinia pestis	Yersinia	Enterobacteriaceae	Enterobacteriales	Gammaproteobacteria	Proteobacteria	Bacteria
YP_001164135.1	Yersinia pestis	Yersinia	Enterobacteriaceae	Enterobacteriales	Gammaproteobacteria	Proteobacteria	Bacteria
YP_002348070.1	Yersinia pestis	Yersinia	Enterobacteriaceae	Enterobacteriales	Gammaproteobacteria	Proteobacteria	Bacteria
YP_001402059.1	Yersinia pseudotuberculosis	Yersinia	Enterobacteriaceae	Enterobacteriales	Gammaproteobacteria	Proteobacteria	Bacteria
YP_001721964.1	Yersinia pseudotuberculosis	Yersinia	Yersinia	Yersinia	Yersinia	Yersinia	Yersinia
YP_001871430.1	Yersinia pseudotuberculosis	Yersinia	Yersinia	Yersinia	Yersinia	Yersinia	Yersinia
YP_069492.1	Yersinia pseudotuberculosis	Yersinia	Yersinia	Yersinia	Yersinia	Yersinia	Yersinia
